# Rational search for natural antimicrobial compounds: relevance of sesquiterpene lactones

**DOI:** 10.1007/s13659-025-00513-y

**Published:** 2025-05-08

**Authors:** Alejandro Recio-Balsells, Eugenia Rodriguez Ristau, Adriana Pacciaroni, Viviana Nicotra, Carina Casero, Manuela García

**Affiliations:** 1 Instituto Multidisciplinario de Biología Vegetal (IMBIV), Consejo Nacional de Investigaciones Científicas y Técnicas (CONICET), Córdoba, Argentina; 2 Facultad de Ciencias Químicas, Universidad Nacional de Córdoba (UNC), Ciudad Universitaria, X5000HUA Córdoba, Argentina

**Keywords:** Sesquiterpene lactones, Antimicrobials, Semisynthetic derivatives, Bioguided study, Extract derivatization

## Abstract

Antimicrobial resistance is one of the most pressing global health challenges, as many pathogens are rapidly evolving to evade existing treatments. Despite this urgent need for new solutions, natural plant-derived compounds remain relatively underexplored in the development of antimicrobial drugs. This report highlights an innovative approach to discovering potent antimicrobial agents through bioguided fractionation of numerous plant species from the rich Argentinean flora. By systematically screening 60 species (over 177 extracts) for antimicrobial activity against representative strains of gram-positive and gram-negative bacteria, we identified promising bioactive compounds within the Asteraceae family—particularly sesquiterpene lactones from the *Xanthium* genus. Building on this basis, we synthesized semi-synthetic derivatives by chemically modifying plant sub-extracts, focusing on structures incorporating heteroatoms and/or heterocycles containing oxygen and nitrogen (important for the bioavailability and bioactivity that they are capable of providing). These modifications were evaluated for their potential to enhance antimicrobial efficacy against bacteria and *Candida* species, including resistant strains. Our findings suggest that tailoring natural metabolites from *Xanthium* and related Asteraceae species can significantly improve their antimicrobial properties. This strategy offers a promising pathway for the development of novel therapeutic agents to combat bacterial and fungal infections in an era of rising drug resistance.

**Graphical Abstract:**

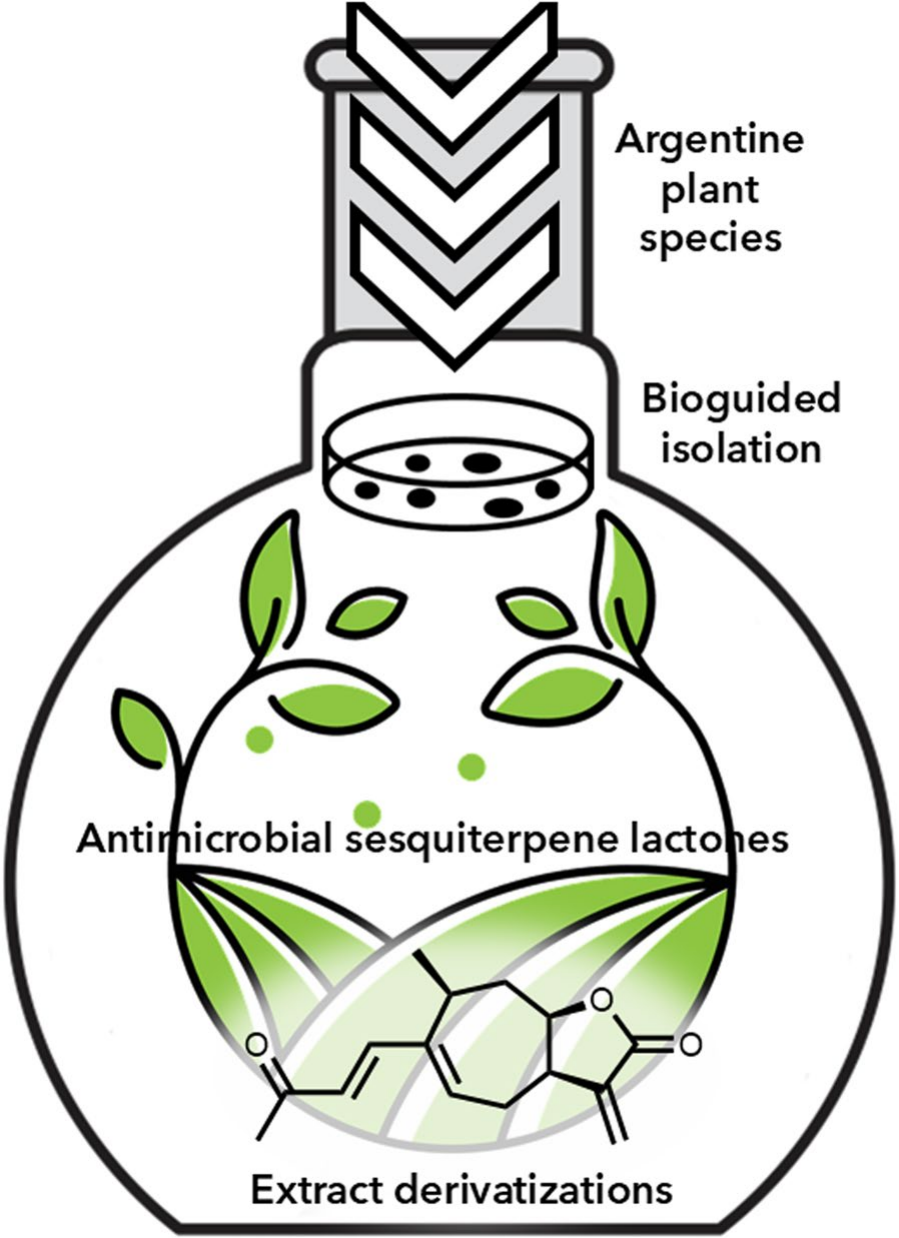

**Supplementary Information:**

The online version contains supplementary material available at 10.1007/s13659-025-00513-y.

## Introduction

Microbial resistance strains are a growing global public health problem. The emergence of these resistant strains is primarily linked to misuse of them, lack of adherence to treatment and excessive use of antimicrobials in humans, animals, and plants [1]. Bacterial antimicrobial resistance (AMR) was estimated to contribute to 4.95 million deaths worldwide in 2019 and to be directly responsible for 1.27 million of these deaths [2]. This ever-expanding and worrying situation allows for the projection of alarming numbers of victims in the future. If appropriate actions are not taken immediately, AMR is projected to kill more people per year than cancer by 2050, with an estimated 10 million deaths annually at a cost of USD 100 trillion to the global economy [3]. Adding to these alarming statistics is the fact that the pharmaceutical industry has redirected its investments away from developing new antimicrobial agents, prioritizing more lucrative areas like chronic diseases [4]. This situation highlights the urgent need for coordinated efforts to address this growing crisis and the imperative to invest in the research and development of new antimicrobial drugs.

Within the complex landscape of antimicrobial resistance, the ESKAPE bacteria group stands out as a significant contributor to the ongoing challenge, causing nosocomial infections and presenting clinically isolated multidrug resistance strains [5]. The ESKAPE group includes both Gram-positive (GP) bacteria, *Enterococcus faecium* and *Staphylococcus aureus*, and Gram-negative (GN) bacteria such as *Klebsiella pneumoniae*, *Acinetobacter baumannii*, *Pseudomonas aeruginosa,* and *Enterobacter spp.*

Regarding fungal microbes, invasive fungal infections have an important threat to public health and are an under-recognized component of antimicrobial resistance. Systemic fungal infections primarily affect immunosuppressed individuals, such as those with HIV/AIDS or undergoing cancer treatment. The main pathogens involved are *Candida*, *Cryptococcus* and *Aspergillus* species [6]. *Candida albicans*, a commensal of the human microbiota, is the most common cause of invasive candidiasis, which can be fatal in vulnerable patients, with mortality rates exceeding 40% [7]. The development of new antifungals drugs advances slowly due to the evolutionary similarity between fungi (eukaryotic) and humans host (mammalians), limiting drug targets. Historically, treatment has relied heavily on just four classes of systemically acting antifungal drugs; this small number of therapeutic options raises great concern due to increasing drug resistance [8].

Historically, natural products (NPs) have played a key role in drug discovery, especially for cancer and infectious diseases, but also in other therapeutic areas, including cardiovascular and neurological diseases. The potential of NPs in drug discovery is unquestionable. Of the drugs approved between 1981 and 2019, nearly half of the new chemical entities approved for therapeutic use are of natural origin, derived from them, or inspired by them [9]. These molecules have undergone a process of evolutionary diversification, allowing them to bind to specific cellular targets, in some cases favoring active or passive membrane permeation. In comparison to small synthetic molecules, NPs cover a wide region of chemical space due to their high structural complexity and inherent scaffold diversity [10]. NPs exhibited distinct features involving more stereochemical centers, greater higher tridimensional shape, carbon-sp^3^ fraction and a more oxygenated structure [11]. The extraordinary success of NPs as drug leads is most evident in the field of antimicrobials, constituting a very rich source of anti-infective compounds, either as such or as potent semisynthetic variations of them. In different data analyses carried out by Porras et al*.* and Álvarez-Martínez et al*.*, phenolic derivates and terpenoids emerge as the predominant antibacterial metabolites derived from plants [12, 13]. Moreover, this class of NPs also demonstrate to be crucial sources of antifungal agents, yielding nine new leads from 2010 to 2019 [14, 15]. Within the terpenoid cluster, sesquiterpene lactones, predominantly obtained from the Asteraceae family, present a C-15 structure formed by three isoprenoid units. These compounds typically incorporate a γ-butyrolactone ring, often α,β-unsaturated with an exocyclic alkene. Notably, this class of compounds has shown a broad spectrum of bioactivities, including anti-infective, antitumoral properties, anti-inflammatory, among others [16].

A strategy used in the search for bioactive compounds involves the chemical modification of plant extracts by introducing functionalities that are rarely produced by secondary metabolism [17]. In this way, the biosynthetic machinery of nature can be complemented to produce a whole range of new semisynthetic compounds in a single reaction step. This strategy has been scarcely utilized in the search for antibacterial compounds.

In response to the urgent demand for new GN antimicrobial agents, several specific approaches have been explored using pure isolated natural compounds to address this challenge. One strategy consists in a synthetic approach that focuses on the nitrogen enrichment of compounds, promoting their accumulation in GN bacteria. This approach is guided by the “eNTRy rules”, which were established through a data-driven experiment that examined the accumulation of approximately 180 compounds in *E. coli*. According to this rule, the probability of compound accumulation in GN increased with the presence of nitrogen groups, especially primary amines, low three-dimensionality (Globularity ≤ 0.25) and rigidity (rotable bonds ≤ 5) [18, 19]. One successful example, is the contribution of Onyedibe et al., in which the addition of a primary amine to a tetrahydrobenzo[α]acridines derivative with GP activity led to the acquisition of GN activity [20].

The search for bioactive compounds through the screening of plant extracts or semisynthetic mixtures can be addressed through a bioguided approach or biodirected fractionation. In this strategy, only those extracts and/or fractions that show activity are selected and their purification are carried out until the pure compounds responsible for the biological activity are obtained. Currently, bioguided phytochemical studies are the most common due to their greater efficiency in terms of time and resources. However, the variable concentrations of compounds in plant extracts, as well as additive, synergistic and antagonistic interactions among them, must be considered [21].

In this study, was explored the antimicrobial potential of the ethanol (EtOH), hexane (Hex) and ethyl acetate (EtAcO) extracts from 60 species of Argentine flora, through a combined bioguided approach (biological activity and NMR content profile as filter criterion). This screening was performed on ATCC bacterial strains *S. aureus* and *E. coli*.

This working methodology led to the isolation of the pure compounds responsible for antimicrobial activity. Another scope of this work involved the chemical derivatization of the extracts with good antimicrobial activity in search of more active compounds. Some strategies involving nitrogen enrichment to improve the permeation of compounds in GN bacteria were analyzed. Furthermore, for the most promising antimicrobial compounds, biological assays were expanded to include strains of methicillin-resistant *S. aureus* (MRSA), *C. tropicalis* and *C. neoformans*. In addition, the modulation of the bioactivity of natural compounds or derivatives with fluconazole, amphotericin B, linezolid and ampicillin were evaluated.

## Results and discussion

As part of our search for bioactive compounds from plant biodiversity, almost 60 native plant species of Argentina, belonging to 22 different families, were processed (Table S1, Supplementary information). In all cases, the aerial parts of each plant sample (except the use of roots of *Solidago chilensis*) were air-dried and extracted with EtOH. After concentration, the residue was partitioned with *n*-hexane and subsequently with EtOAc. A sample of each organic extract was taken, and its antimicrobial potential against two clinically important bacterial species, *S. aureus* methicillin-sensitive ATCC 25923 (MSSA) and *E. coli* ATCC 25922, was evaluated using the broth microdilution method [22].

To proceed with a bioguided isolation approach and for biomonitoring purposes, the concept of the cut-off point proposed by Machado et al. was adopted, so that extracts and fractions with MICs greater than 1000 μg/mL were considered inactive. In this way, the results of antibacterial activity revealed that of the 177 extracts evaluated, 26 exhibited inhibitory effect on the growth of *S. aureus*, while none was effective against *E. coli.* Regarding the plant families, most of the bioactive extracts were obtained from species belonging to Asteraceae. Among the active extracts, 13 were obtained of partition with EtOAc (50%), 9 with EtOH (35%) and 4 with Hex (15%), see Table S1 in Supplementary Information.

These results highlight the potential efficacy of EtOAc extracts over their EtOH and Hex counterparts. In the total ethanolic extracts the bioactive metabolites of interest are possibly very diluted, therefore, for future high-throughput bioguided testing of plant-derived extracts, partitions that allow concentration and thus detection of compounds present in low concentrations are necessary. Discarding total ethanolic extracts may lead to the elimination of valuable extracts.

It is important to clarify that the inactivity of the extracts analyzed does not imply an absence of bioactive molecules, simply that the workflow anticipates that a criterion be taken to advance with the purification that allows arriving at the pure compounds and/or mixtures responsible for the antimicrobial activity. In fact, the presence of hundreds of compounds within a global extract or the low relative concentration of a certain group of metabolites often leads to such results.

After a detailed review of the bibliographical background of the bioactive species, the bioguided approach was continued on the panel of active EtOAc extracts namely, *Zinnia peruviana*, [23] *Laurus nobilis*, [24]*, Hypochaeris radicata*, [25] *Xanthium cavanillesii* [26] and *Xanthium spinosum* [27]*.* Interestingly, when analyzing the phytochemical background of the selected species, it was observed that they have different sesquiterpene lactone nuclei as the major specialized metabolite contained (supplemented by ^1^H-NMR analysis) in the EtOAc polar extract. Of the selected plant material, previous phytochemical studies were performed.

Therefore, the selected extracts were fractionated by vacuum chromatography and grouped into four fractions (F1-F4). The results of the MIC determination for selected bioactive extracts and their fractions against *S. aureus* are summarized in Table S2.

Globally, considering a) the bioactivity values obtained, b) the reports of sesquiterpene lactones as metabolites with promising antimicrobial activity, c) the high amount of individual metabolites in the *Xanthium* species (supplemented by ^1^H-NMR analysis), and d) the complexity of the mixture, i.e. the feasibility for subsequent purifications (by the complementary use of ^1^H-NMR and TLC), *X. cavanillesii* and *X. spinosum* were selected to continue the isolation of antimicrobial metabolites. Indeed, the selection of these species also enables the implementation of several derivatization strategies (from pure compounds or from crude extracts) and the potential access to a wide structural diversity for the establishment of structure–activity relationships.

**Fig.1:**
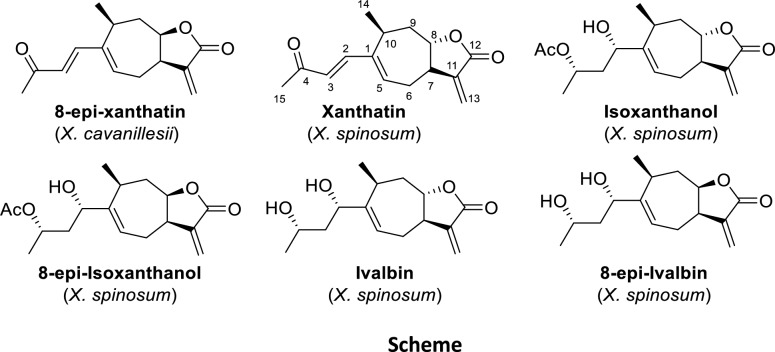
Natural compounds isolated from *Xanthium* sp.

The isolation of the compounds responsible for the activity of the EtOAc extract of *X. cavanillesii* proved to be remarkably straightforward. The main compound isolated from the bioactive fractions (F2 and F3, MIC = 0.5 mg/mL against *S. aureus*) of *X. cavanillesii* was 8-*epi*-xanthatin [28], constituting 46% of the total mass of the processed extract (LSs enrichment), Fig. 1.

*X. spinosum* presented a more complex constitution, their preparative TLC purification of the bioactive fractions (F2 and F3, MIC = 0.25 mg/mL against *S. aureus*) led to the isolation of 8-*epi*-isoxanthanol, 8-*epi*-ivalbine and the known compounds xanthatin [27], isoxanthanol and ivalbine [29] (Fig. 1) identified by comparison with published spectroscopic and other physical data (see ^1^H and ^13^C NMR data in Supporting Information). In this case, unlike the previous work of Olivaro et al., [26] 8-*epi*-xanthatin and non-hydroxylated xanthanolides were obtained from *X. cavanillesii*. It is necessary to mention the importance of determining the stereochemistry of the lactone ring, since there are slight differences in the chemical shifts between xanthine and 8-*epi*-xanthine that can lead to an error in the identification of the isolated metabolite (see experimental part and complementary information for structural elucidation). It is well known that phytochemical composition of different populations of the same species can differ because of a wide range of geographical, ecological and environmental reasons including, hydric stress, time of harvest, interactions between other plants or insects among others [30–32]. Therefore, indirectly, this study also raises implications from a phytochemical point of view.

In order to provide information on the possible broad-spectrum or selectivity antimicrobial activity, the antimicrobial potential of metabolites isolated from *Xanthium* spp. was evaluated against the bacterial strains used in previous biological assays and the yeast strain *C. albicans* ATCC10231. The results of the MIC determination for metabolites tested and for positive controls (ampicillin and ketoconazole) are summarized in Table 1.

**Table 1:** MIC of xanthanolides (mg/mL)

Isolated compounds	*S. aureus* ATCC 25923	*E. coli* ATCC 25922	*C. albicans* ATCC 10231
8-*epi*-xanthatin	0.50–0.25	> 1	0.25–0.125
Xanthatin	0.50–0.25	> 1	0.25–0.125
Isoxanthanol	0.25–0.125	> 1	1
8-*epi*-isoxanthanol	0.125	> 1	> 1
Ivalbin	> 1	> 1	> 1
8-*epi*-ivalbin	> 1	> 1	> 1
Ampicillin	1.6 × 10^–4^	5 × 10^–2^	NT
Ketoconazole	NT	NT	3.4 × 10^–3^

NT: Not tested

Regarding the antibacterial activity of isolated pure compounds, xanthatin, 8-*epi*-xanthatin, isoxanthanol and 8-*epi*-isoxanthanol showed similar inhibitory effect on visible growth of *S. aur*eus, with MIC values in the range of 0.50–0.125 mg/mL. None of the metabolites evaluated were effective against *E. coli*, even at the highest concentration evaluated (1 mg/mL).

In line with those described above, previous biological studies reported antibacterial and antifungal activity of xanthatin. Tsankova et al. reported an MIC value of 0.125mg/mL against *S. aureus* by a method of serial dilution in broth [33]. Sato et al. determined the activity of this metabolite against *S. aureus* strains, including *S. aureus* methicillin-resistant by disc-diffusion tests [34].

On the other hand, the similar bioactivity values obtained for xanthatin, isoxanthanol and their respective epimers demonstrated that stereochemistry at the C-8 position does not have an important compromise in the antibacterial effect. Previous biological studies reported antibacterial and antifungal activity of xanthatin. Regarding the inhibition of *C. albicans*, only xanthatin and 8-*epi*-xanthatin were bioactive, with relevant MIC values (0.25–0.125 mg/mL). This result agreed with those obtained by Lavault et al., who reported a similar inhibitory effect for xanthatin against *C. albicans* and *C. glabrata* (MIC = 0.32 mg/mL) [35]. There are no previous reports of antifungal activity for 8-*epi*-xanthatin.

Relate to ivalbine and 8-*epi*-ivalbine, no inhibitory effect on the growth of the tested microbial strains was observed, even at the highest concentration tested (1 mg/mL).

Once the isolation and characterization of pure bioactive compounds from *Xanthium* spp. was completed, and given the bioactivity results obtained previously, different chemical derivatization strategies to create chemical diversity from bioactive scaffolds were explored. Given the considerable amount of 8-*epi*-xanthatin present in the EtOAc sub-extract of *X. cavanillesii*, efforts were focused on derivatization in this extract. The goal with these derivatizations to potentially improve activity was twofold: first, to introduce nitrogen heteroatoms according to the eNTRy rules and to employ oxidative reactions.

**Scheme 1:**
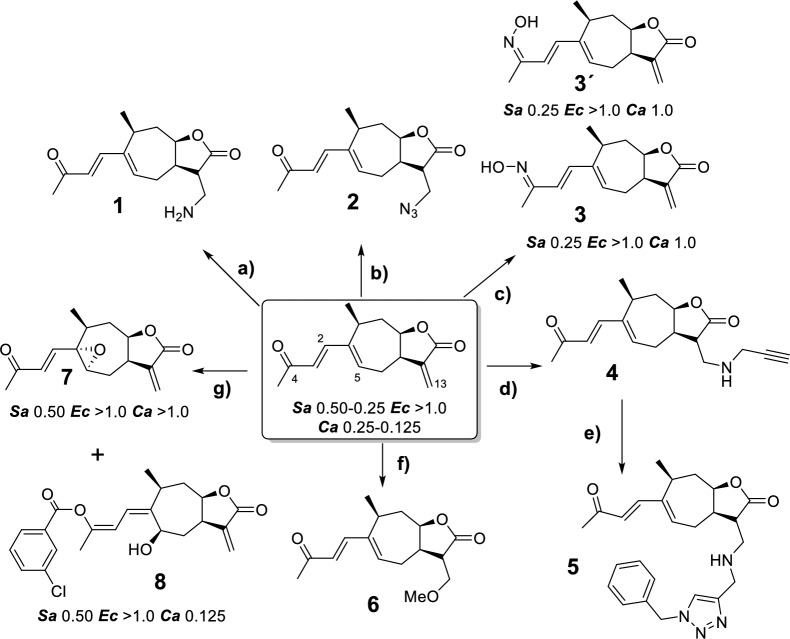
Chemical derivatization of *X. cavanillesii* EtOAc extract. Conditions**:** **a** NH_4_OH, RT, 4 h. **b** TMSA, AcOH, TEA, DCM, RT, 24 h. **c** [NH_3_OH]Cl, MeOH, RT, 24 h. **d** Propargylamine, MeOH, RT, 24 h. **e** CuSO_4,_ AscNa, H_2_O:*t*-BuOH (1:1), RT, 24 h. **f** KOH, MeOH, RT, 3 h **g** *m*CPBA, 0°C-RT, DCM, 24 h. All biological activities are MIC express in mg/mL. *Sa: S. aureus. Ec: E. coli. Ca: C. albicans*. Results are only shown for those compounds that have shown MIC in any of the strains evaluated

Scheme 1 shows the derivatives obtained from the EtOAc extract of *X. cavanillesii*, the reaction conditions and the corresponding MIC values obtained against the bacterial and yeast strains evaluated. Results are only shown for those compounds that have shown activity in any of the strains evaluated.

A series of Michael additions were conducted using different nitrogen nucleophiles, including ammonium hydroxide (**1**), trimethylsilyl azide (**2**), hydroxylammonium chloride (**3**) and propargylamine (**4**). While amine addition with ammonium hydroxide was achieved, the yield was notably low (7%), likely due to polymerization products. Reactions with trimethylsilyl azide and propargylamine yielded the expected products **2** and **4** with higher efficiencies of 24% and 56%. Given the low yield for compound **1**, it may be beneficial to pursue azide reduction from compound** 2**, though selectivity is needed to preserve the γ-lactone ring, carbonyl, and alkene groups from (in pharmacophore scaffold).

With hydroxylammonium chloride, two oxime products were obtained (**3** isomer E and **3′** isomer Z) instead of undergoing a Michael addition. These products were obtained in comparable yields of 26% for **3** and 32% for **3′**. Notably, the oxime configuration produced significant shifts in the NMR spectra, with the most pronounced difference observed in the ^1^H NMR signal for the H-3 and H-5 (6.22 ppm, 6.92 ppm respectively) for **3** and (6.96 ppm, 6.00 ppm respectively) for **3′**. Michael additions and oxime formation had been previously reported by the Yang group on xanthatin [36, 37]. The ^1^H and ^13^C-NMR chemical shifts of **3** and **3´** are in agree with those previously described for isomeric oximes synthesized from xanthatin (characterized by X-ray and NMR).

An additional approach involved a copper-catalyzed cycloaddition of the alkyne product with benzyl azide, yielding the respective 1,2,3-triazole derivative (**5**), with the aim of enhancing drug-target interactions. It is well known that triazole-based derivatives possess fascinating pharmacological features as they possess an electron-rich nature capable of allowing binding with various biological targets and enzymes, thus displaying a wide range of biological activities [38]. Similarly, a methoxy group was also introduced via Michael addition on the ɣ-lactone alkene of 8-*epi*-xanthatin. Finally, oxidation with *m*CPBA provided the corresponding epoxide (**7**), along with a product with the *m*-chlorobenzoic acid fragment (**8**).

According to the MIC values obtained against bacterial and yeast strains tested, some structure–activity relationships can be inferred. In this sense, the bioactivity values observed for the Michael-type derivatives at C-13 (compounds **1**, **2**, **4**–**6**) indicate a deleterious effect when the exocyclic double bond is replaced. Given the results obtained, it is important to note that it was not possible to evaluate the effect of the addition of nitrogen, since even complying with all the eNTRY rules (for example compound **1,** see Supplementary Information, Table S4), it is essential to preserve the natural pharmacophoric fragment intact. The addition of nucleophilic nitrogen presupposes (without the use of catalysts) a Michael addition on the exocyclic double bond, with a deleterious effect on biological activity. In this sense, it is difficult to moderate the reactivity of the exocyclic double bond for the insertion of nitrogenous nucleophiles at remote sites of the molecule, without the use of organometallic catalysis (for example, C-H amination implemented by Castro et al.) [39].

Contrary to what might be assumed, the type of fusion or ring closure of the pharmacophore supported by the configuration change at C-8 position in all NPs evaluated, is not decisive for the activity suggesting that the reactivity provided by the electrophile through its electron density is more important than the three-dimensionality effects of the electrophile group. Regarding the inhibitory effect on the growth of *S. aureus*, derivatives **3**, **3′**, **7** and **8**, whose pharmacophoric scaffold is not altered, showed bioactivity values comparable to xanthatin (MIC = 0.5–0.25 mg/mL). If the antifungal activity of the derivatives obtained is analyzed, the direct comparison of MIC values of xanthatin (0.25–0.125 mg/mL) and **7** (> 1.0 mg/mL) against *C. albicans*, allows to presume the importance of the presence of the double bond at C1–C5 in bioactivity. Overall, one explanation for these observations is that the side chain must retain some rigidity provided by the conjugated unsaturations (see Supporting information, Fig.S1 for tridimensional structures). In this sense, ivalbine, with a flexible chain and both hydroxyl groups free, did not show relevant inhibition values, perhaps because the two nearby hydroxy groups interact with each other, affecting a key compound-ligand interaction and/or by a decrease in membrane permeability.

In order to further analyze the antimicrobial behavior of these hits, the minimum bactericidal and fungicidal concentrations (MBC, MFC) of the compounds that showed an inhibitory effect on the visible growth of reference strains tested were evaluated. Thus, compounds xanthatin, 8-*epi*-xanthatin, isoxanthanol and 8-*epi*-isoxanthanol, and the derivatives **3**, **3′**, **7** and **8** were analyzed against sensitive and resistant *S. aureus* strains as well as against the reference yeast strains *C. tropicalis* ATCC 66029 and *C. neoformans* ATCC66031. The results obtained are summarized in Table 2. The direct comparison of the MIC and MBC values for all the compounds evaluated revealed a similar or greater activity against the MRSA strain compared to the sensitive strain. Regarding the antifungal activity, the bioactivity obtained showed the importance of added structural rigidity due to extended conjugation in the side chain (compound **8**). In this sense, the conservation of the activity against *C. albicans* and *C. tropicalis*, highlighting the promising bioactivity on *C. neoformans* (MIC and CFM = 0.06 and 0.125 mg/mL, respectively).

**Table 2:** MIC, MBC and MFC of naturally xanthanolides and semisynthetic derivatives **3**, **3′**,** 7** and **8** (mg/mL)

Compounds	*Sa*		*MRSA*		*Ca*		*Ct*		*Cn*	
	MIC	MBC	MIC	MBC	MIC	MFC	MIC	MFC	MIC	MFC
8-*epi*-xanthatin	0.50–0.25	1.0–0.5	0.25	0.50	0.25–0.125	0.50	0.25	0.50	0.06–0.03	0.125
Xanthatin	0.50–0.25	0.5	0.125	0.25	0.25–0.125	0.25–0.125	0.25–0.125	0.25	0.06–0.03	0.125
Isoxanthanol	0.25–0.125	1.0	0.25–0.125	0.50	1	> 1	NT	NT	NT	NT
8-*epi*-isoxanthanol	0.125	1.0	0.125	0.25	> 1	NT	NT	NT	NT	NT
**3**	0.25	1.0	0.125	0.5	1.0	> 1	> 1	NT	> 1	NT
**3′**	0.25	1.0	0.25–0.125	1.0	1.0	1.0	1.0	1.0	0.50	0.50
**7**	0.50	> 1.0	0.50	1.0	> 1	NT	NT	NT	NT	NT
**8**	0.50	> 1.0	0.50	> 1.0	0.125	0.125	0.25–0.125	0.50	0.06–0.03	0.125
Ampicillin	1.6 × 10^–4^	NT	3 × 10^–3^	NT	NT	NT	NT	NT	NT	NT
Ketoconazole	NT	NT	NT	NT	3.8 × 10^–3^	NT	3.4 × 10^–3^	NT	6 × 10^–4^	NT

NT: Not tested

Antimicrobial formulations, in which two or more bioactive compounds with different mechanisms of action are mixed, constitute an interesting approach to enhance antibacterial and antifungal efficacy, while representing a strategy that decreases the possibility of microbial resistance emergence. A large number of plant extracts and isolated NPs have been reported to act synergistically with commercial antibiotics, antifungals and chemotherapeutics, increasing the activity of these drugs, as indicated by the significant decrease in minimum inhibitory concentrations [40].

In this context, the modulatory effect of 8-*epi*-xanthatin on the activity of antimicrobial drugs with different mechanisms of action was evaluated. To this purpose, the MICs of ampicillin, linezolid (antibacterial drugs), ketoconazole, and amphotericin B (antifungal drugs) were determined in the absence and presence of this metabolite at a subinhibitory concentration (MIC/2 = 125 mg/mL). The microbial strains used in the modulation assays were *S. aureus* and *C. albicans*. Regarding the antibacterial agents evaluated, the results showed a non-significant reduction in the MIC of ampicillin against the *S. aureus* strain (MIC_ampic._ = 6.3 × 10^–3^ mg/mL; MIC_comb._ = 3.1 × 10^–3^ mg/mL). Furthermore, the combination of this metabolite with linezolid did not show a modulatory effect [MIC_lin._ = MIC_comb._ (5 × 10^–4^ mg/mL)]. Regarding the antifungal drugs evaluated, the results showed “indifference” for the combination with amphotericin B against the *C. albicans* strain. On the other hand, the MIC values obtained for the combination of ketoconazole with 8-*epi*-xanthatin showed an antagonistic effect against the same fungal strain (MIC_ketoc._ = 6 × 10^–3^ mg/mL; MIC_comb._ = 4 × 10^–1^ mg/mL).

There are several reports on the antimicrobial activity of simple natural phenols and acids, particularly regarding the antifungal activity of cinnamic acid and several related compounds. In addition, their chemosensitizing capacity has been reported, increasing the efficacy of cell wall-targeting antifungals [41]. In this context, the inhibitory activity of cinnamic acid and 4-oxo-4-(p-tolyl)butanoic acid against the strain *C. albicans* ATCC 10231 was determined, obtaining MIC values of 0.25 and 0.5 mg/mL, respectively.

**Scheme 2:**
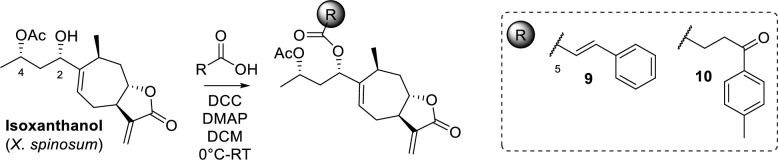
Isoxanthanol esterification

Based on this result, isoxanthanol was selected as an esterifiable and bioactive metabolite to evaluate its inhibitory potential against *C. albicans* in combination with the above-mentioned acids at sub-inhibitory concentrations (MIC/2), Scheme 2. The direct comparison of the MIC values obtained showed indifference for the combinations tested and a decrease in the inhibitory activity of derivatives **9** and **10** (MIC > 1 mg/mL) compared to the starting metabolite (MIC = 1 mg/mL, see Supporting Information Table S5 for activity data). Although these results are not as expected, they serve to determine the feasibility of combining NCEs with antimicrobials approved for use, which constitute viable approaches to reducing the doses applied.

Finally, the family of natural and semisynthetic compounds obtained in this work was evaluated on the SwissADME web platform based on the relevant physicochemical parameters of solubility and permeability with respect to oral bioavailability. All were found to comply with the Lipinsky rule and drug-likeness by acceptable oral availability predictions (see supplementary information, Table S6 and S7).

## Conclusions

The present study has allowed the exploration of a significant number of plant species of the Argentine flora, which is valuable considering the importance of NPs in the search for bioactive compounds and the low percentage of plant species studied to date. For this reason, work is currently being done on the analysis of the chemical content and bioactivity of the promising species from the initial screening, together with species not studied at the moment.

On the other hand, the importance of performing an initial fractionation of the total plant extract has been demonstrated, in order to concentrate the bioactive metabolites in a particular fraction according to their polarity. This strategy allowed the detection of bioactive metabolites that could go unnoticed because they are present in traces in the total extract.

Regarding the derivatization strategy, the efficiency achieved, in terms of time and resources, has been demonstrated by derivatizing an extract enriched in metabolites with potential bioactivity and the subsequent purification of the bioactive metabolites/derivatives. Derivatization of an extract or sub-extract allows rapid access to chemical libraries, to introduce greater chemical complexity in a single reaction step, thus rapidly expanding the possibilities of obtaining bioactive compounds.

Respecting the biological activity found, the unreported antifungal activity of 8-*epi*-xanthatin and derivative **8** against opportunistic *Candida* strains stands out. The results constitute a starting point for the future rational development of new derivatives and hybrids with improved antifungal properties. The bioactivity obtained shows the importance of added rigidity due to the extended conjugation in the side chain (compound **8**). Regarding the complexification of the structure, more in-depth studies should be carried out with different substituents in the side chain to find evidence of the molecular determinants necessary in these positions.

Finally, the analysis of the structure–activity relationships of the compounds, pharmacokinetic parameters, solubility and antimicrobial activity, have allowed us to find molecular determinants required for the synthesis of increasingly efficient derivatives. It is crucial to find new synthetic functionalization strategies that allow the obtaining of nitrogenous or oxygenated derivatives in positions far from the pharmacophoric fragment since the results obtained in resistant and fungal strains are promising. For all these reasons, these findings are the beginning of future projects related to obtaining antimicrobial SLs.

## Experimental

### General

Optical rotation was measured on a JASCO P-1010 polarimeter. IR spectra were obtained in a Nicolet iZ10 Thermo Scientific spectrophotometer (each compound was dissolved in a minimum amount of solvent, and a drop of solution was added to the AgCl IR plates). NMR experiments were performed on a Bruker AVANCE II 400 MHz instrument. Multiplicity determinations (HSQC-DEPT) and 2D spectra (COSY, HSQC, HMBC, and NOESY) were obtained using standard Bruker software. Chemical shifts are expressed in ppm (δ) units using tetramethylsilane as the standard. Exact mass spectra were obtained on a Bruker microTOF-Q II mass spectrometer, equipped with an ESI source operating in positive mode. Antimicrobial activity measurements were determined in a microplate absorbance reader (Synergy HT- Biotek. 2012). Chromatographic separations were performed by column chromatography and vacuum on silica gel 60 (0.063–0.200 mm) and preparative TLC on silica gel 60 F_254_ (0.2 mm thick) plates. The presence of compounds was revealed by anisaldehyde reagents.

### Plant material

Plants were collected in different locations of Córdoba Province, Argentina, from November 2021 to March 2022. Plants were selected according to their availability, accessibility, and previous reports on antimicrobial activity or chemical content. The plant material was identified by Gloria Barboza (IMBIV-CONICET, Córdoba, Argentina), and a voucher specimen was deposited on the Museo Botánico de Córdoba, Universidad Nacional de Córdoba (under the herbarium codes shown in the Table S1 in Supplementary Information).

### Crude extracts and fractionation

The different vegetal materials collected were air-dried, powdered, and extracted at room temperature by three extraction cycles by EtOH 96% (10 g of vegetal powder in 200 mL × 3) and the solvent was evaporated under reduced pressure. Afterwards, 200 mg of extracts were resuspended in 100 mL water (8:2) and partitioned with n-hexane (3 × 80 mL). Then the aqueous phase was partitioned and extracted with EtOAc (3 × 80 mL). Finally, the hexane and EtOAc were independently dried (Na_2_SO_4_), filtered, and evaporated to dryness.

The selected EtOAc plant extracts with promising antimicrobial activity were fractionated by flash chromatography. Elution with n-hexane/ EtOAc mixtures of increasing polarity (100:0–0:100) and EtOAc/MeOH (100:0–0:100) were grouped in four fractions (F1-F4). The structures of the compounds were determined by a combination of 1- and 2-dimensional NMR spectroscopic methods, together with the exact masses and isotopic distribution using HRMS spectrometry. All compounds were determined to be > 95% pure by ^1^H NMR spectroscopy. See supplementary information for detailed NMR data and structural elucidation.

### Isolation of metabolites from *Xanthium* species

#### Isolation of xanthanolides from *X. spinosum*

774 g of *X. spinosum* branches conditioned and extracted as described above yielded 94 g of dry extract. Partitioning this residue with n-hexane and EtOAc yielded 15.7 g and 32.9 g, respectively. Subsequently, in order to explore the chemical content (in terms of yield), 25 mg of EtOAc extract was subjected to preparative TLC purification with a mixture of EtOAc and Hex (3:1) resulting in 5.6 mg of xanthatin, 2.9 mg of isoxanthanol, 2.0 mg of 8-*epi*-isoxanthanol, 2.1 mg of ivalbin and 1.6 mg of 8-*epi*-ivalbin.

**Xanthatin:** ^1^H NMR (CDCl_3_, 400.13 MHz): 7.07 d (1H, *J* = 16.0 Hz, H-2), 6.28 dd (1H, *J* = 9.2; 3.4 Hz, H-5), 6.20 d (1H*, J* = 16.0 Hz, H-3), 6.20 d (1H, *J* = 3.0 Hz, H_2_-13a), 5.48 d (1H, *J* = 3.0 Hz, H_2_-13b), 4.29 ddd (1H, *J* = 12.3; 10.1; 2.6 Hz, H-8),3.08 m (1H*,* H-10), 2.79 ddd (1H, *J* = 16.7; 9.0; 2.6 Hz, H_2_-6a), 2.55 m (1H*,* H-7), 2.38 ddd (1H, *J* = 12.7; 4.0; 2.7 Hz, H_2_-9a), 2.30 s (3H, H_3_-15), 2.22 m (1H, H_2_-6b), 1.85 td (1H, *J* = 12.7; 3.6 Hz, H_2_-9b),1.16 d (3H, *J* = 7.5 Hz, H_3_-14).^13^C NMR (CDCl_3_, 100.03 MHz): 198.6 (C, C-4),169.9 (C, C-12),148.4 (CH, C-2),144.9 (C, C-1),138.2 (C, C-11),137.8 (CH, C-5),124.8 (CH, C-3),118.8 (CH_2_, C-13), 81.4 (CH, C-8), 47.5 (CH, C-7), 36.7 (CH_2_, C-9), 29.2 (CH, C-10), 27.9 (CH_3_, C-15), 27.2 (CH_2_, C-6), 18.9 (CH_3_, C-14). ^1^H NMR identical with literature reported by Yuan et al. [27].

**Isoxanthanol:** ^1^H NMR (CDCl_3_, 400.13 MHz): 6.16 d (1H, *J* = 3.2 Hz, H_2_-13a), 5.75 dd (1H, *J* = 5.6; 3.6 Hz, H-5), 5.43 d (1H, *J* = 3.2 Hz, H_2_-13b), 4.94 m (1H, H-4), 4.29 ddd (1H, *J* = 12.5; 10.4; 3.0 Hz, H-8), 4.12 dd (1H, *J* = 6.9; 6.7 Hz, H-2), 2.80 m (1H*,* H-10), 2.51 m (1H, H_2_-6a), 2.46 m (1H*,* H-7), 2.32 m (1H, H_2_-9a), 2.10 ddd (1H, *J* = 15.1; 11.2; 3.2 Hz, H_2_-6b), 2.03 s (3H, H_3_-17), 1.94 m (1H, H_2_-3a), 1.66 m (1H, H_2_-3b), 1.66 m (1H, H_2_-9b), 1.26 d (3H, *J* = 6.2 Hz, H_3_-15), 1.18 d (3H, *J* = 7.3 Hz, H_3_-14).^13^C NMR (CDCl_3_, 100.03 MHz): 170.7 (C, C-16),170.0 (C, C-12), 149.3 (C, C-1), 139.5 (C, C-11),124.4 (CH, C-5),118.3 (CH_2_, C-13), 82.2 (CH, C-8), 76.8 (CH, C-2), 69.2 (CH, C-4), 47.3 (CH, C-7), 41.9 (CH_2_, C-3), 36.9 (CH_2_, C-9), 29.0 (CH, C-10),25.4 (CH_2_, C-6), 21.4 (CH_3_, C-17), 20.5 (CH_3_, C-15), 19.9 (CH_3_, C-14). ^1^H NMR identical with literature reported by Marco et al. [29].

**8-*epi*-Isoxanthanol:** [*α*]_L_^25^: −16.5 (*c* 0.003, DCM). IR (dry film) 3464, 2964, 2932, 1761, 1734, 1373, 1242 cm^−1^. ^1^H NMR (CDCl_3_, 400.13 MHz): 6.28 d (1H, *J* = 3.2 Hz, H_2_-13a), 5.74 dd (1H, *J* = 8.9; 5.8 Hz, H-5), 5.53 d (1H, *J* = 3.2 Hz, H_2_-13b), 4.96 m (1H, H-4), 4.61 ddd (1H, *J* = 11.4; 8.6; 2.5 Hz, H-8), 4.10 dd (1H, *J* = 7.9; 6.2 Hz, H-2), 3.34 m (1H*,* H-7), 2.60 m (1H*,* H-10), 2.45 m (1H, H_2_-6a), 2.30 m (1H, H_2_-6b), 2.08 m (1H, H_2_-9a), 2.04 s (3H, H_3_-17), 1.97 m (1H, H_2_-3a), 1.83 m (1H, H_2_-9b), 1.71 m (1H, H_2_-3b), 1.29 d (3H, *J* = 6.3 Hz, H_3_-15), 1.20 d (3H, *J* = 6.9 Hz, H_3_-14). ^13^C NMR (CDCl_3_, 100.03 MHz): 170.6 (C, C-16), 170.2 (C, C-12), 147.7 (C, C-1), 138.7 (C, C-11), 122.4 (CH, C-5), 121.8 (CH_2_, C-13), 78.8 (CH, C-8), 73.6 (CH, C-2), 69.2 (CH, C-4), 42.2 (CH_2_, C-3), 41.4 (CH, C-7), 36.9 (CH_2_, C-9), 32.9 (CH, C-10), 26.1 (CH_2_, C-6), 21.9 (CH_3_, C-14), 21.3 (CH_3_, C-17), 20.4 (CH_3_, C-15). HRESIMS m/z [M + Na]^+^ 331.1528 (calcd for C_17_H_24_NaO^+^, 331.1516).

**Ivalbin**: ^1^H NMR (CDCl_3_, 400.13 MHz): 6.15 d (1H, *J* = 3.3 Hz, H_2_-13a), 5.85 dd (1H, *J* = 9.0; 3.5 Hz, H-5), 5.44 d (1H, *J* = 3.3 Hz, H_2_-13b), 4.30 dd (1H, *J* = 6.9; 6.7 Hz, H-2), 4.30 m (1H, H-8), 4.07 m (1H, H-4), 2.85 m (1H*,* H-10), 2.52 ddd (1H, *J* = 15.7; 9.0; 2.5 Hz, H_2_-6a), 2.43 m (1H*,* H-7), 2.32 m (1H, H_2_-9a), 2.11 ddd (1H, *J* = 15.7; 11.8; 4.1 Hz, H_2_-6b), 1.68 m (1H, H_2_-3a), 1.68 m (1H, H_2_-9b), 1.55 m (1H, H_2_-3b), 1.23 d (3H, *J* = 6.2 Hz, H_3_-15), 1.18 d (3H, *J* = 7.3 Hz, H_3_-14).^13^C NMR (CDCl_3_, 100.03 MHz): 170.1 (C, C-12), 150.1 (C, C-1), 139.5 (C, C-11), 123.4 (CH, C-5), 118.3 (CH_2_, C-13), 82.4 (CH, C-8), 79.7 (CH, C-2), 68.8 (CH, C-4), 48.6 (CH, C-7), 43.7 (CH_2_, C-3), 37.0 (CH_2_, C-9), 29.4 (CH, C-10), 25.1 (CH_2_, C-6), 24.4 (CH_3_, C-15),19.6 (CH_3_, C-14). ^1^H NMR identical with literature reported by Marco et al. [29].

**8-*epi*-ivalbin:** [*α*]_D_^25^: + 10.3 (c*c* 0.004, DCM). IR (dry film) 3404, 2958, 2919, 2851, 1739 cm^−1^. ^1^H NMR (CDCl_3_, 400.13 MHz): 6.28 d (1H, *J* = 3.2 Hz, H_2_-13a), 5.82 dd (1H, *J* = 8.8; 5.8 Hz, H-5), 5.53 d (1H, *J* = 3.2 Hz, H_2_-13b), 4.62 ddd (1H, J = 11.4; 8.5; 2.5 Hz, H-8), 4.27 dd (1H, *J* = 10.2; 2.2 Hz, H-2), 4.08 m (1H, H-4), 3.35 m (1H*,* H-7), 2.64 m (1H*,* H-10), 2.47 m (1H, H_2_-6a), 2.32 m (1H, H_2_-6b), 2.08 m (1H, H_2_-9a), 1.84 m (1H, H_2_-9b), 1.71 m (1H, H_2_-3a), 1.59 dt (1H, J = 14.5; 2.5 Hz, H_2_-3b), 1.25 d (3H, *J* = 6.2 Hz, H_3_-15), 1.19 d (3H, *J* = 6.8 Hz, H_3_-14). ^13^C NMR (CDCl_3_, 100.03 MHz): 170.2 (C, C-12), 148.5 (C, C-1), 138.8 (C, C-11), 122.0 (CH, C-5), 121.9 (CH_2_, C-13), 79.2 (CH, C-8), 76.7 (CH, C-2), 69.1 (CH, C-4), 44.6 (CH_2_, C-3), 41.6 (CH, C-7), 37.0 (CH_2_, C-9), 33.4 (CH, C-10), 26.1 (CH_2_, C-6), 24.4 (CH_3_, C-15), 21.9 (CH_3_, C-14). HRESIMS m/z [M + Na]^+^ 289.1416 (calcd for C_15_H_22_NaO_4_^+^, 289.1410).

#### Isolation of 8-epi-xanthatin from *X. cavanillesii*

352 g of *X. cavanillesii* branches conditioned and extracted as described above yielded 44.9 g of dry extract. Partitioning this residue with n-hexane and EtOAc yielded 7.64 g and 15.1 g, respectively. Subsequently, the EtOAc extract was subjected to silica gel column chromatography purification with n-hexane/EtOAc (100:0–0:100) to provide 206 mg of 8-*epi*-xanthatin.

**8-*epi*-xanthatin:** ^1^H NMR (CDCl_3_, 400.13 MHz): 6.98 d (1H, *J* = 16.3 Hz, H-2), 6.32 d (1H, *J* = 3.3 Hz, H_2_-13a), 6.20 dd (1H, *J* = 8.9; 6.3 Hz, H-5), 6.14 d (1H*, J* = 16.3 Hz, H-3), 5.57 d (1H, *J* = 3.3 Hz, H_2_-13b), 4.65 dd (1H, *J* = 12.4; 10.1; 2.6 Hz, H-8), 3.42 m (1H*,* H-7), 2.83 m (1H*,* H-10), 2.59 ddd (1H, *J* = 18.5; 12.3; 6.3 Hz, H_2_-6a), 2.50 m (1H, H_2_-6b), 2.29 s (3H, H_3_-15), 2.18 ddd (1H, *J* = 12.4; 8.8; 2.2 Hz, H_2_-9a), 1.91 ddd (1H, *J* = 16.2; 12.7; 3.6 Hz, H_2_-9b), 1.18 d (3H, *J* = 6.9 Hz, H_3_-14).^13^C NMR (CDCl_3_, 100.03 MHz): 198.6 (C, C-4), 169.8 (C, C-12), 146.4 (CH, C-2), 142.8 (C, C-1), 138.2 (C, C-11),135.6 (CH, C-5), 125.9 (CH, C-3), 122.4 (CH_2_, C-13), 78.2 (CH, C-8), 41.2 (CH, C-7), 36.3 (CH_2_, C-9), 31.8 (CH, C-10), 27.7 (CH_3_, C-15), 27.0 (CH_2_, C-6), 21.5 (CH_3_, C-14). ^1^H NMR identical with literature reported by Kummer et al*.* [28].

### Derivatization of *X. cavanillesii* EtOAc extracts

#### Derivatization with ammonium hydroxide

94 mg of the EtOAc extract of *X. cavanillesii* were dissolved in 3 mL of ammonium hydroxide and stirred for 4 h at room temperature. Afterwards, the reaction crude was extracted with DCM (3 × 10 mL). The combined organic extracts were dried over anhydrous MgSO_4_ and evaporated, and the resulting residue was purified by preparative TLC (5% of MeOH and 1% of triethylamine in DCM) to yield 2.7 mg of compound **1** (7%).

*(3S,3aR,7S,8aR)-3-(Aminomethyl)-7-methyl-6-((E)-3-oxobut-1-en-1-yl)-3,3a,4,7,8,8a-hexahydro-2H-cyclohepta[b]furan-2-one* (**1**). Yellow oil; [*α*]_D_^25^: + 21.6 (*c* 0.002, DCM). IR (dry film) 2958, 2926, 1762, 1666, 1363, 1263 cm^−1^. ^1^H NMR (CDCl_3_, 400.13 MHz): 6.97 d (1H, *J* = 16.0 Hz, H-2), 6.14 m (1H, H-5), 6.12 d (1H*, J* = 16.0 Hz, H-3), 4.48 ddd (1H, *J* = 12.2; 8.5; 2.0 Hz, H-8), 2.98 dd (1H, *J* = 12.4; 4.7 Hz, H_2_-13a), 2.90 dd (1H, *J* = 12.4; 5.2 Hz, H_2_-13b), 2.82 m (1H*,* H-10), 2.79 m (1H*,* H-7), 2.45 m (1H*,* H-11), 2.44 m (2H, H_2_-6), 2.28 s (3H, H_3_-15), 2.13 m (1H, H_2_-9a), 1.96 m (1H, H_2_-9b), 1.19 d (3H, *J* = 7.0 Hz, H_3_-14). ^13^C NMR (CDCl_3_, 100.03 MHz): 198.5 (C, C-4),177.6 (C, C-12),146.4 (CH, C-2),142.5 (C, C-1), 135.9 (CH, C-5), 125.7 (CH, C-3), 78.6 (CH, C-8), 48.0 (CH_2_, C-13), 44.9 (CH, C-11), 39.8 (CH, C-7), 35.2 (CH_2_, C-9), 31.5 (CH, C-10), 27.5 (CH_3_, C-15), 27.4 (CH_2_, C-6), 21.0 (CH_3_, C-14). HRESIMS m/z [M + H]^+^ 264.1637 (calcd for C_15_H_22_NO_3_^+^, 264.1594).

#### Derivatization with trimethylsilyl azide

To a round bottom flask were added 196 μL of trimethylsilyl azide, 3 mL of DCM and 84 μL of AcOH. Afterwards 73 mg of the EtOAc extract of *X. cavanillesii* and 9 μL of TEA were added and stirred for 48 h at room temperature. The reaction was quenched by pouring the mixture into ice water; subsequently, the aqueous solution was extracted with AcOEt (3 × 30 mL). Organic layers were combined and washed with saturated NaHCO_3_ solution and brine, and dried with Na_2_SO_4_, filtered, and evaporated. Preparative TLC purification of the crude with a mixture of EtOAc and *n*-hex (1:1) resulting in 9.7 mg of compound **2** (24%).

*(3S,3aR,7S,8aR)-3-(Azidomethyl)-7-methyl-6-((E)-3-oxobut-1-en-1-yl)-3,3a,4,7,8,8a-hexahydro-2H-cyclohepta[b]furan-2-one* (**2**)*.* Yellow oil. [*α*]_D_^25^: + 31.2 (*c* 0.006, DCM). IR (dry film) 2963, 2930, 2107, 1770, 1716, 1452, 1365, 1277, 1184 cm^−1^. ^1^H NMR (CDCl_3_, 400.13 MHz): 6.97 d (1H, *J* = 16.1 Hz, H-2), 6.14 m (1H, H-5), 6.13 d (1H*, J* = 16.1 Hz, H-3), 4.53 ddd (1H, *J* = 12.4; 8.7; 1.9 Hz, H-8), 3.68 brd (2H, *J* = 4.9 Hz, H_2_-13), 2.85 m (1H*,* H-10), 2.84 m (1H*,* H-7), 2.51 m (1H*,* H-11), 2.50 m (2H, H_2_-6), 2.30 s (3H, H_3_-15), 2.16 m (1H, H_2_-9a), 1.95 m (1H, H_2_-9b), 1.20 d (3H, *J* = 7.0 Hz, H_3_-14).^13^C NMR (CDCl_3_, 100.03 MHz): 198.6 (C, C-4), 175.3 (C, C-12), 146.1 (CH, C-2), 142.9 (C, C-1),135.3 (CH, C-5), 125.9 (CH, C-3), 78.5 (CH, C-8), 49.6 (CH_2_, C-13), 44.6 (CH, C-11), 40.0 (CH, C-7), 35.1 (CH_2_, C-9), 31.6 (CH, C-10), 27.6 (CH_3_, C-15), 27.3 (CH_2_, C-6), 21.2 (CH_3_, C-14). HRESIMS m/z [M + HN_2_]^+^ 262.1446 (calcd for C_15_H_20_NO_3_^+^, 262.1438).

#### Derivatization with hydroxylammonium chloride

To a solution of EtOAc extract (130 mg) in MeOH (3 mL) were added 38 mg of [NH₄OH]Cl and the reaction mixture stirred for 24 h at room temperature. After, 5 mL of H_2_O was added, and the pH of the reaction was adjusted to 7 with a saturated NaHCO_3_ solution. MeOH was evaporated and the aqueous phase was extracted with DCM (3 × 30 mL). Organic layers were combined and dried with Na_2_SO_4_, filtered, and evaporated. Preparative TLC purification was carried out with a mixture of EtOAc and *n*-hex (1:1), resulting in 17.0 mg of product **3** (26%) and 20.6 mg of product **3’** (32%).

*(3aR,7S,8aR)-6-((1E,3E)-3-(Hydroxyimino)but-1-en-1-yl)-7-methyl-3-methylene-3,3a,4,7,8,8a-hexahydro-2H-cyclohepta[b]furan-2-one* (**3**)*.* Grayish oil; [*α*]_D_^25^: + 28.1 (*c* 0.004, DCM). IR (dry film) 3335, 2960, 2932, 2873, 1765, 1272 cm^−1^. ^1^H NMR (CDCl_3_, 400.13 MHz): 6.36 d (1H*, J* = 16.5 Hz, H-2), 6.30 d (1H, *J* = 3.4 Hz, H_2_-13a), 6.22 d (1H, *J* = 16.5 Hz, H-3), 6.92 dd (1H, *J* = 9.0; 6.3 Hz, H-5), 5.55 d (1H, *J* = 3.0 Hz, H_2_-13b), 4.65 ddd (1H, *J* = 12.2; 8.8; 2.2 Hz, H-8), 3.39 m (1H*,* H-7), 2.86 m (1H*,* H-10), 2.55 ddd (1H, *J* = 19.8; 12.8; 6.2 Hz, H_2_-6a), 2.42 m (1H, H_2_-6b), 2.14 m (1H, H_2_-9a), 2.02 s (3H, H_3_-15), 1.88 m (1H, H_2_-9b), 1.18 d (3H, *J* = 6.9 Hz, H_3_-14).^13^C NMR (CDCl_3_, 100.03 MHz): 169.9 (C, C-12), 156.8 (C, C-4), 143.6 (C, C-1), 138.5 (C, C-11), 136.3 (CH, C-2), 128.8 (CH, C-5), 124.5 (CH, C-3), 121.6 (CH_2_, C-13), 78.4 (CH, C-8), 41.6 (CH, C-7), 36.6 (CH_2_, C-9), 31.8 (CH, C-10), 26.9 (CH_2_, C-6), 21.6 (CH_3_, C-14), 9.55 (CH_3_, C-15). HRESIMS m/z [M + Na]^+^ 284.1248 (calcd for C_15_H_19_NNaO_3_^+^, 284.1257).

*(3aR,7S,8aR)-6-((1E,3Z)-3-(Hydroxyimino)but-1-en-1-yl)-7-methyl-3-methylene-3,3a,4,7,8,8a-hexahydro-2H-cyclohepta[b]furan-2-one* (**3’**)*.* Greenish oil; [*α*]_D_^25^: + 50.0 (*c* 0.005, DCM). IR (dry film) 3285, 2960, 2933, 1762, 1616, 1373, 1274 cm^−1^. ^1^H NMR (CDCl_3_, 400.13 MHz): 6.96 d (1H*, J* = 16.6 Hz, H-3), 6.41 d (1H, *J* = 16.6 Hz, H-2), 6.30 d (1H, *J* = 3.2 Hz, H_2_-13a), 6.00 dd (1H, *J* = 8.9; 6.3 Hz, H-5), 5.55 d (1H, *J* = 3.2 Hz, H_2_-13b), 4.65 dd (1H, *J* = 14.4; 9.0; 5.0 Hz, H-8), 3.39 m (1H*,* H-7), 2.93 m (1H*,* H-10), 2.57 ddd (1H, *J* = 19.8; 12.8; 6.2 Hz, H_2_-6a), 2.44 m (1H, H_2_-6b), 2.16 m (1H, H_2_-9a), 2.02 s (3H, H_3_-15), 1.89 m (1H, H_2_-9b),1.21 d (3H, *J* = 6.9 Hz, H_3_-14).^13^C NMR (CDCl_3_, 100.03 MHz): 169.9 (C, C-12),153.4 (C, C-4),143.8 (C, C-1),139.3 (CH, C-2),138.5 (C, C-11),130.9 (CH, C-5),122.1 (CH_2_, C-13), 115.4 (CH, C-3),78.4 (CH, C-8), 41.4 (CH, C-7), 36.6 (CH_2_, C-9), 31.8 (CH, C-10), 26.9 (CH_2_, C-6), 21.6 (CH_3_, C-14),16.8 (CH_3_, C-15). HRESIMS m/z [M + H]^+^ 262.1439 (calcd for C_15_H_20_NO_3_^+^, 262.1438).

#### Derivatization with propargylamine

To 213 mg of EtOAc extract in 4 mL of MeOH was added 86 μL propargylamine, and the reaction mixture stirred for 28 h at room temperature. The solvent was evaporated and the residue purified by column chromatography with *n*-hexane/EtOAc mixtures of increasing polarity (100:0–0:100) to yield 68.2 mg (56%) of compound **4**.

*(3S,3aR,7S,8aR)-7-Methyl-6-((E)-3-oxobut-1-en-1-yl)-3-((prop-2-yn-1-ylamino)methyl)-3,3a,4,7,8,8a-hexahydro-2H-cyclohepta[b]furan-2-one* (**4**). Yellow oil; [*α*]_D_^25^: + 7.4 (*c* 0.003, DCM). IR (dry film) 32,980, 2957, 2928, 1762, 1665, 1595, 1361, 1258, 1179, 1019, 984 cm^−1^. ^1^H NMR (CDCl_3_, 400.13 MHz): 6.97 d (1H, *J* = 16.2 Hz, H-2), 6.13 dd (1H,* J* = 8.9; 6.6 Hz, H-5), 6.12 d (1H*, J* = 16.2 Hz, H-3), 4.49 ddd (1H, *J* = 12.4; 8.6; 1.9 Hz, H-8), 3.44 t (2H, *J* = 2.5 Hz, H_2_-1´), 2.94 ddd (2H, *J* = 22.2; 12.1; 6.4 Hz, H_2_-13), 2.82 m (1H*,* H-10), 2.80 m (1H*,* H-7), 2.50 m (1H*,* H-11), 2.47 m (2H, H_2_-6), 2.28 s (3H, H_3_-15), 2.22 m (1H*,* H-2´), 2.14 m (1H, H_2_-9a), 1.96 m (1H, H_2_-9b), 1.19 d (3H, *J* = 6.9 Hz, H_3_-14).^13^C NMR (CDCl_3_, 100.03 MHz): 198.6 (C, C-4), 177.5 (C, C-12), 146.4 (CH, C-2), 142.6 (C, C-1), 135.8 (CH, C-5),125.6 (CH, C-3), 81.5 (C, C-1´), 78.5 (CH, C-8), 72.0 (CH, C-2´), 47.2 (CH_2_, C-13), 44.7 (CH, C-11), 40.2 (CH, C-7), 38.4 (CH_2_, C-16), 35.1 (CH_2_, C-9), 31.6 (CH, C-10), 27.6 (CH_3_, C-15), 27.4 (CH_2_, C-6), 21.1 (CH_3_, C-14). HRESIMS m/z [M + H]^+^ 302.1753 (calcd for C_18_H_24_NO_3_^+^, 302.1751).

#### Extract derivatization with potassium hydroxide

To a round bottom flask was added 2 ml of DCM and 68.5 mg of KOH and stirred for 30 min at room temperature. Afterwards, 150 mg of the EtOAc extract was dissolved in 2 mL of DCM was added and stirred for another 2 h. The solvent was evaporated, 5 ml of water was added, and pH neutralized with a 4 M HCl solution. Then, the aqueous phase was extracted with EtOAc (3 × 30 mL). The combined organic extracts were dried over anhydrous MgSO_4_ and evaporated, and the resulting residue was purified by preparative TLC (EtOAc/*n*-hexane, 3:2) to yield 24.9 mg of compound **6** (31%).

*(3S,3aR,7S,8aR)-3-(Methoxymethyl)-7-methyl-6-((E)-3-oxobut-1-en-1-yl)-3,3a,4,7,8,8a-hexahydro-2H-cyclohepta[b]furan-2-one* (**6**)*.* Yellow oil; [*α*]_D_^25^: + 27.6 (*c* 0.013, DCM). IR (dry film) 2928, 2877, 1770, 1671, 1363, 1189, 1118 cm^−1^. ^1^H NMR (CDCl_3_, 400.13 MHz): 6.97 d (1H, *J* = 16.3 Hz, H-2), 6.14 m (1H, H-5), 6.12 d (1H*, J* = 16.3 Hz, H-3), 4.49 ddd (1H, *J* = 12.4; 8.8; 2.2 Hz, H-8), 3.65 d (2H, *J* = 4.6 Hz, H_2_-13), 3.36 s (3H, H_3_-1´), 2.91 m (1H*,* H-7), 2.82 m (1H*,* H-10), 2.49 m (1H*,* H-11), 2.48 m (2H, H_2_-6), 2.28 s (3H, H_3_-15), 2.13 ddd (1H, *J* = 13.8; 7.5; 2.2 Hz, H_2_-9a), 1.96 m (1H, H_2_-9b), 1.18 d (3H, *J* = 7.0 Hz, H_3_-14).^13^C NMR (CDCl_3_, 100.03 MHz): 198.6 (C, C-4), 176.2 (C, C-12), 146.6 (CH, C-2),142.5 (C, C-1),136.2 (CH, C-5),125.6 (CH, C-3), 78.9 (CH, C-8), 70.2 (CH_2_, C-13), 59.1 (CH_3_, C-1´), 45.5 (CH, C-11), 39.8 (CH, C-7), 35.1 (CH_2_, C-9), 31.5 (CH, C-10), 27.6 (CH_3_, C-15), 27.5 (CH_2_, C-6), 21.3 (CH_3_, C-14). HRESIMS m/z [M + Na]^+^ 301.1419 (calcd for C_16_H_22_NaO_4_^+^, 301.1410).

#### Derivatization with *m*-chloroperoxybenzoic acid

To a solution of EtOAc extract (146 mg) in DCM (3 mL) at 0 °C was added 153 mg of MCPBA, and the reaction mixture was stirred for 24 h at room temperature. Subsequently, the solvent was evaporated, and the residue redissolved in EtOAc, washed with an aqueous Na_2_S_2_O_3_ solution, then with NaHCO_3_ and finally with H_2_O. The organic layer was dried with Na_2_SO_4_, filtered, and evaporated. Preparative TLC purification (EtOAc/*n*-hex, 1:1), resulting in 26.1 mg of **7** (31%) and 14.6 mg of compound **8** (13%).

*(1aS,2S,3aR,6aR,7aS)-2-Methyl-6-methylene-1a-((E)-3-oxobut-1-en-1-yl)octahydro-5H-oxireno[2',3':4,5]cyclohepta[1,2-b]furan-5-one* (**7**)*.* Colorless oil; [*α*]_D_^25^: + 6.1 (*c* 0.018, DCM). IR (dry film) 2967, 2932, 1763, 1673, 1627, 1273, 968 cm^−1^. ^1^H NMR (CDCl_3_, 400.13 MHz): 6.74 d (1H, *J* = 15.8 Hz, H-2), 6.29 m (1H, H_2_-13a), 6.28 d (1H*, J* = 15.8 Hz, H-3), 5.66 d (1H, *J* = 1.9 Hz, H_2_-13b), 4.61 dd (1H, *J* = 11.7; 7.4; 4.2 Hz, H-8), 3.30 m (1H*,* H-7), 3.07 dd (1H, *J* = 7.7; 4.9 Hz, H-5), 2.29 m (1H*,* H-10), 2.25 s (3H, H_3_-15), 2.15 ddd (1H, *J* = 15.1; 7.8; 3.4 Hz, H_2_-6a), 2.05 m (1H, H_2_-6b),1.86 ddd (1H, *J* = 14.1; 3.9; 2.6 Hz, H_2_-9a), 1.71 m (1H, H_2_-9b),1.13 d (3H, *J* = 6.9 Hz, H_3_-14).^13^C NMR (CDCl_3_, 100.03 MHz): 197.7 (C, C-4),169.0 (C, C-12),146.7 (CH, C-2),139.0 (C, C-11),129.6 (CH, C-3),123.2 (CH_2_, C-13),79.4 (CH, C-8), 65.8 (CH, C-5), 62.7 (C, C-1), 39.3 (CH, C-7), 32.2 (CH_2_, C-9), 31.6 (CH, C-10), 31.2 (CH_2_, C-6), 28.3 (CH_3_, C-15), 18.8 (CH_3_, C-14). HRESIMS m/z [M + Na]^+^ 285.1090 (calcd for C_15_H_18_NaO_4_^+^, 285.1097).

*(2Z,4Z)-4-((3aR,5R,7S,8aR)-5-Hydroxy-7-methyl-3-methylene-2-oxooctahydro-6H-cyclohepta[b]furan-6-ylidene)but-2-en-2-yl 3-chlorobenzoate* (**8**)*.* Colorless oil; [*α*]_D_^25^: + 44.7 (*c* 0.009, DCM). IR (dry film) 3491, 2961, 2928, 1768, 1663, 1576, 1426, 1376, 1280, 1263, 1223, 1125, 1004 cm^−1^. ^1^H NMR (CDCl_3_, 400.13 MHz): 8.01 t (1H*, J* = 1.6 Hz, H-3´), 7.93 dt (1H*, J* = 7.8; 1.6 Hz, H-7´), 7.67 d (1H*, J* = 9.0 Hz, H-3),7.59 ddd (1H*, J* = 8.0; 2.0; 1.0 Hz, H-5´), 7.42 t (1H*, J* = 7.8 Hz, H-6´), 6.32 d (1H, *J* = 3.2 Hz, H_2_-13a), 5.63 d (1H, *J* = 2.7 Hz, H_2_-13b), 5.53 d (1H, *J* = 9.0 Hz, H-2), 5.20 t (1H, *J* = 7.9 Hz, H-5), 4.58 ddd (1H, *J* = 12.5; 8.3; 3.2 Hz, H-8), 3.14 m (1H*,* H-7),2.63 dd (1H*,* J = 16.1; 8.0 H-10),2.37 ddd (1H, *J* = 14.0; 6.8; 3.6 Hz, H_2_-6a), 2.22 m (1H, H_2_-9a), 2.18 s (3H, H_3_-15), 2.11 m (1H, H_2_-6b), 1.81 m (1H, H_2_-9b),1.33 d (3H, *J* = 7.3 Hz, H_3_-14).^13^C NMR (CDCl_3_, 100.03 MHz): 169.7 (C, C-12), 168.9 (C, C-4), 164.2 (C, C-1´),152.3 (C, C-1), 138.3 (C, C-11), 135.0 (C, C-2´), 134.9 (C, C-4´), 134.1 (CH, C-5´), 130.0 (CH, C-3´), 129.9 (CH, C-6´), 128.0 (CH, C-7´), 123.7 (CH, C-2), 122.8 (CH_2_, C-13), 88.7 (CH, C-3), 78.1 (CH, C-8), 68.4 (CH, C-5), 39.1 (CH, C-10), 37.5 (CH, C-7), 35.9 (CH_2_, C-9), 34.7 (CH_2_, C-6), 23.3 (CH_3_, C-14), 20.8 (CH_3_, C-15). HRESIMS m/z [M + Na + OMe]^+^ 457.1070 (calcd for C_23_H_27_ClO_6_Na^+^, 457.1388).

#### Click reaction of product 4 with benzyl azide.

To a solution of **4** (21 mg, 0.087 mmol) in DMF (3 mL) were added 22 μL of benzyl azide (0.174 equiv), 7 mg of NaAsc (0.035 equiv) and 5 mg of CuSO_4_ (0.017 equiv). The reaction mixture was stirred for 24 h at room temperature. The work up involved addition of distilled water and extraction with DCM (3 × 30 mL). Preparative TLC purification of the crude with EtOAc resulting in 7.0 mg of product **5** (21%).

*(3S,3aR,7S,8aR)-3-((((1-Benzyl-1H-1,2,3-triazol-4-yl)methyl)amino)methyl)-7-methyl-6-((E)-3-oxobut-1-en-1-yl)-3,3a,4,7,8,8a-hexahydro-2H-cyclohepta[b]furan-2-on* (**5**)*.* Yellow oil; [*α*]_D_^25^: + 11.0 (*c* 0.003, DCM). IR (dry film) 2956, 2932, 1766, 1665, 1622, 1595, 1456, 1360, 1258, 1176, 726 cm^−1^. ^1^H NMR (CDCl_3_, 400.13 MHz): 7.47 s (1H*,* H-5´), 7.37 m (1H*,* H-8´), 7.35 m (2H*,* H-7´), 7.27 m (2H*,* H-6´), 6.95 d (1H, *J* = 16.2 Hz, H-2), 6.11 d (1H*, J* = 16.2 Hz, H-3), 6.08 m (1H, *J* = 8.9; 6.3 Hz, H-5), 5.50 s (2H, H_2_-4´), 4.46 m (1H, H-8), 3.95 s (2H, H_2_-1´), 2.98 dd (1H, *J* = 12.3; 5.0 Hz, H_2_-13a), 2.91 dd (1H, *J* = 12.3; 6.5 Hz, H_2_-13b), 2.81 m (1H*,* H-10), 2.73 m (1H*,* H-7), 2.56 m (1H*,* H-11), 2.49 m (1H, H_2_-6a), 2.39 m (1H, *J* = 14.0; 9.0; 5.1 Hz, H_2_-6b), 2.28 s (3H, H_3_-15), 2.12 m (1H, H_2_-9a),1.95 m (1H, H_2_-9b),1.17 d (3H, *J* = 7.0 Hz, H_3_-14).^13^C NMR (CDCl_3_, 100.03 MHz): 198.7 (C, C-4),177.8 (C, C-12),146.4 (CH, C-2),145.7 (C, C-2´),142.5 (C, C-1),136.0 (CH, C-5),129.1 (CH, C-8´),128.9 (CH, C-7´),128.1 (CH, C-6´),125.7 (CH, C-3),121.8 (CH, C-3´), 78.9 (CH, C-8), 54.6 (CH_2_, C-4´), 47.6 (CH_2_, C-13), 44.8 (CH_2_, C-1´), 44.3 (CH, C-11), 40.3 (CH, C-7), 35.0 (CH_2_, C-9),31.6 (CH, C-10), 27.7 (CH_3_, C-15), 27.3 (CH_2_, C-6), 21.1 (CH_3_, C-14). HRESIMS m/z [M + H]^+^ 435.2405 (calcd for C_25_H_31_N_4_O_3_^+^, 435.2391).

### Isoxanthanol esterification’s with carboxylic acids

#### Derivatization with cinnamic acid

To a round-bottom flask, 24 mg (0.078 mmol) of isoxanthanol was added and dissolved in DCM (3 mL). Separately, 15 mg (0.102 mmol) of cinnamic acid was dissolved in DCM (1 mL) and added stepwise to the previous solution at 0°C with continuous stirring. Subsequently, 21 mg (0.102 mmol) of DCC and 2 mg (0.011 mmol) of DMAP, both dissolved in DCM (1 mL), were added to the reaction mixture at 0°C. The mixture was stirred at room temperature overnight. Afterwards, EtOAc and a concentrated solution of NH₄Cl was added, followed by filtration through Celite. Then, the organic layer was washed with a concentrated solution of NH₄Cl and brine. Organic layer was dried with Na₂SO₄, filtered and evaporated. Preparative TLC purification of the crude with (EtOAc/*n*-hex, 1:1) resulting in 17.2 mg of product **9** (50%).

*(1S,3S)-3-acetoxy-1-((3aS,7S,8aS)-7-methyl-3-methylene-2-oxo-3,3a,4,7,8,8a-hexahydro-2H-cyclohepta[b]furan-6-yl)butyl cinnamate* (**9**)**.** Yellow oil; [*α*]_D_^25^: + 12.9 (*c* 0.007, DCM). IR (dry film) 2974, 2934, 2863, 1771, 1732, 1715, 1637, 1248 cm^−1^. ^1^H NMR (CDCl_3_, 400.13 MHz): 7.69 d (1H, *J* = 16.0 Hz, H-20), 7.53 m (2H, H-22,22´), 7.39 m (3H*,* H-23,23´,24), 6.42 d (1H,* J* = 16.0 Hz, H-19), 6.16 d (1H, *J* = 3.2 Hz, H_2_-13a), 5.93 dd (1H, *J* = 9.1; 3.4 Hz, H-5), 5.44 d (1H, *J* = 3.0 Hz, H-13b), 5.34 t (1H, *J* = 7.3 Hz, H-2), 4.90 m (1H, H-4), 4.29 td (1H, *J* = 11.3;2.8 Hz, H-8), 2.86 m (1H*,* H-10), 2.51 m (H, H-6a), 2.48 m (H, H-7), 2.34 ddd (1H, *J* = 12.7; 4.4; 3.0 Hz, H-9a), 2.18 m (H, H-3a), 2.15 m (H, H-6b), 2.07 s (3H, H_3_-17), 1.80 ddd (1H, *J* = 14.1; 7.4; 5.0 Hz, H-3b), 1.72 td (H, J = 12.3;3.5 Hz, H-9b), 1.26 d (3H, *J* = 6.2 Hz, H_3_-15), 1.11 d (3H, *J* = 7.3 Hz, H_3_-14). ^13^C NMR (CDCl_3_, 100.03 MHz): 170.5 (C, C-16), 170.0 (C, C-12), 166.1 (C, C-18), 145.4 (CH, C-20), 144.8 (C, C-1), 139.3 (C, C-11), 134.3 (C, C-21), 130.5 (CH, C-24´), 128.9 (2CH, C-23, 23´), 128.2 (2CH, C-22, 22´), 127.5 (CH, C-5), 118.5 (CH_2_, C-13), 117.9 (CH, C-19), 82.0 (CH, C-8), 78.0 (CH, C-2), 67.9 (CH, C-4), 48.0 (CH, C-7), 39.2 (CH_2_, C-3), 37.0 (CH_2_, C-9), 29.3 (CH, C-10), 25.5 (CH_2_, C-6), 21.3 (3C, CH_3_-17), 20.2 (3H, CH_3_-15), 19.4 (3H, CH_3_-14). HRESIMS m/z [M + Na]^+^ 461.1944 (calcd for C_26_H_30_NaO_6_^+^, 461.1935).

#### Derivatization X 4-oxo-4-(*p*-tolyl)butanoic acid

To a round-bottom flask, 28 mg (0.091 mmol) of isoxanthanol was added and dissolved in 3 mL of DCM. Separately, 23 mg (0.118 mmol) of 4-oxo-4-(*p*-tolyl)butanoic acid was dissolved in 1 mL of DCM and added stepwise to the previous solution at 0°C with continuous stirring. Subsequently, 24 mg (0.118 mmol) of DCC and 2 mg (0.014 mmol) of DMAP, both dissolved in 1 mL of DCM, were added to the reaction mixture at 0°C. The mixture was stirred at room temperature overnight. The reaction mixture was then quenched by adding EtOAc and a concentrated solution of NH₄Cl, followed by filtration through Celite. The organic layer was washed with a concentrated NH₄Cl solution and brine. After drying the organic layer over Na₂SO₄, it was filtered and evaporated. Preparative TLC purification of the crude with (EtOAc/*n*-hex, 1:1) resulting in 19.7 mg of product **10** (49%).

*(1S,3S)-3-acetoxy-1-((3aS,7S,8aS)-7-methyl-3-methylene-2-oxo-3,3a,4,7,8,8a-hexahydro-2H-cyclohepta[b]furan-6-yl)butyl 4-oxo-4-(p-tolyl)butanoate* (**10**). Yellow oil; [*α*]_D_^25^:−33.6 (*c* 0.006, DCM). IR (dry film) 2975, 2932, 2860, 2360, 1770, 1736, 1686, 1249 cm^−1^. ^1^H NMR (CDCl_3_, 400.13 MHz): 7.86 d (2H, *J* = 8.2 Hz, H-24,24´), 7.25 d (2H, *J* = 8.2 Hz, H-23,23´), 6.15 d (1H, *J* = 3.2 Hz, H-13a), 5.85 dd (1H, *J* = 9.1; 3.4 Hz, H-5), 5.43 d (1H, *J* = 3.0 Hz, H-13b), 5.23 t (1H, *J* = 7.2 Hz, H-2), 4.85 m (1H, H-4), 4.29 td (1H, *J* = 11.2;2.8 Hz, H-8), 3.27 t (2H, J = 6.2 Hz, H_2_-20), 2.78 m (1H, H-10), 2.74 m (2H, H_2_-19), 2.50 m (1H, H-6a), 2.47 m (1H, H-7), 2.41 s (3H, H_3_-25), 2.31 ddd (1H, *J* = 12.7, 4.4, 3.1, H-9a), 2.24 m (1H, H-3a), 2.09 m (1H, H-6b), 2.04 m (1H, H-3b), 2.03 s (3H, H_3_-17), 1.68 m td (1H, J = 12.8;3.5 Hz, H-9b), 1.24 d (3H, *J* = 6.3 Hz, H_3_-15), 1.13 d (3H, *J* = 7.3 Hz, H_3_-14). ^13^C NMR (CDCl_3_, 100.03 MHz): 197.4 (C, C-21), 172.0 (C, C-18), 170.4 (C, C-16), 169.8 (C, C-12), 144.5 (C, C-1), 144.1 (C, C-25), 139.0 (C, C-11), 128.1 (C, C-24), 129.3 (CH, C-23), 127.5 (CH, C-5), 118.3 (CH_2_, C-13), 82.1 (CH, C-8), 78.1 (CH, C-2), 67.8 (CH, C-4), 47.9 (CH, C-7), 39.1 (CH_2_, C-3), 37.0 (CH_2_, C-9), 33.1 (CH2, C-20), 29.5 (CH, C-10), 28.3 (CH_2_, C-19), 25.3 (CH_2_, C-6), 21.6 (CH_3_, C-26), 21.2 (CH_3_, C-17), 20.1 (CH_3_, C-15), 19.3 (CH_3_, C-14). HRESIMS m/z [M + Na]^+^ 505.2205 (calcd for C_28_H_34_NaO_7_^+^, 505.2197).

### Biological assays

#### Bacterial strains

*Staphylococcus aureus* (ATCC 25923), methicillin resistant *S. aureus* (ATCC 43300), *Escherichia coli* (ATCC 25922), *Candida albicans* ATCC 10231, *C. tropicalis* ATCC 66029, and *Cryptococcus. neoformans* ATCC 66031 were purchased from Bioartis SRL (Buenos Aires, Argentina). Bacterial strains were stored at − 80 °C in tryptic soy (TS) broth (Oxoid Ltd., Basingstoke, Hampshire, U.K.) with added 20% glycerol. Strains were revived by plating TS agar and incubated at 36 °C 24–48 h. In the case of yeast species, the same procedure was followed using Sabouraud glucose (SG) broth.

#### Determination of minimum inhibitory concentration (MIC)

The MIC of the extracts, fractions and isolated compounds were determined in multiwell plates by the standard broth microdilution method described by the Clinical and Laboratory Standards Institute (CLSI) [42]. Briefly, 1:2 serial dilutions of each sample to be evaluated were prepared in 100 µL of Mueller Hinton (MHB) broth (Oxoid Ltd., Basingstoke, Hampshire, U.K.), ranging from 1 to 0.008 mg/mL. 100 μL of a standardized bacterial inoculum (10^5^ CFU/mL) were added to each well. The MIC values were recorded as the lowest concentration of the compound at which no signs of growth were observed, based on the OD_625_ value of less than 0.05 after 24 h of incubation. Meanwhile, antibiotics ampicillin was included as control using the following concentration window: 0.1 to 0.5 × 10^–4^ mg/mL. For the MIC evaluation of yeast-like species, SG broth was used. The multiwell plates were incubated for 48–72 h, and growth determination was based on OD_530_ nm values, with ketoconazole included as a positive control (1.5 × 10^−2 ^to 1.2 × 10^–4^ mg/mL). In all cases, negative and microbial growth controls were performed with 1% DMSO.

#### Minimum bactericide (MBC) and fungicidal (MFC) concentration

Briefly, from the MIC determination, a known aliquot was removed from the wells corresponding to MIC, 2xMIC and 4xMIC and seeded in TS agar plates. The lowest concentration of compounds that provided no bacterial growth after 24 h of incubation at 36 °C was the MBC. For the determination MFC, subculture was carried out on SG agar plates.

#### Modulation of the antimicrobial activity assay

For the evaluation of pure compounds as antibiotic and antifungal modulators, the MICs of ampicillin, linezolid, ketoconazole, and amphotericin B were determined in the presence or absence of 8-*epi*-xanthine at a subinhibitory concentration (1/2 MIC). The concentrations of the antibiotic or antifungal agent tested were consistent with the MIC determination assay. In the wells containing a combination of antimicrobial drugs and 8-*epi*-xanthine, a fixed amount of the latter was added to ensure that the final concentration was consistent with its MIC/2. The microplates were incubated at 37°C for 24–48 h, and growth was determined by reading the OD_625_ or OD_350_ nm for *S. aureus* and *C. albicans*, respectively. The analyzed metabolite was considered a “potentiator” of antimicrobial activity when the combined MIC was lower than the MIC of the antimicrobial drug and “non-potentiator” when no changes were observed (combined MIC ≥ antimicrobial MIC). Minor variations in MIC were not considered significant [43].

## Supplementary Information

Additional file 1.

## Data Availability

All data generated or analysed during this study are included in this published article [and its supplementary information files]. If something has been omitted, the datasets are available from the corresponding author on reasonable request.

## References

[1] Global antimicrobial resistance and use surveillance system (GLASS) report 2022. Geneva: World Health Organization; 2022. Licence: CC BY-NC-SA 3.0 IGO.

[2] Lancet T. Antimicrobial resistance: an agenda for all. The Lancet. 2024;403:2349. 10.1016/S0140-6736(24)01076-6. 10.1016/S0140-6736(24)01076-6 38797177

[3] O’Neill J. Antimicrobial Resistance: Tackling a crisis for the health and wealth of nations, The Review on Antimicrobial Resistance (2014) 1–17.

[4] Chinemerem Nwobodo D, Ugwu MC, Oliseloke Anie C, Al-Ouqaili MTS, Chinedu Ikem J, Victor Chigozie U, Saki M. Antibiotic resistance: the challenges and some emerging strategies for tackling a global menace. J Clin Lab Anal. 2022;36:e24655–e24655. 10.1002/jcla.24655. 35949048 10.1002/jcla.24655 PMC9459344

[5] Rice LB. Progress and challenges in implementing the research on ESKAPE pathogens. Infect Control Hosp Epidemiol. 2010;31:S7–10. 10.1086/655995. 20929376 10.1086/655995

[6] Robbins N, Caplan T, Cowen LE. Molecular evolution of antifungal drug resistance. Annu Rev Microbiol. 2017;71:753–75. 10.1146/annurev-micro-030117-020345. 28886681 10.1146/annurev-micro-030117-020345

[7] Bhattacharya S, Sae-Tia S, Fries BC. Candidiasis and mechanisms of antifungal resistance. Antibiotics. 2020. 10.3390/antibiotics9060312. 32526921 10.3390/antibiotics9060312 PMC7345657

[8] Fisher MC, Alastruey-Izquierdo A, Berman J, Bicanic T, Bignell EM, Bowyer P, Bromley M, Brüggemann R, Garber G, Cornely OA, Gurr SJ, Harrison TS, Kuijper E, Rhodes J, Sheppard DC, Warris A, White PL, Xu J, Zwaan B, Verweij PE. Tackling the emerging threat of antifungal resistance to human health. Nat Rev Microbiol. 2022;20:557–71. 10.1038/s41579-022-00720-1. 35352028 10.1038/s41579-022-00720-1 PMC8962932

[9] Newman DJ, Cragg GM. Natural products as sources of new drugs over the nearly four decades from 01/1981 to 09/2019. J Nat Prod. 2020;83:770–803. 10.1021/acs.jnatprod.9b01285. 32162523 10.1021/acs.jnatprod.9b01285

[10] Wright GD. Opportunities for natural products in 21st century antibiotic discovery. Nat Prod Rep. 2017;34:694–701. 10.1039/C7NP00019G. 28569300 10.1039/c7np00019g

[11] Stratton CF, Newman DJ, Tan DS. Cheminformatic comparison of approved drugs from natural product versus synthetic origins. Bioorg Med Chem Lett. 2015;25:4802–7. 10.1016/j.bmcl.2015.07.014. 26254944 10.1016/j.bmcl.2015.07.014 PMC4607632

[12] Porras G, Chassagne F, Lyles JT, Marquez L, Dettweiler M, Salam AM, Samarakoon T, Shabih S, Farrokhi DR, Quave CL. Ethnobotany and the role of plant natural products in antibiotic drug discovery. Chem Rev. 2021;121:3495–560. 10.1021/acs.chemrev.0c00922. 33164487 10.1021/acs.chemrev.0c00922 PMC8183567

[13] Álvarez-Martínez FJ, Barrajón-Catalán E, Herranz-López M, Micol V. Antibacterial plant compounds, extracts and essential oils: an updated review on their effects and putative mechanisms of action. Phytomedicine. 2021;90:153626. 10.1016/j.phymed.2021.153626. 34301463 10.1016/j.phymed.2021.153626

[14] Zhang C-W, Zhong X-J, Zhao Y-S, Rajoka MSR, Hashmi MH, Zhai P, Song X. Antifungal natural products and their derivatives: a review of their activity and mechanism of actions. Pharmacol Res Modern Chin Med. 2023;7:100262. 10.1016/j.prmcm.2023.100262.

[15] Aldholmi M, Marchand P, Ourliac-Garnier I, Le Pape P, Ganesan A. A decade of antifungal leads from natural products: 2010–2019. Pharmaceuticals. 2019. 10.3390/ph12040182. 31842280 10.3390/ph12040182 PMC6958371

[16] Moujir L, Callies O, Sousa PMC, Sharopov F, Seca AML. Applications of sesquiterpene lactones: a review of some potential success cases. Appl Sci (Switzerland). 2020. 10.3390/app10093001.

[17] Ayelen Ramallo I, Salazar MO, García P, Furlan RLE. Chapter 10—chemical diversification of natural product extracts. In: Atta-ur-Rahman, editor. Studies in Natural Products Chemistry. Elsevier; 2019. p. 371–98. 10.1016/B978-0-444-64181-6.00010-3.

[18] Richter MF, Hergenrother PJ. The challenge of converting Gram-positive-only compounds into broad-spectrum antibiotics. Ann N Y Acad Sci. 2019;1435:18–38. 10.1111/nyas.13598. 29446459 10.1111/nyas.13598 PMC6093809

[19] Geddes EJ, Li Z, Hergenrother PJ. An LC-MS/MS assay and complementary web-based tool to quantify and predict compound accumulation in *E. coli*. Nat Protoc. 2021;16:4833–54. 10.1038/s41596-021-00598-y. 34480129 10.1038/s41596-021-00598-y PMC8715754

[20] Onyedibe KI, Nemeth AM, Dayal N, Smith RD, Lamptey J, Ernst RK, Melander RJ, Melander C, Sintim HO. Re-sensitization of multidrug-resistant and colistin-resistant gram-negative bacteria to colistin by povarov/doebner-derived compounds. ACS Infect Dis. 2023;9:283–95. 10.1021/acsinfecdis.2c00417. 36651182 10.1021/acsinfecdis.2c00417 PMC10547215

[21] Caesar LK, Cech NB. Synergy and antagonism in natural product extracts: when 1 + 1 does not equal 2. Nat Prod Rep. 2019;36:869–88. 10.1039/C9NP00011A. 31187844 10.1039/c9np00011a PMC6820002

[22] Eloff JN. Avoiding pitfalls in determining antimicrobial activity of plant extracts and publishing the results. BMC Complement Altern Med. 2019;19:106. 10.1186/s12906-019-2519-3. 31113428 10.1186/s12906-019-2519-3 PMC6530048

[23] González U, Morales-Jiménez J, Nieto-Camacho A, Martínez M, Maldonado E. Elemenolides from Zinnia peruviana and evaluation of their antibacterial and α-glucosidase inhibitory activities. Nat Prod Res. 2021;35:1977–84. 10.1080/14786419.2019.1648461. 31401868 10.1080/14786419.2019.1648461

[24] Julianti E, Jang KH, Lee S, Lee D, Mar W, Oh K-B, Shin J. Sesquiterpenes from the leaves of *Laurus nobilis* L. Phytochemistry. 2012;80:70–6. 10.1016/j.phytochem.2012.05.013. 22683316 10.1016/j.phytochem.2012.05.013

[25] Jamuna S, Karthika K, Paulsamy S, Thenmozhi K, Kathiravan S, Venkatesh R. Confertin and scopoletin from leaf and root extracts of Hypochaeris radicata have anti-inflammatory and antioxidant activities. Ind Crops Prod. 2015;70:221–30. 10.1016/j.indcrop.2015.03.039.

[26] Olivaro C, Rostan V, Bandera D, Moyna G, Vazquez A. Xanthane sesquiterpenoids from the roots and flowers of Xanthium cavanillesii. Nat Prod Res. 2016;30:2238–42. 10.1080/14786419.2016.1149709. 26936835 10.1080/14786419.2016.1149709

[27] Yuan Z, Zheng X, Zhao Y, Liu Y, Zhou S, Wei C, Hu Y, Shao H. Phytotoxic compounds isolated from leaves of the invasive weed Xanthium spinosum. Molecules. 2018. 10.3390/molecules23112840. 30388777 10.3390/molecules23112840 PMC6278460

[28] Kummer DA, Brenneman JB, Martin SF. Application of a domino intramolecular enyne metathesis/cross metathesis reaction to the total synthesis of (+)-8-epi-xanthatin. Org Lett. 2005;7:4621–3. 10.1021/ol051711a. 16209494 10.1021/ol051711a

[29] Marco JA, Sanz-Cervera JF, Corral J, Carda M, Jakupovic J. Xanthanolides from Xanthium: absolute configuration of xanthanol, isoxanthanol and their C-4 epimers. Phytochemistry. 1993;34:1569–76. 10.1016/S0031-9422(00)90847-1.

[30] Yang L, Wen K-S, Ruan X, Zhao Y-X, Wei F, Wang Q. Response of plant secondary metabolites to environmental factors. Molecules. 2018. 10.3390/molecules23040762. 29584636 10.3390/molecules23040762 PMC6017249

[31] Verma N, Shukla S. Impact of various factors responsible for fluctuation in plant secondary metabolites. J Appl Res Med Aromat Plants. 2015;2:105–13. 10.1016/j.jarmap.2015.09.002.

[32] Pavarini DP, Pavarini SP, Niehues M, Lopes NP. Exogenous influences on plant secondary metabolite levels. Anim Feed Sci Technol. 2012;176:5–16. 10.1016/j.anifeedsci.2012.07.002.

[33] Tsankova ET, Trendafilova AB, Kujumgiev AI, Galabov AS, Robeva PR. Xanthanolides of *Xanthium italicum* Moretti and their biological activity. Zeitschrift für Naturforschung C. 1994;49:154–6. 10.1515/znc-1994-1-223. 10.1515/znc-1994-1-223 8148005

[34] Sato Y, Oketani H, Yamada T, Singyouchi K-I, Ohtsubo T, Kihara M, Shibata H, Higuti T. a Xanthanolide with potent antibacterial activity against methicillin-resistant *Staphylococcus aureus*. J Pharmacy Pharmacol. 1997;49:1042–4. 10.1111/j.2042-7158.1997.tb06038.x. 10.1111/j.2042-7158.1997.tb06038.x 9364417

[35] Lavault M, Landreau A, Larcher G, Bouchara J-P, Pagniez F, Le Pape P, Richomme P. Antileishmanial and antifungal activities of xanthanolides isolated from Xanthium macrocarpum. Fitoterapia. 2005;76:363–6. 10.1016/j.fitote.2005.03.019. 15890467 10.1016/j.fitote.2005.03.019

[36] Yang C, Li Y, Zhang Y, Hu Q, Liu Y, Li Y, Shi H, Song L, Cao H, Hao X, Zhi X. Natural Sesquiterpene lactone as source of discovery of novel fungicidal candidates: structural modification and antifungal activity evaluation of xanthatin derived from *Xanthium strumarium* L. J Agric Food Chem. 2023;71:11239–51. 10.1021/acs.jafc.3c02435. 37449982 10.1021/acs.jafc.3c02435

[37] Zhi X, Song L, Liang J, Wei S, Li Y, Zhang Y, Hao X, Cao H, Yang C. Synthesis and in vitro antifungal activity of new Michael-type amino derivatives of xanthatin, a natural sesquiterpene lactone from *Xanthium strumarium* L. Bioorg Med Chem Lett. 2022;55:128481. 10.1016/j.bmcl.2021.128481. 34852242 10.1016/j.bmcl.2021.128481

[38] Carlucci R, Lisa M-N, Labadie GR. 1,2,3-Triazoles in biomolecular crystallography: a geometrical data-mining approach. J Med Chem. 2023;66:14377–90. 10.1021/acs.jmedchem.3c01097. 37903297 10.1021/acs.jmedchem.3c01097

[39] Castro SJ, Padrón JM, Darses B, Nicotra VE, Dauban P. Late-stage Rh(II)-catalyzed nitrene transfer for the synthesis of guaianolide analogs with enhanced antiproliferative activity. Eur J Org Chem. 2021;2021:1859–63. 10.1002/ejoc.202100074.

[40] Ayaz M, Ullah F, Sadiq A, Ullah F, Ovais M, Ahmed J, Devkota HP. Synergistic interactions of phytochemicals with antimicrobial agents: potential strategy to counteract drug resistance. Chem Biol Interact. 2019;308:294–303. 10.1016/j.cbi.2019.05.050. 31158333 10.1016/j.cbi.2019.05.050

[41] Ma C-M, Abe T, Komiyama T, Wang W, Hattori M, Daneshtalab M. Synthesis, anti-fungal and 1,3-β-d-glucan synthase inhibitory activities of caffeic and quinic acid derivatives. Bioorg Med Chem. 2010;18:7009–14. 10.1016/j.bmc.2010.08.022. 20813534 10.1016/j.bmc.2010.08.022

[42] Clinical and Laboratory Standards Institute (CLSI), 2006. Methods for Dilution Antimicrobial Susceptibility Tests for Bacteria That Grow Aerobically; Approved Standard—Seventh Edition. Clinical and Laboratory Standards Institute document M7-A7 [ISBN 1-56238-587-9]. Vol. 29, Clinical and Laboratory Standards Institute, 940 West Valley Road, Suite 1400, Wayne, Pennsylvania 19087–1898 USA., (n.d.).

[43] Coutinho HDM, Costa JGM, Falcão-Silva VS, Siqueira-Júnior JP, Lima EO. Potentiation of antibiotic activity by Eugenia uniflora and Eugenia jambolanum. J Med Food. 2010;13:1024–6. 10.1089/jmf.2009.0158. 20482280 10.1089/jmf.2009.0158

