# Supplementary material for: Rational search for natural antimicrobial compounds: relevance of sesquiterpene lactones
Source: Nat Prod Bioprospect. 2025 May 8;15(1):28. doi: 10.1007/s13659-025-00513-y (PMC12061821; doi:10.1007/s13659-025-00513-y)

***Supporting Information***

**Rational search for natural antimicrobial compounds: Relevance of sesquiterpene lactones**

Alejandro Recio-Balsells, Eugenia Rodriguez Ristau, Adriana Pacciaroni, Viviana Nicotra, Carina Casero and Manuela García^*^

Instituto Multidisciplinario de Biología Vegetal (IMBIV), Consejo Nacional de Investigaciones Científicas y Técnicas (CONICET), Córdoba, Argentina. Facultad de Ciencias Químicas, Universidad Nacional de Córdoba (UNC), Ciudad Universitaria, X5000HUA, Córdoba, Argentina

*Corresponding Author

E-mail: manuelagarcia@unc.edu.ar

**Table S1.** Antimicrobial activity against gram-positive and negative strains of extracts of the selected species.

| ***Family*** | ***N°*** | ***Plant species*** | **Herbarium specimen code** | **Fraction** | **MIC (mg/mL)** | |
| --- | --- | --- | --- | --- | --- | --- |
|  |  |  |  |  | ***S. aureus* ATCC 25923** | ***E. coli***  **ATCC 25922** |
| Amaranthaceae | 1 | *Gomphrena perennis* L. | CORD105005 | Total | >1 | >1 |
|  |  |  |  | Hex | >1 | >1 |
|  |  |  |  | AcOEt | >1 | >1 |
|  | 2 | *Gomphrena pulchella* Mart. | CORD105010 | Total | >1 | >1 |
|  |  |  |  | Hex | >1 | >1 |
|  |  |  |  | AcOEt | >1 | >1 |
|  | 3 | *Salsola kali* L. var. kali | CORD00105433 | Total | >1 | >1 |
|  |  |  |  | Hex | >1 | >1 |
|  |  |  |  | AcOEt | >1 | >1 |
| Anacardiaceae | 4 | *Schinus areira* L. | CORD00105420 | Total | >1 | >1 |
|  |  |  |  | Hex | >1 | >1 |
|  |  |  |  | AcOEt | >1 | >1 |
| Apocynaceae | 5 | *Cynanchum bonariense* (Decne.) T. Mey. | CORD120383 | Total | >1 | >1 |
|  |  |  |  | Hex | >1 | >1 |
|  |  |  |  | AcOEt | >1 | >1 |
| Araliaceae | 6 | *Hydrocotyle bonariensis* Lam*.* | CORD104997 | Total | >1 | >1 |
|  |  |  |  | Hex | >1 | >1 |
|  |  |  |  | AcOEt | **1** | >1 |
| Asteraceae | 7 | *Ambrosia tenuifolia* Spreng. | CORD00098343 | Total | >1 | >1 |
|  |  |  |  | Hex | **0.5** | >1 |
|  |  |  |  | AcOEt | >1 | >1 |
|  | 8 | *Artemisia annua* |  | Total | >1 | >1 |
|  |  |  |  | Hex | >1 | >1 |
|  |  |  |  | AcOEt | >1 | >1 |
|  | 9 | *Austroliabum candidum* (Griseb.) H. Rob. & Brettell | CORD00105411 | Total | >1 | >1 |
|  |  |  |  | Hex | >1 | >1 |
|  |  |  |  | AcOEt | **0.5** | >1 |
|  | 10 | *Baccharis articulata* (Lam.) Pers. | CORD00105412 | Total | >1 | >1 |
|  |  |  |  | Hex | **1** | >1 |
|  |  |  |  | AcOEt | **1** | >1 |
|  | 11 | *Baccharis coridifolia* DC. | CORD00105410 | Total | >1 | >1 |
|  |  |  |  | Hex | **0.25** | >1 |
|  |  |  |  | AcOEt | >1 | >1 |
|  | 12 | *Baccharis linearifolia* (Lam.) Pers. | CORD00105436 | Total | **1** | >1 |
|  |  |  |  | Hex | >1 | >1 |
|  |  |  |  | AcOEt | >1 | >1 |
|  | 13 | *Doniophyton anomalum* (D. Don) Kurtz | CORD00056278 | Total | >1 | >1 |
|  |  |  |  | Hex | >1 | >1 |
|  |  |  |  | AcOEt | **1** | >1 |
|  | 14 | *Fleischmannia prasiifolia* (Griseb.) R.M. King & H. Rob. | CORD00105427 | Total | >1 | >1 |
|  |  |  |  | Hex | >1 | >1 |
|  |  |  |  | AcOEt | >1 | >1 |
|  | 15 | *Hypochaeris radicata* L. | CORD00105415 | Total | >1 | >1 |
|  |  |  |  | Hex | >1 | >1 |
|  |  |  |  | AcOEt | **1** | >1 |
|  | 16 | *Lessingianthus mollissimus* (D. Don ex Hook. & Arn.) H. Rob*.* | CORD00105416 | Total | >1 | >1 |
|  |  |  |  | Hex | >1 | >1 |
|  |  |  |  | AcOEt | >1 | >1 |
|  | 17 | *Schkuhria pinnata* (Lam.) Kuntze ex Thell. | CORD105000 | Total | >1 | >1 |
|  |  |  |  | Hex | >1 | >1 |
|  |  |  |  | AcOEt | >1 | >1 |
|  | 18 | *Stevia achalensis* Hieron. | CORD00040412 | Total | >1 | >1 |
|  |  |  |  | Hex | >1 | >1 |
|  |  |  |  | AcOEt | >1 | >1 |
|  | 19 | *Stevia yaconensis* Hieron var. subeglandulosa | CORD00041098 | Total | >1 | >1 |
|  |  |  |  | Hex | **1** | >1 |
|  |  |  |  | AcOEt | >1 | >1 |
|  | 20 | *Solidago chilensis* Meyen* | CORD0009834 | Total | >1 | >1 |
|  |  |  |  | Hex | >1 | >1 |
|  |  |  |  | AcOEt | >1 | >1 |
|  | 21 | *Verbesina encelioides (Cav.) Benth. & Hook ex A. Gray* | CORD105004 | Total | >1 | >1 |
|  |  |  |  | Hex | >1 | >1 |
|  |  |  |  | AcOEt | >1 | >1 |
|  | 22 | *Xanthium cavanillesii* Schouw*.* | CORD00105430 | Total | **1** | >1 |
|  |  |  |  | Hex | >1 | >1 |
|  |  |  |  | AcOEt | **0.25** | >1 |
|  | 23 | *Xanthium spinosum* L. | CORD00111674 | Total | **1** | >1 |
|  |  |  |  | Hex | >1 | >1 |
|  |  |  |  | AcOEt | **0.5** | >1 |
|  | 24 | *Zinnia peruviana* (L.) L. | CORD105002 | Total | >1 | >1 |
|  |  |  |  | Hex | >1 | >1 |
|  |  |  |  | AcOEt | **0.5** | >1 |
| Berberidaceae | 25 | *Nandina domestica* | CORD00105419 | Total | >1 | >1 |
|  |  |  |  | Hex | >1 | >1 |
|  |  |  |  | AcOEt | >1 | >1 |
| Cannabaceae | 26 | *Celtis tala* Gill. ex Planch. | CORD00105349 | Total | >1 | >1 |
|  |  |  |  | Hex | >1 | >1 |
|  |  |  |  | AcOEt | >1 | >1 |
| Celastraceae | 27 | *Maytenus vitis idaea* Griseb. | CORD00048267 | Total | >1 | >1 |
|  |  |  |  | Hex | >1 | >1 |
|  |  |  |  | AcOEt | >1 | >1 |
| Euphorbiaceae | 28 | *Acalypha variabilis* Klotzsch ex Baill. | CORD00105413 | Total | **0.5-0.25** | >1 |
|  |  |  |  | Hex | >1 | >1 |
|  |  |  |  | AcOEt | >1 | >1 |
|  | 29 | *Euphorbia helioscopia* L. | CORD00105406 | Total | >1 | >1 |
|  |  |  |  | Hex | >1 | >1 |
|  |  |  |  | AcOEt | >1 | >1 |
|  | 30 | *Jatropha excisa* Griseb. var. excisa | CORD105008 | Total | >1 | >1 |
|  |  |  |  | Hex | >1 | >1 |
|  |  |  |  | AcOEt | >1 | >1 |
| Fabaceae | 31 | *Bauhinia forficata* Benth | CORD00103312 | Total | >1 | >1 |
|  |  |  |  | Hex | >1 | >1 |
|  |  |  |  | AcOEt | >1 | >1 |
|  | 32 | *Otholobium higuerilla* (Gillies ex Hook.) J.W. Grimes | CORD00105414 | Total | >1 | >1 |
|  |  |  |  | Hex | >1 | >1 |
|  |  |  |  | AcOEt | >1 | >1 |
|  | 33 | *Senna aphylla* (Cav.) H.S. Irwin & Barneby var. Aphylla | CORD00105007 | Total | >1 | >1 |
|  |  |  |  | Hex | >1 | >1 |
|  |  |  |  | AcOEt | >1 | >1 |
|  | 34 | *Vachellia caven* (Molina) Seigler & Ebinger | CORD00104754 | Total | **1** | >1 |
|  |  |  |  | Hex | >1 | >1 |
|  |  |  |  | AcOEt | >1 | >1 |
| Lamiaceae | 35 | *Rosmarinus officinalis* L. | CORD00019479 | Total | **0.5** | >1 |
|  |  |  |  | Hex | >1 | >1 |
|  |  |  |  | AcOEt | >1 | >1 |
| Lauraceae | 36 | *Laurus nobilis* L. | CORD00075384 | Total | **0.5** | >1 |
|  |  |  |  | Hex | >1 | >1 |
|  |  |  |  | AcOEt | **0.25** | >1 |
| Loranthaceae | 37 | *Ligaria cuneifolia* (Ruiz & Pav.) Tiegh. | CORD105011 | Total | >1 | >1 |
|  |  |  |  | Hex | >1 | >1 |
|  |  |  |  | AcOEt | >1 | >1 |
| Mimosoideae | 38 | *Senegalia praecox (Griseb.) Seigler & Ebinger* | CORD105003 | Total | >1 | >1 |
|  |  |  |  | Hex | >1 | >1 |
|  |  |  |  | AcOEt | >1 | >1 |
| Nyctaginaceae | 39 | *Boerhavia diffusa L. var. leiocarpa* (Heim.) Adams | CORD105006 | Total | >1 | >1 |
|  |  |  |  | Hex | >1 | >1 |
|  |  |  |  | AcOEt | >1 | >1 |
|  | 40 | *Mirabilis jalapa* L. | CORD00105431 | Total | >1 | >1 |
|  |  |  |  | Hex | >1 | >1 |
|  |  |  |  | AcOEt | >1 | >1 |
| Paulowniaceae | 41 | *Paulownia tomentosa* (Thund.) Steud. | CORD00105424 | Total | >1 | >1 |
|  |  |  |  | Hex | >1 | >1 |
|  |  |  |  | AcOEt | >1 | >1 |
| Ranunculaceae | 42 | *Clematis montevidensis* Spreng. *var. montevidensis* | CORD105009 | Total | >1 | >1 |
|  |  |  |  | Hex | >1 | >1 |
|  |  |  |  | AcOEt | **1** | >1 |
|  | 43 | *Anemone decapetala Ard. var. decapetala* | CORD00105407 | Total | **1** | >1 |
|  |  |  |  | Hex | >1 | >1 |
|  |  |  |  | AcOEt | **0.25** | >1 |
| Rutaceae | 44 | *Ruta chalepensis* L. | CORD104998 | Total | >1 | >1 |
|  |  |  |  | Hex | >1 | >1 |
|  |  |  |  | AcOEt | **0.5** | >1 |
| Solanaceae | 45 | *Cestrum parqui* L'Hér. | CORD00105428 | Total | >1 | >1 |
|  |  |  |  | Hex | >1 | >1 |
|  |  |  |  | AcOEt | >1 | >1 |
|  | 46 | *Dunalia brachyacantha* Miers | CORD00022546 | Total | >1 | >1 |
|  |  |  |  | Hex | >1 | >1 |
|  |  |  |  | AcOEt | **1** | >1 |
|  | 47 | *Exodeconus maritimus* (Benth.) D'Arcy | CORD00578445 | Total | >1 | >1 |
|  |  |  |  | Hex | >1 | >1 |
|  |  |  |  | AcOEt | >1 | >1 |
|  | 48 | *Jaborosa integrifolia* Lam. | CORD00022932 | Total | >1 | >1 |
|  |  |  |  | Hex | >1 | >1 |
|  |  |  |  | AcOEt | >1 | >1 |
|  | 49 | *Lycium cestroides* Schltdl. | CORD00105434 | Total | >1 | >1 |
|  |  |  |  | Hex | >1 | >1 |
|  |  |  |  | AcOEt | >1 | >1 |
|  | 50 | *Nicandra physalodes* (L.) Gaertn | CORD00053636 | Total | >1 | >1 |
|  |  |  |  | Hex | >1 | >1 |
|  |  |  |  | AcOEt | >1 | >1 |
|  | 51 | *Nicotiana glauca* Graham | CORD00441165 | Total | >1 | >1 |
|  |  |  |  | Hex | >1 | >1 |
|  |  |  |  | AcOEt | >1 | >1 |
|  | 52 | *Physalis viscosa* L. | CORD00105429 | Total | >1 | >1 |
|  |  |  |  | Hex | >1 | >1 |
|  |  |  |  | AcOEt | >1 | >1 |
|  | 53 | *Sclerophylax arnottii* Miers | CORD00030372 | Total | >1 | >1 |
|  |  |  |  | Hex | >1 | >1 |
|  |  |  |  | AcOEt | >1 | >1 |
|  | 54 | *Sclerophylax caducifructus* Di Fulvio | CORD00050334 | Total | >1 | >1 |
|  |  |  |  | Hex | >1 | >1 |
|  |  |  |  | AcOEt | >1 | >1 |
|  | 55 | *Vassobia breviflora* (Sendtn.) Hunz. | CORD00053634 | Total | >1 | >1 |
|  |  |  |  | Hex | >1 | >1 |
|  |  |  |  | AcOEt | >1 | >1 |
| Urticaceae | 56 | *Urtica major/dioica* |  | Total | >1 | >1 |
|  |  |  |  | Hex | >1 | >1 |
|  |  |  |  | AcOEt | >1 | >1 |
| Verbenaceae | 57 | *Aloysia gratissima* (Gillies & Hook. ex Hook.) Tronc. *var. gratissima* | CORD00105426 | Total | >1 | >1 |
|  |  |  |  | Hex | >1 | >1 |
|  |  |  |  | AcOEt | >1 | >1 |
|  | 58 | *Lantana camara* L. | CORD00100241 | Total | >1 | >1 |
|  |  |  |  | Hex | >1 | >1 |
|  |  |  |  | AcOEt | >1 | >1 |
|  | 59 | *Lippia junelliana* (Mold.) Troncoso | CORD00105417 | Total | **0.5** | >1 |
|  |  |  |  | Hex | >1 | >1 |
|  |  |  |  | AcOEt | >1 | >1 |
| *Only in this case, roots were used for extraction (using Soxhlet). | | | | | | |

**Table S2.** Antibacterial activity of fractions of EtOAc preselected extracts.

| **Species** | **Extract** | **Extract or Fraction** | ***S. aureus* MIC (mg/mL)** |
| --- | --- | --- | --- |
| ***Zinnia peruviana*** | EtOAc | EtOAc | **0.50** |
|  |  | F1 | **1.00** |
|  |  | F2 | **≤0.125** |
|  |  | F3 | **0.50** |
|  |  | F4 | NA |
| ***Laurus nobilis*** | EtOAc | EtOAc | **0.25** |
|  |  | F1 | **0.25** |
|  |  | F2 | **≤0.125** |
|  |  | F3 | **0.50** |
|  |  | F4 | **0.50** |
| ***Hypochaeris radicata*** | EtOAc | EtOAc | **1.00** |
|  |  | F1 | **1.00** |
|  |  | F2 | **0.50** |
|  |  | F3 | NA |
|  |  | F4 | NA |
| ***Xanthium cavanillesii*** | EtOAc | EtOAc | **1.00** |
|  |  | F1 | NA |
|  |  | F2 | **0.50** |
|  |  | F3 | **0.50** |
|  |  | F4 | NA |
| ***Xanthium spinosum*** | EtOAc | EtOAc | **0.50** |
|  |  | F1 | NA |
|  |  | F2 | **0.25** |
|  |  | F3 | **0.25** |
|  |  | F4 | NA |
| **NA: Not active** |  |  |  |

**Structural elucidation of natural undescribed compounds**

NMR spectroscopy of xanthatin and 8-epi-xanthatin shows clear differences that facilitate their distinction **(Table S3)**. These are observed in the ^1^H and ^13^C NMR chemical shift of the CH-8 methine group. In ^1^H NMR, the H-8 in xanthatin appears at 4.30 ppm, while in 8-epi-xanthatin it is at 4.65 ppm. Similarly, in ^13^C NMR, C-8 exhibits a shift at 81.4 ppm for xanthatin and 78.2 ppm for 8-epi-xanthatin. Additionally, for 8-epi-xanthatin NOESY spectra clearly shows an interaction between the H-8 (4.65 ppm) and H-10 (2.83 ppm). Other notable differences in the ^1^H NMR spectra include shifts of the protons H-2, H-3, and H-5, though these are less pronounced than for C-6.

The ^1^H and ^13^C chemical shifts of new isolated compounds (8-epi-isoxanthanol and 8-epi-ivalbin) were very similar to their respective epimers. Similar to xanthatin and 8-epi-xanthatin, the primary distinguishing feature in the spectra were the shift of the H-8, with isoxanthanol and ivalbin showing values of 4.29 and 4.31 ppm, compared to 4.61 and 4.62 ppm for 8-epi-isoxanthanol and 8-epi-ivalbin.

**Table S3.** Main differences in chemical shifts of naturally isolated xanthanolides

|  | **H-8** | **C-8** | **H-2** | **H-3** | **H-5** |
| --- | --- | --- | --- | --- | --- |
| Xanthatin | 4.29 | 81.4 | 7.05 | 6.20 | 6.28 |
| Isoxanthanol | 4.29 | 82.2 | 4.12 | 1.94,1.66 | 5.75 |
| Ivalbin | 4.31 | 82.4 | 4.30 | 1.68, 1.55 | 5.85 |
| 8-epi-xantathin | 4.65 | 78.2 | 6.98 | 6.14 | 6.20 |
| 8-epi-isoxanthanol | 4.61 | 78.8 | 4.10 | 1.97, 1.83 | 5.74 |
| 8-epi-ivalbin | 4.62 | 79.2 | 4.27 | 1.71, 1.59 | 5.82 |

**Table S4.** enTRy rules for natural compounds and chemical derivatives (**1**-**8**). The rules that were not followed are shown in red and the rules that were respected are shown in green.

|  | Primary Amine | Low Globularity | Low flexibility |
| --- | --- | --- | --- |
| 8-epi-xantatina |  |  |  |
| xantatina |  |  |  |
| Isoxantanol |  |  |  |
| epiisoxanth |  |  |  |
| Ivalbin |  |  |  |
| epiivalbin |  |  |  |
| 1 |  |  |  |
| 2 |  |  |  |
| 3 |  |  |  |
| 4 |  |  |  |
| 5 |  |  |  |
| 6 |  |  |  |
| 7 |  |  |  |
| 8 |  |  |  |

**Figure S1.** Minimum Energy Structures (MMFF) of naturally xanthanolides and semisynthetic derivatives **3**, **3´**, **7** and **8**.

| **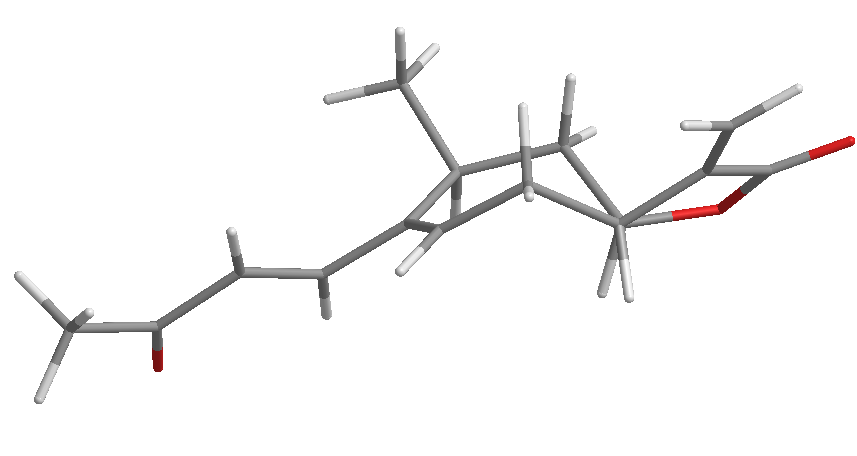** | **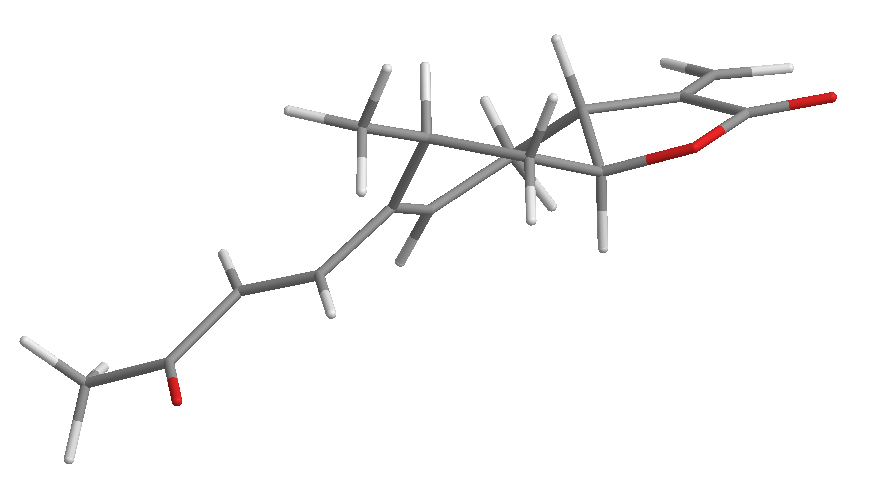** | **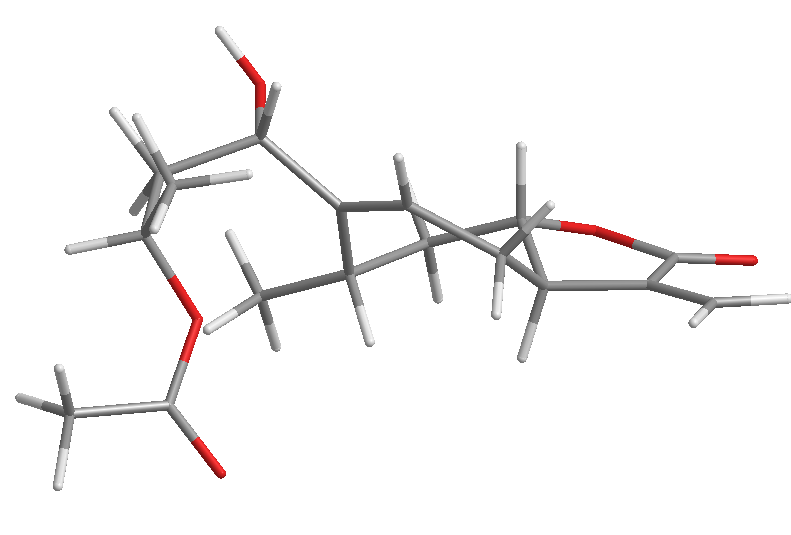** | **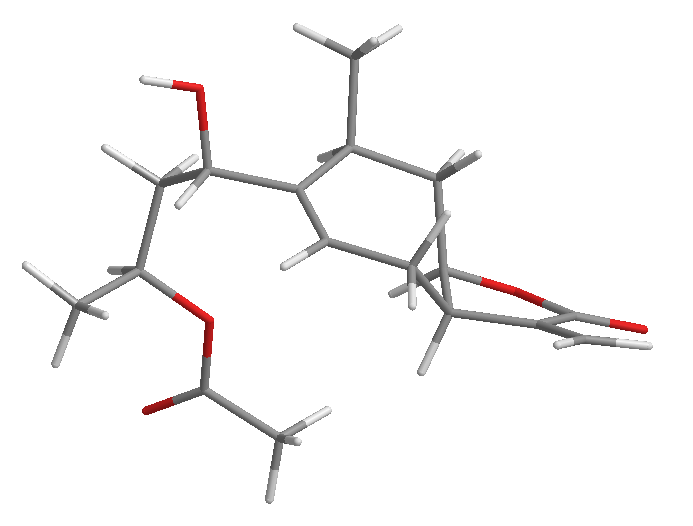** |
| --- | --- | --- | --- |
| **8-epi-xanthatin** | **xanthatin** | **isoxanthanol** | **8-epi-isoxanthanol** |
| **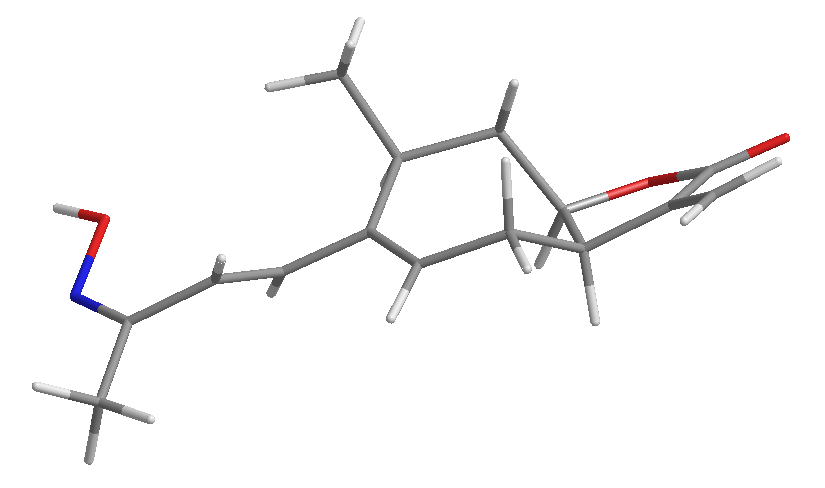** | **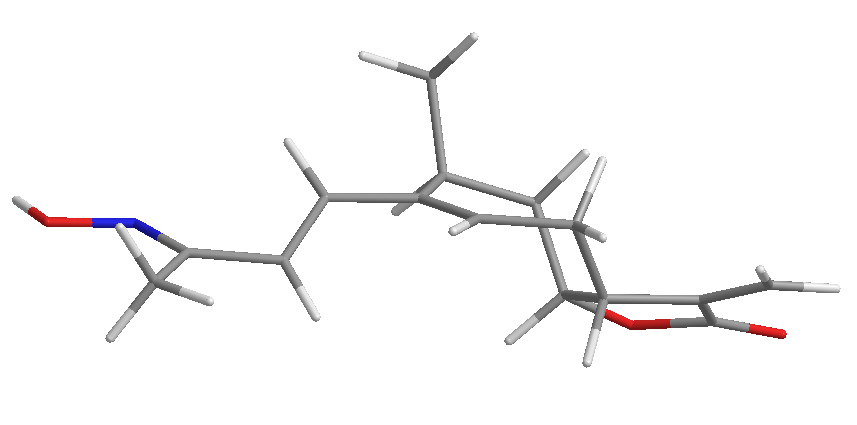** | **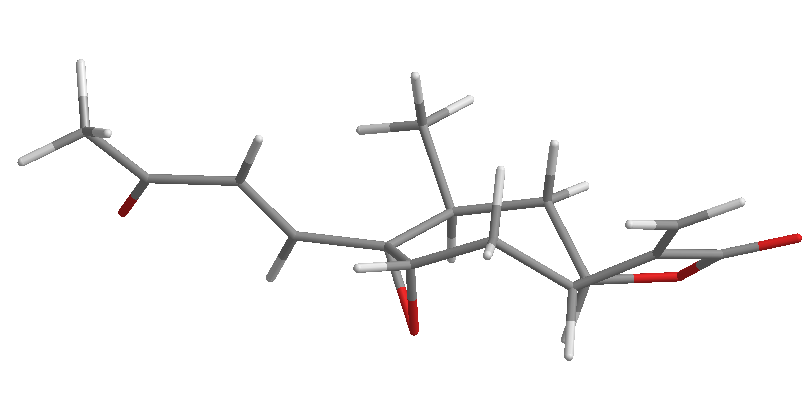** | **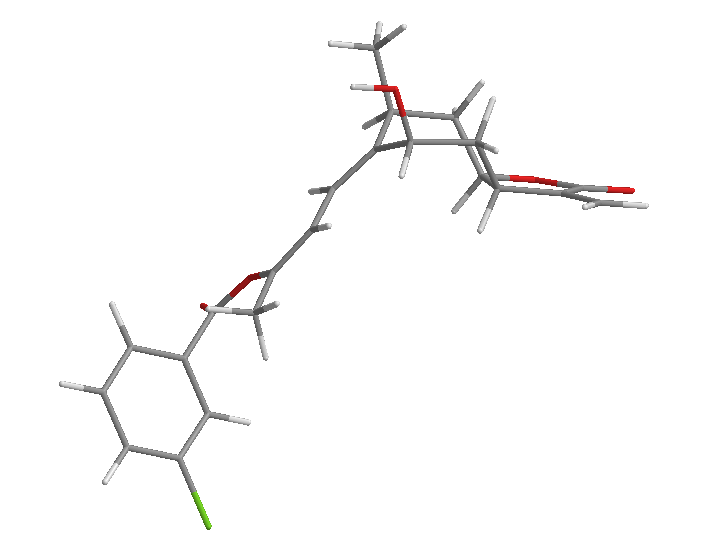** |
| **3** | **3´** | **7** | **8** |

**Table S5.** MIC of natural compounds and semisynthetic derivatives **9** and **10** against *C. albicans* ATCC 10231

| **Compound** | **MIC (mg/mL)** |
| --- | --- |
| Cinnamic acid | 0.25 |
| 4-oxo-4-(p-tolyl)butanoic acid | 0.5 |
| Isoxanthanol | 1 |
| Isoxanthanol + Cinnamic acid (0.125 mg/ml) | 1 |
| Isoxanthanol + 4-oxo-4-(p-tolyl)butanoic acid (0.25 mg/ml) | 1 |
| Compound 9 | >1 |
| Compound 10 | >1 |

**Table S6.** Structural parameters and physicochemical descriptors of the set of compounds obtained

| Compound | Fraction Csp3 | Globularity | PBF (plane of best fit) | N° Rot Bonds | N° HBA | N° HBD | Molar Refractivity | TPSA | Consensus Log P | ESOL Class | PAINS #alerts | Brenk #alerts | Leadlikeness #violations | Synthetic Accessibility |
| --- | --- | --- | --- | --- | --- | --- | --- | --- | --- | --- | --- | --- | --- | --- |
| 8-epi-xanthatin | 0.47 | 0.107 | 0.859 | 2 | 3 | 0 | 70.05 | 43.37 | 2.48 | Soluble | 0 | 1 | 1 | 4.18 |
| Xanthatin | 0.47 | 0.129 | 0.935 | 2 | 3 | 0 | 70.05 | 43.37 | 2.47 | Soluble | 0 | 1 | 1 | 4.18 |
| Isoxanthanol | 0.65 | 0.054 | 0.695 | 5 | 5 | 1 | 82.39 | 72.83 | 2.17 | Soluble | 0 | 3 | 0 | 4.72 |
| 8-epi-isoxanthanol | 0.65 | 0.113 | 0.972 | 5 | 5 | 1 | 82.39 | 72.83 | 2.26 | Soluble | 0 | 3 | 0 | 4.72 |
| Ivalbin | 0.67 | 0.168 | 1.043 | 3 | 4 | 2 | 72.65 | 66.76 | 1.76 | Soluble | 0 | 2 | 0 | 4.53 |
| 8-epi-ivalbina | 0.67 | 0.103 | 0.933 | 3 | 4 | 2 | 72.65 | 66.76 | 1.75 | Soluble | 0 | 2 | 0 | 4.53 |
| (1) | 0.60 | 0.097 | 0.854 | 3 | 4 | 1 | 73.24 | 69.39 | 1.68 | Soluble | 0 | 1 | 0 | 4.24 |
| (2) | 0.60 | 0.081 | 0.763 | 4 | 6 | 0 | 75.35 | 93.12 | 2.18 | Soluble | 1 | 4 | 0 | 4.36 |
| (3) | 0.47 | 0.108 | 0.845 | 2 | 4 | 1 | 74.27 | 58.89 | 2.53 | Soluble | 0 | 4 | 0 | 4.43 |
| (3´) | 0.47 | 0.108 | 0.845 | 2 | 4 | 1 | 74.27 | 58.89 | 2.53 | Soluble | 0 | 4 | 0 | 4.43 |
| (4) | 0.56 | 0.063 | 0.763 | 5 | 4 | 1 | 85.91 | 55.40 | 2.39 | Soluble | 0 | 2 | 0 | 4.42 |
| (5) | 0.44 | 0.053 | 0.979 | 8 | 6 | 1 | 121.95 | 86.11 | 2.98 | Soluble | 0 | 1 | 2 | 4.96 |
| (6) | 0.62 | 0.114 | 0.957 | 4 | 4 | 0 | 76.42 | 52.60 | 2.15 | Soluble | 0 | 1 | 0 | 4.55 |
| (7) | 0.60 | 0.170 | 1.001 | 2 | 4 | 0 | 69.54 | 55.90 | 2.01 | Soluble | 0 | 2 | 0 | 4.37 |
| (8) | 0.36 | 0.164 | 1.284 | 4 | 5 | 1 | 106.83 | 72.83 | 3.90 | Moderately soluble | 0 | 3 | 2 | 4.88 |
| (9) | 0.42 | 0.159 | 1.299 | 9 | 6 | 0 | 121.74 | 78.90 | 4.10 | Moderately soluble | 0 | 3 | 3 | 5.29 |
| (10) | 0.50 | 0.142 | 1.340 | 11 | 7 | 0 | 131.61 | 95.97 | 4.30 | Moderately soluble | 0 | 3 | 3 | 5.33 |

**Table S7.** The bioavalability radar for natural compounds and semisynthetic derivatives. The pink area represents the optimal range for each properties (lipophilicity: XLOGP3 between -0.7 and +5.0, size: MW between 150 and 500 g/mol, polarity: TPSA between 20 and 130 Å2, solubility: log *S* not higher than 6, saturation: fraction of carbons in the sp3 hybridization not less than 0.25, and flexibility: no more than 9 rotatable bonds). For all simulated compounds, adequate oral bioavailability is predicted.

| 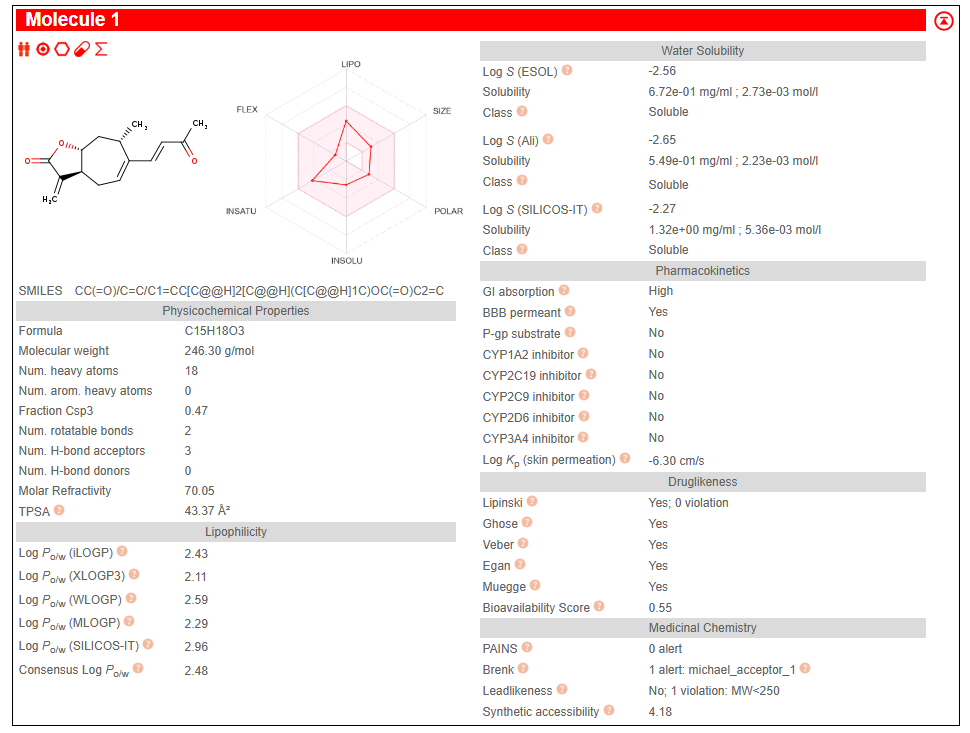 | 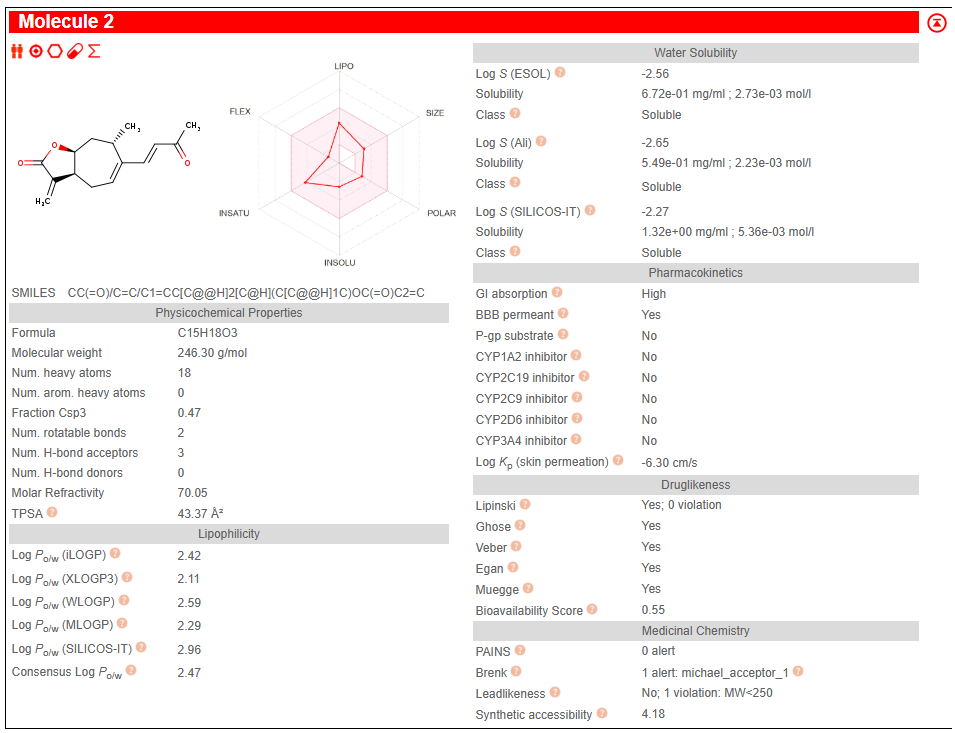 | 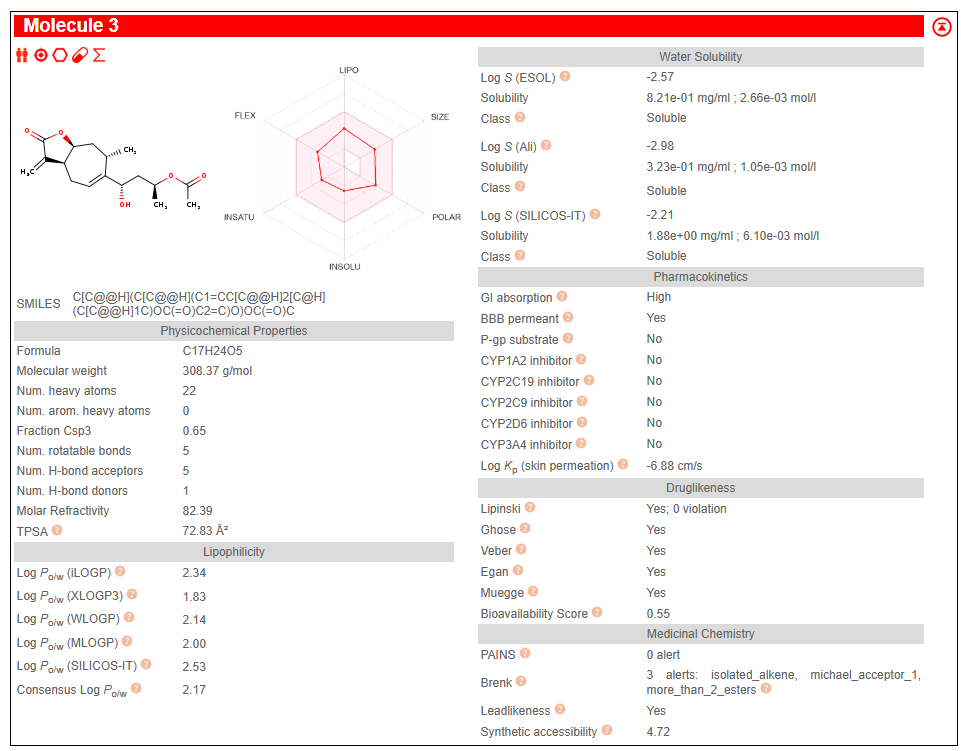 | 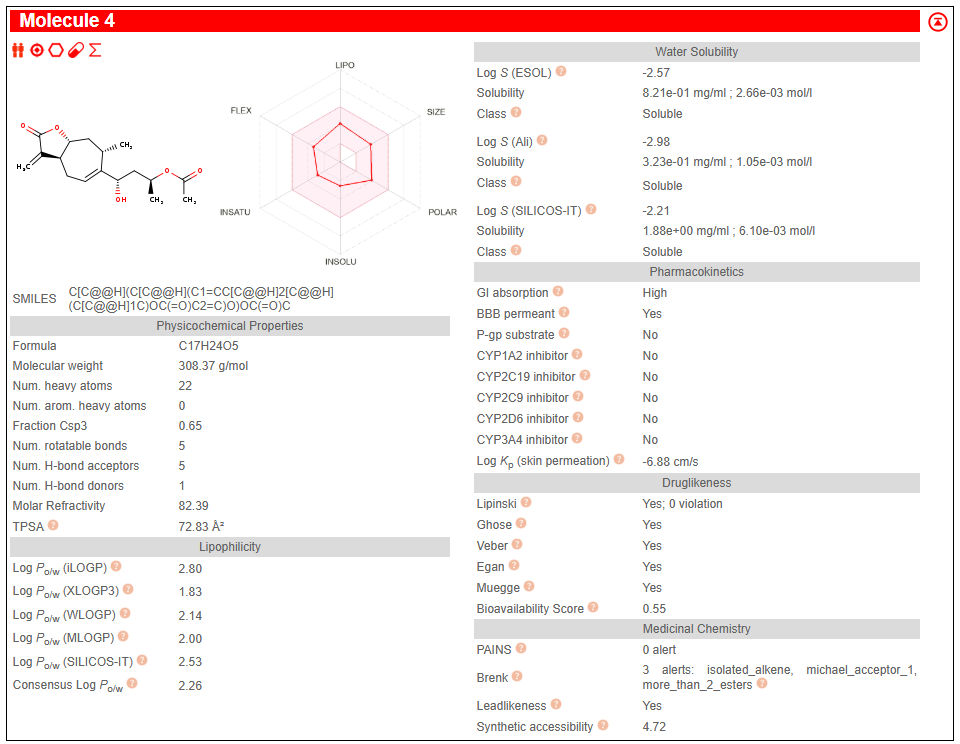 | 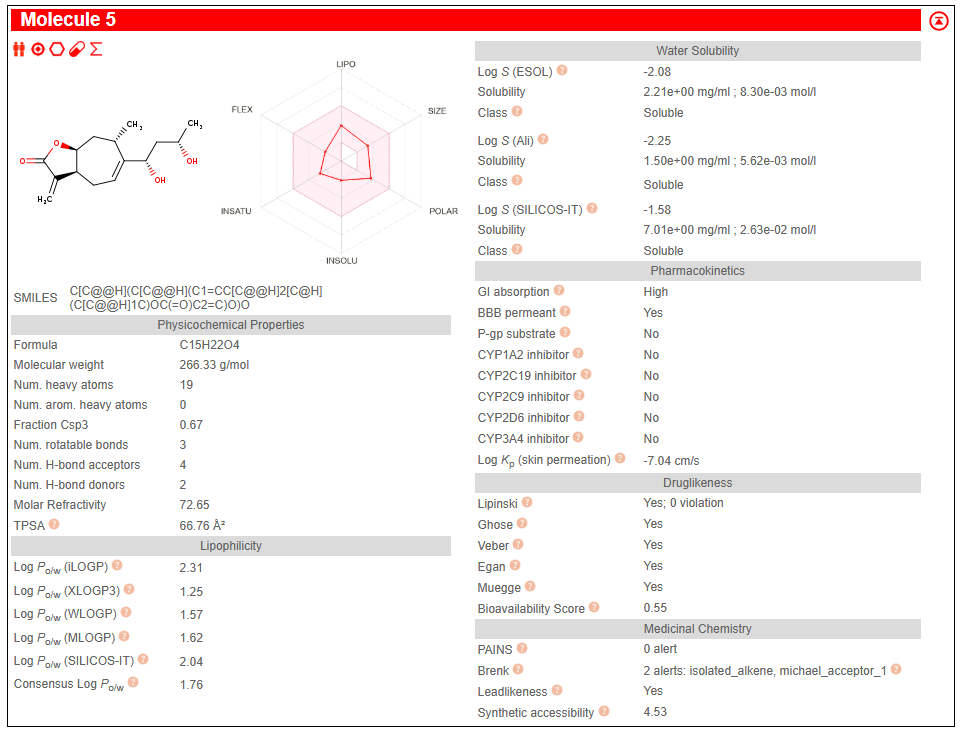 |
| --- | --- | --- | --- | --- |
| 8-epi-xanthatin | Xanthatin | Isoxanthanol | 8-epi-isoxanthanol | Ivalbin |
| 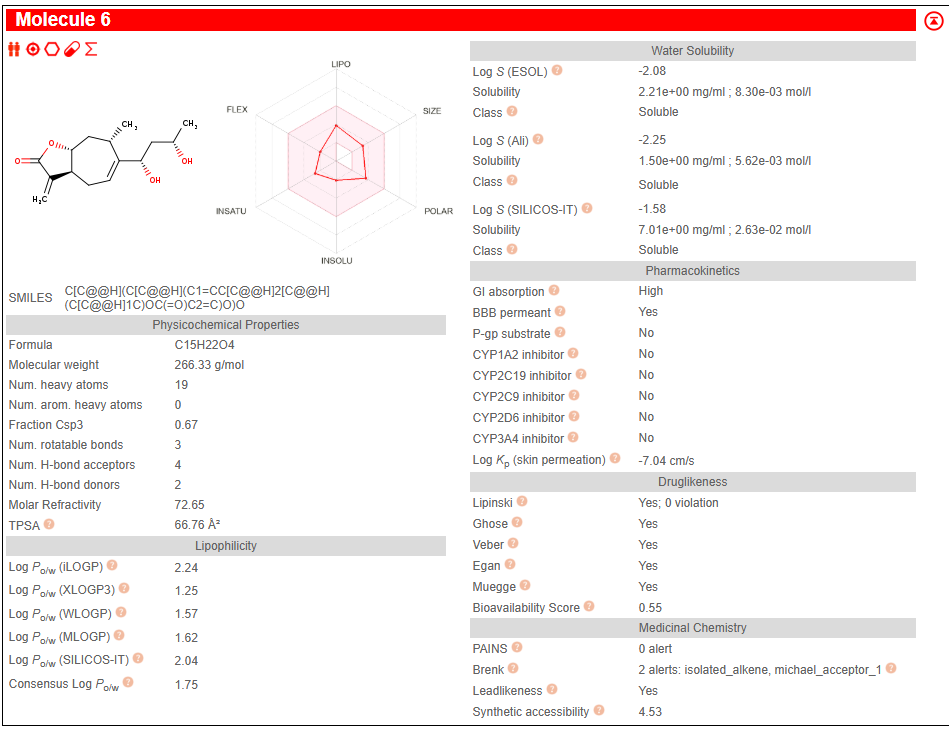 | 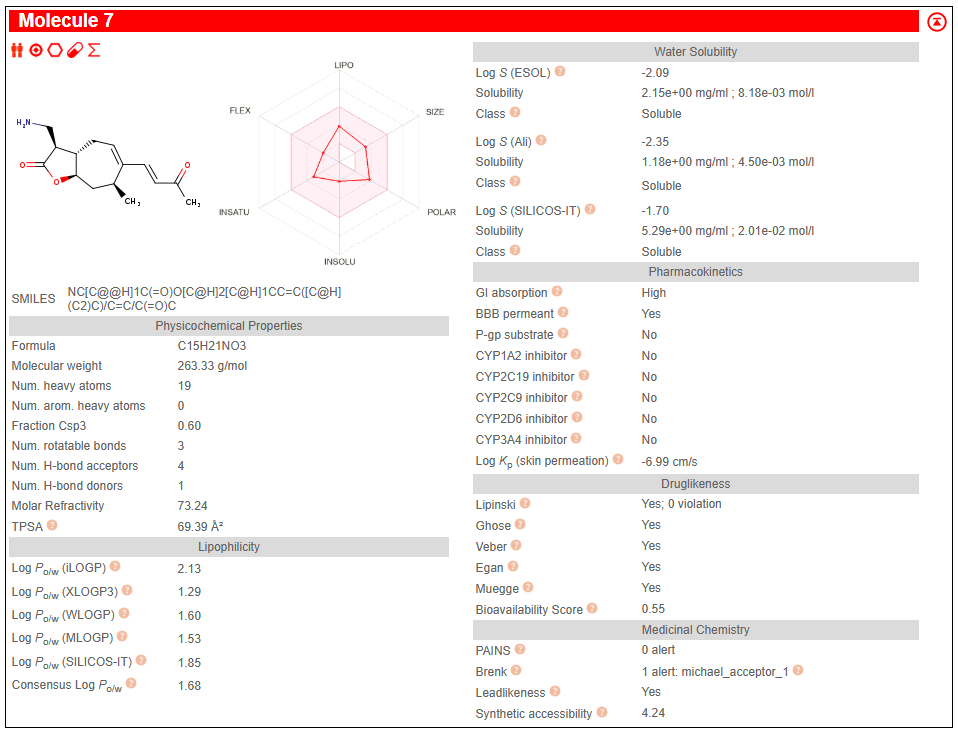 | 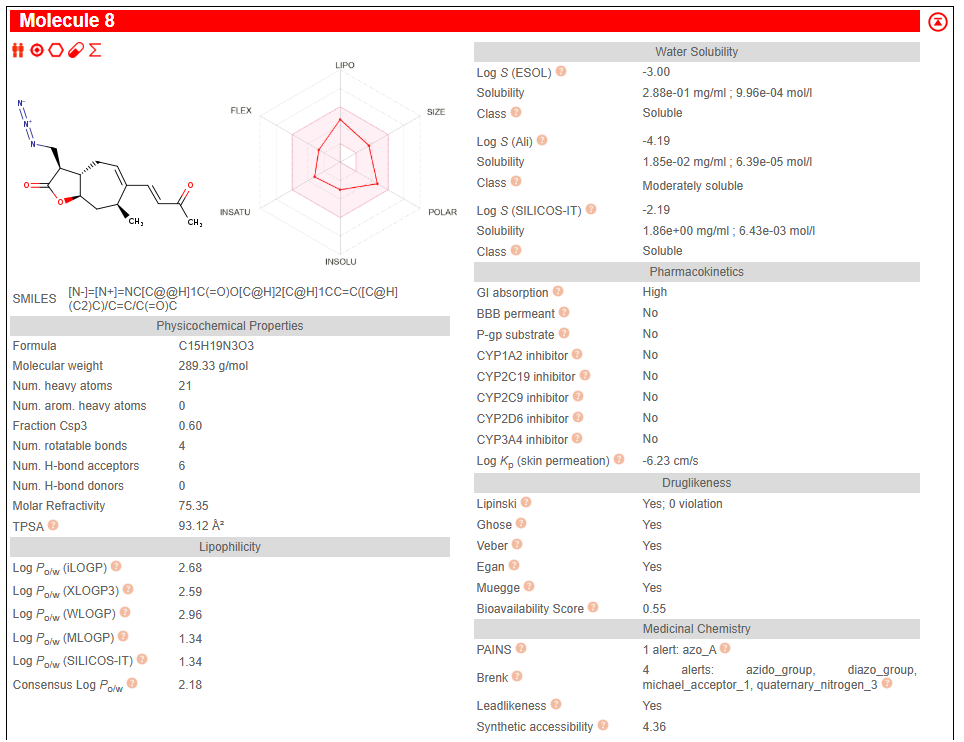 | 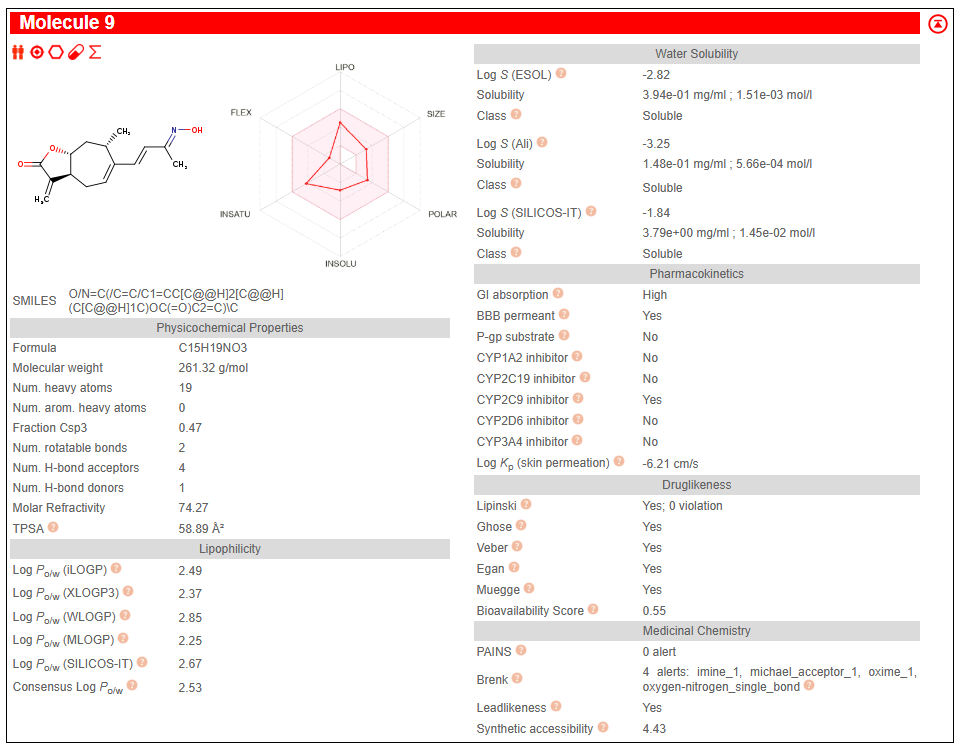 | 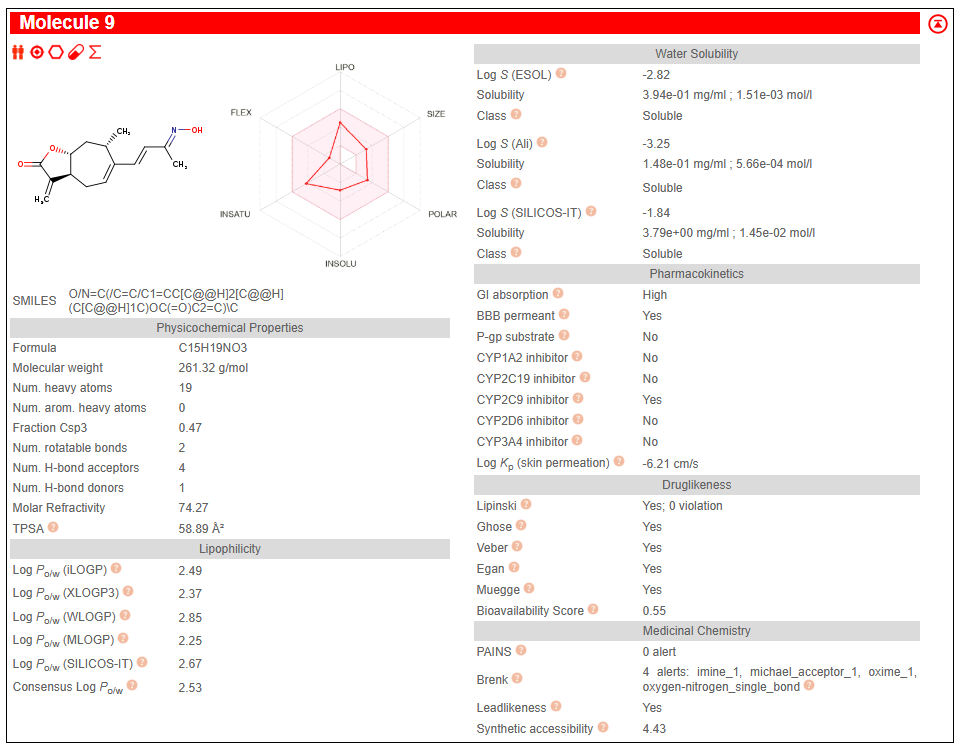 |
| 8-epi-ivalbina | **1** | **2** | **3** | **(3´)** |
| 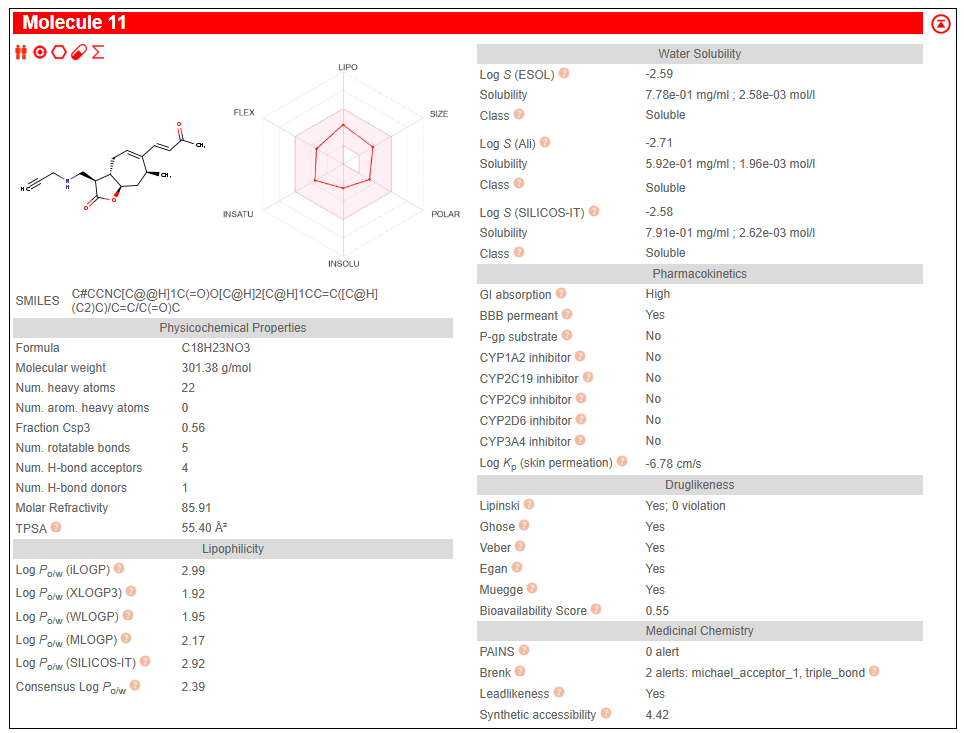 | 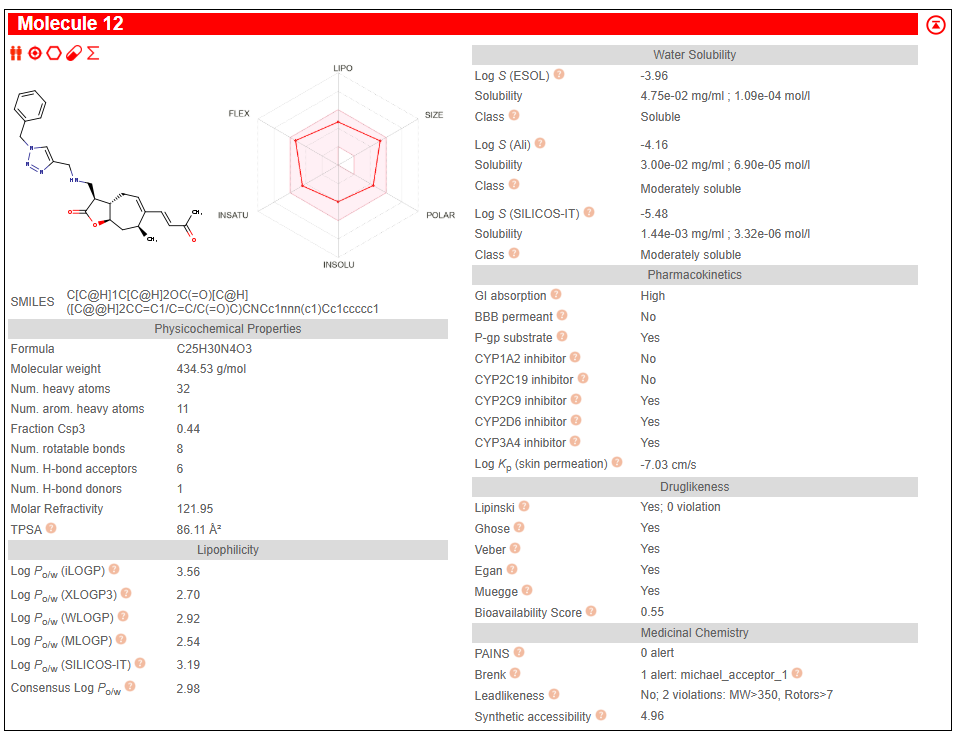 | 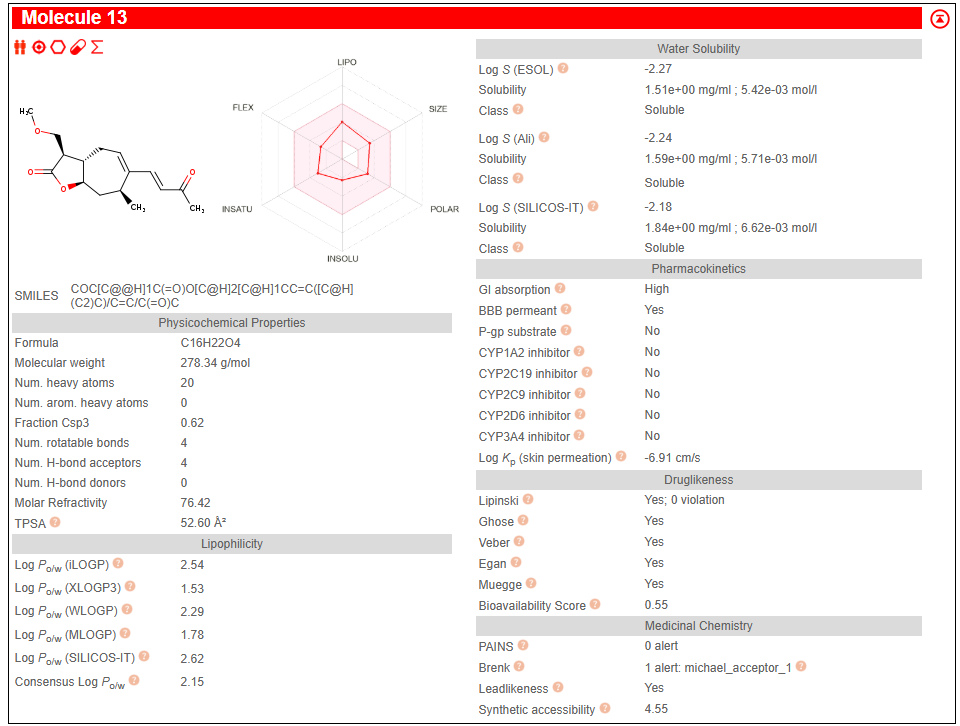 | 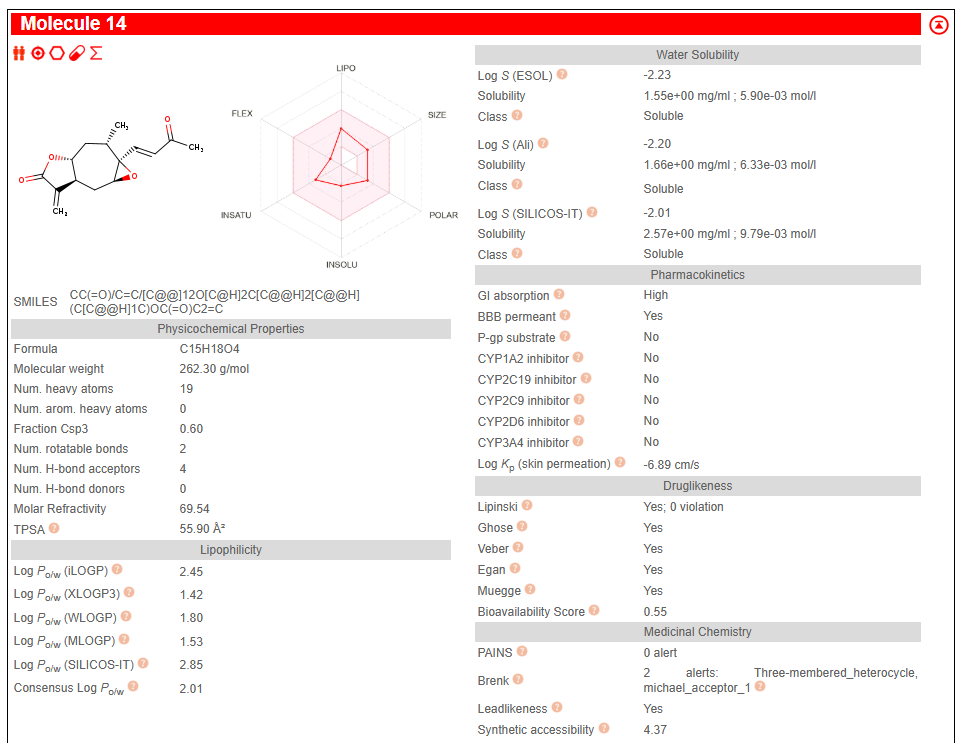 | 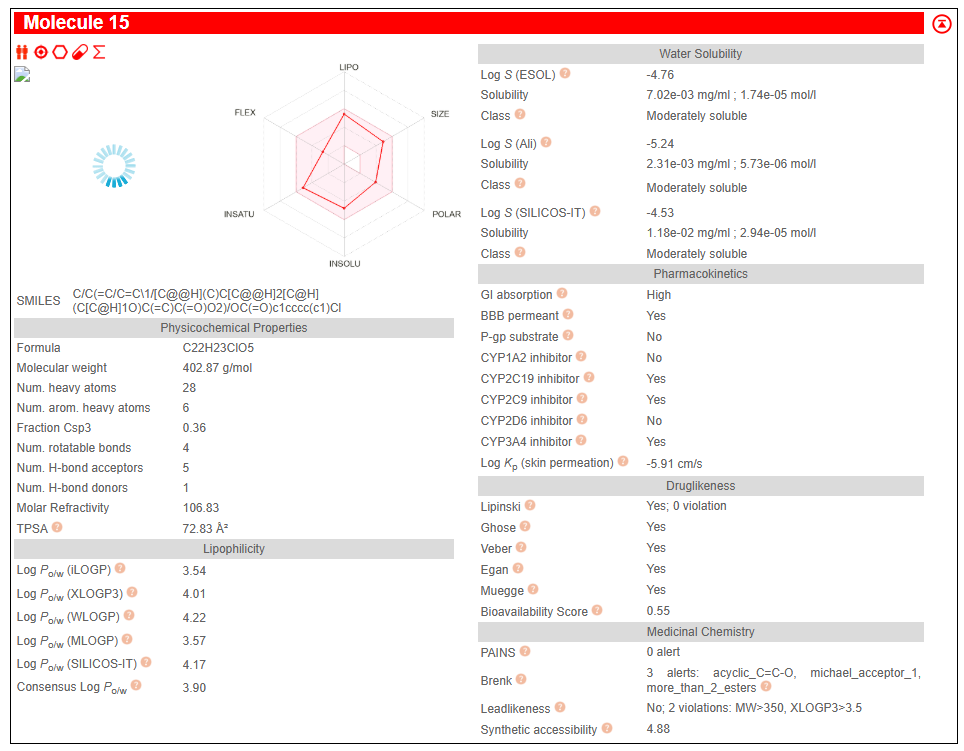 |
| **4** | **5** | **6** | **7** | **8** |
| 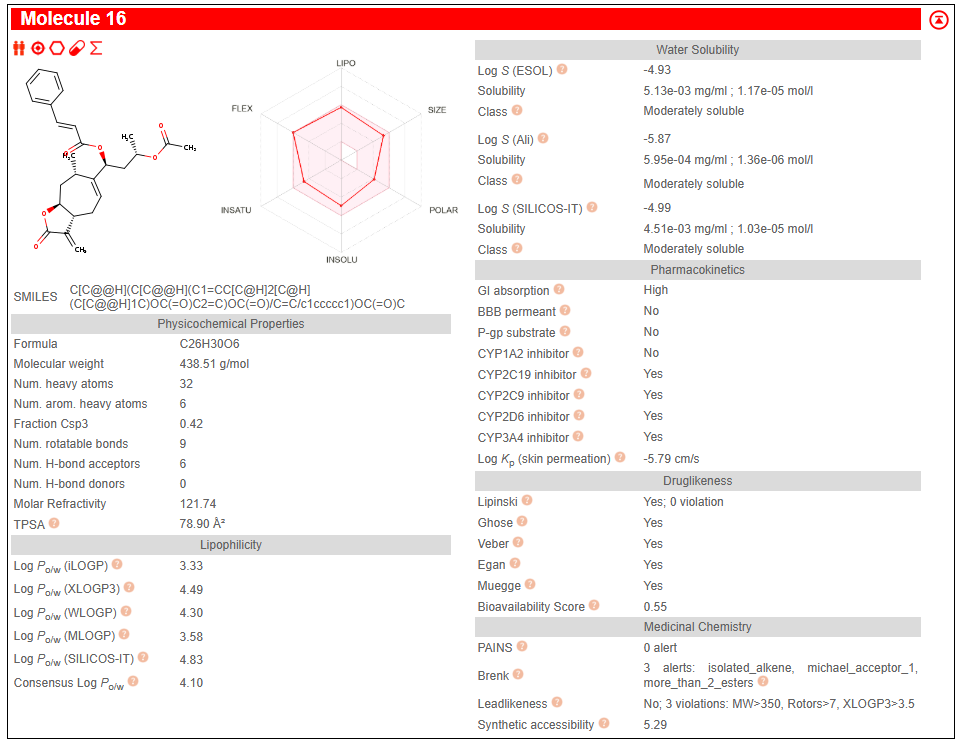 | 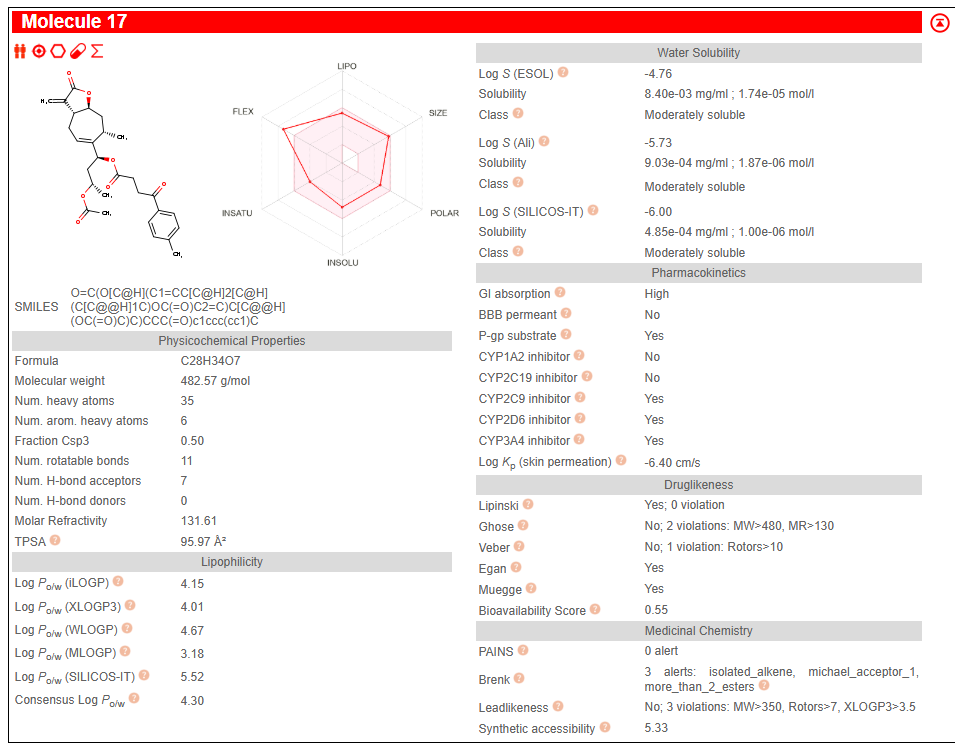 |  |  |  |
| **9** | **10** |  |  |  |

# **Figure S2-1.** ^1^H NMR spectrum of 8-epi-isoxanthanol (CDCl_3_, 400.13 MHz).


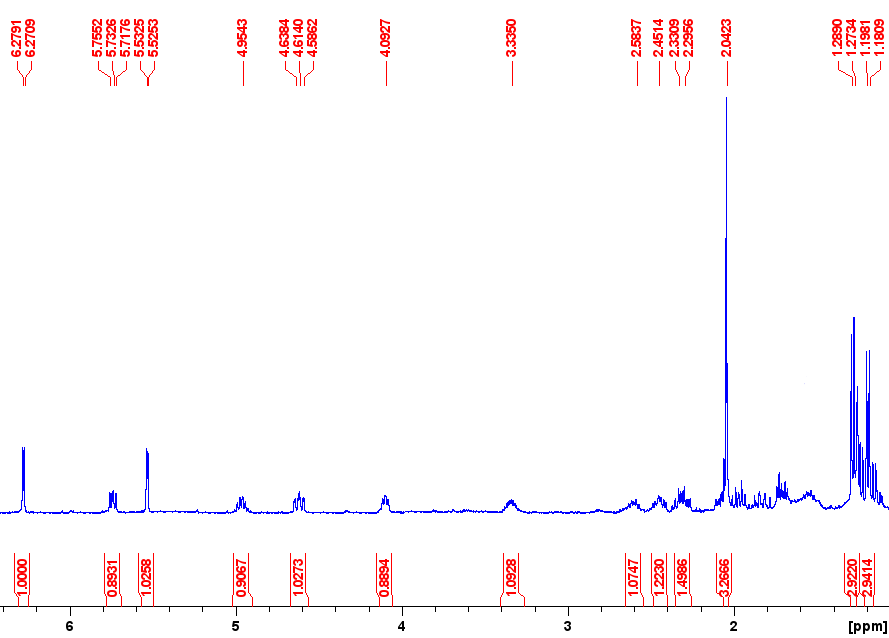

# **Figure S2-2**. COSY spectrum of 8-epi-isoxanthanol (CDCl_3_, 400.13 MHz).


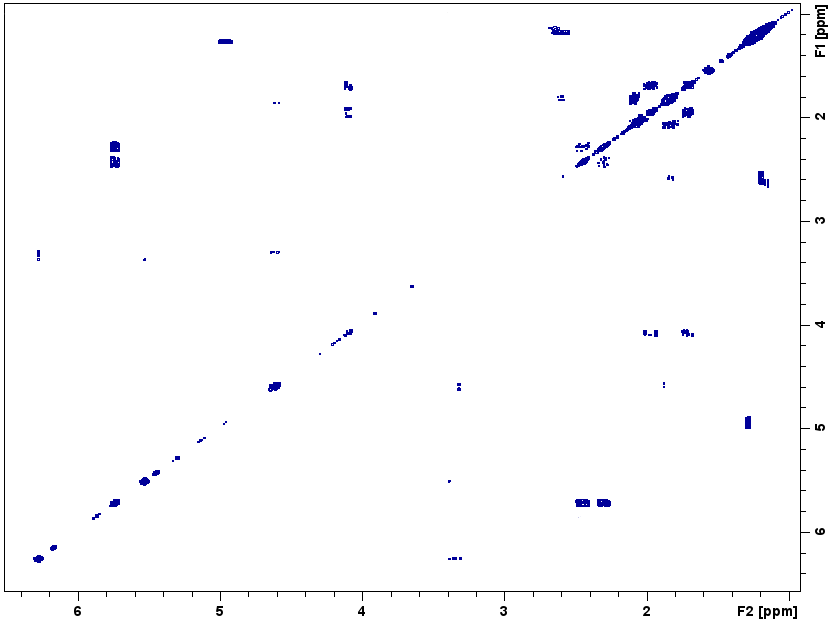

# **Figure S2-4**. HSQC spectrum of 8-epi-isoxanthanol (CDCl_3_, 400.13 MHz).


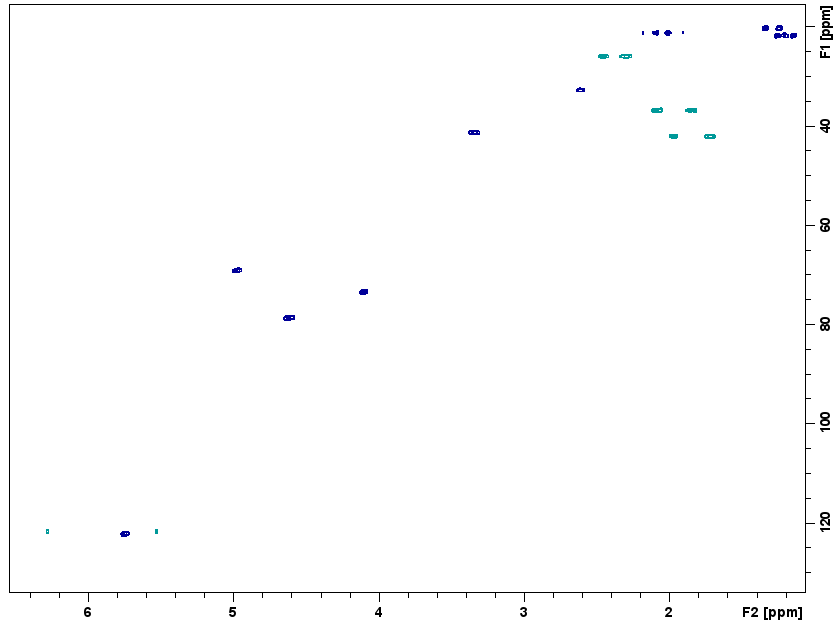

# **Figure S2-5**. HMBC spectrum of 8-epi-isoxanthanol (CDCl_3_, 400.13 MHz).


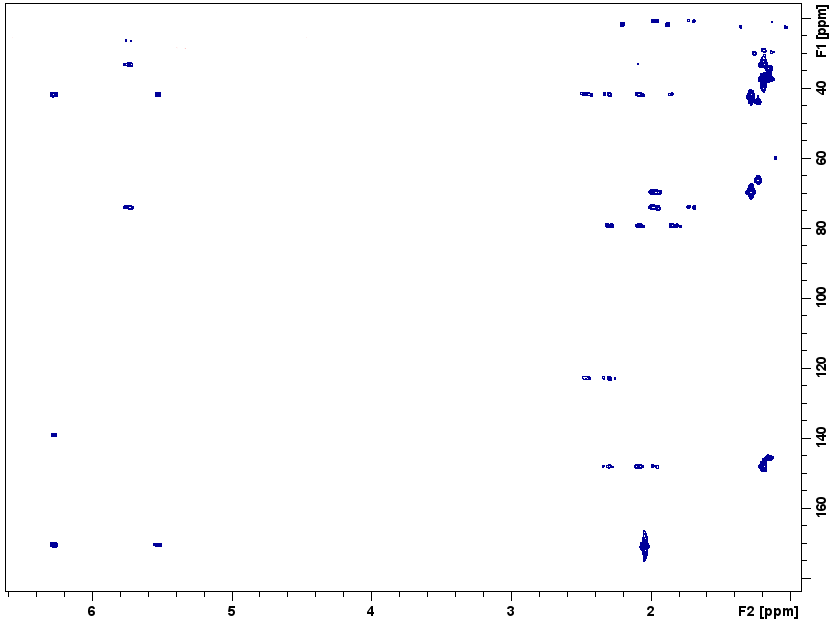

# **Figure S3-1.** ^1^H NMR spectrum of 8-epi-ivalbin (CDCl_3_, 400.13 MHz).


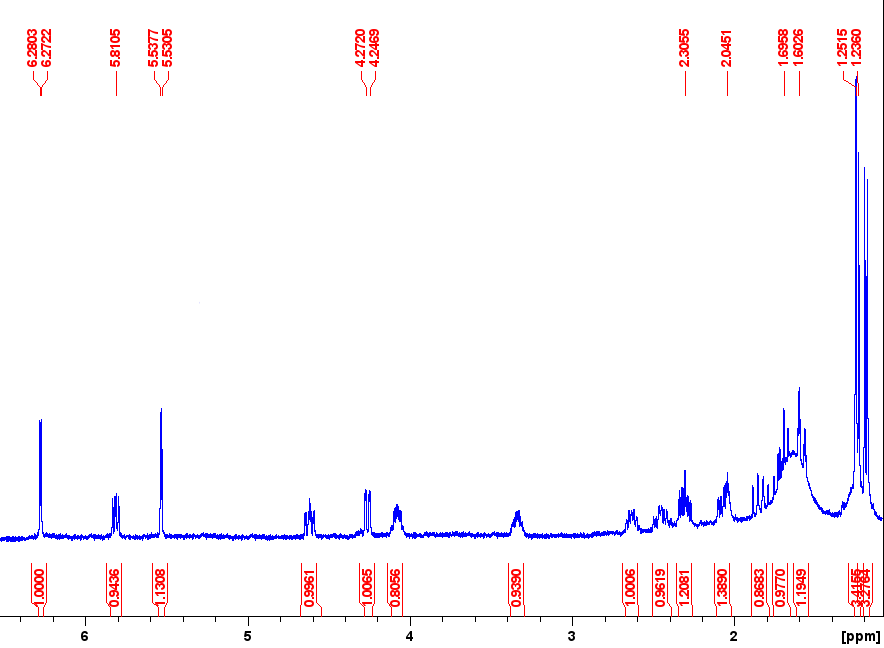

# **Figure S3-2**. COSY spectrum of 8-epi-ivalbin (CDCl_3_, 400.13 MHz).


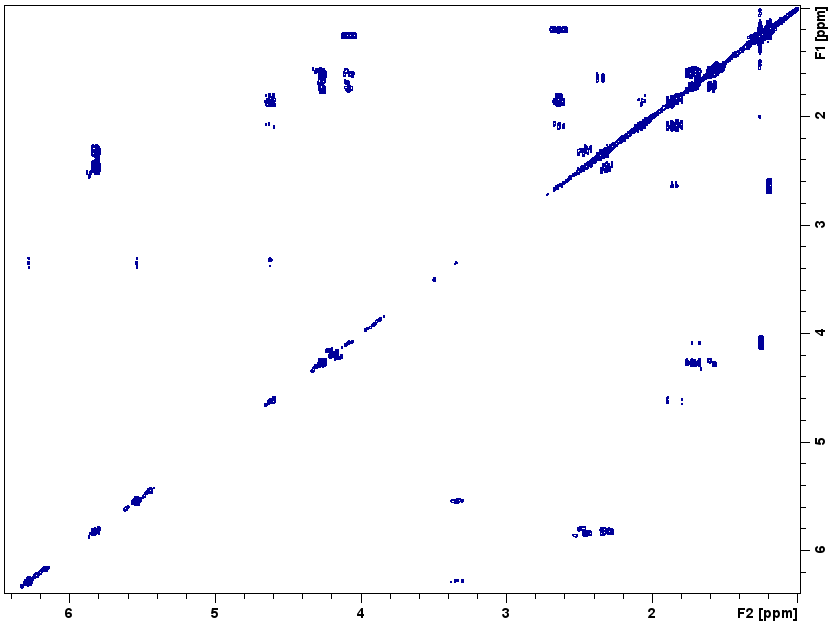

# **Figure S3-4**. HSQC spectrum of 8-epi-ivalbin (CDCl_3_, 400.13 MHz).


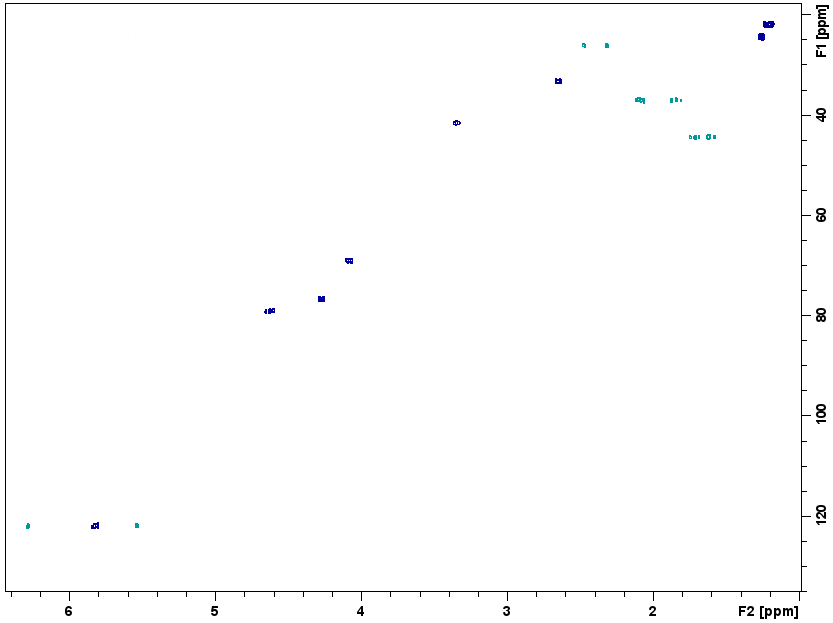

# **Figure S3-5**. HMBC spectrum of 8-epi-ivalbin (CDCl_3_, 400.13 MHz).


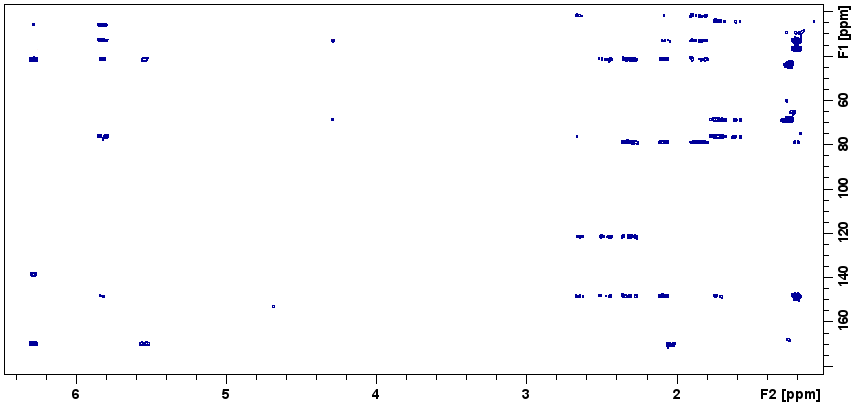

# **Figure S4-1.** ^1^H NMR spectrum of compound **1** (CDCl_3_, 400.13 MHz).


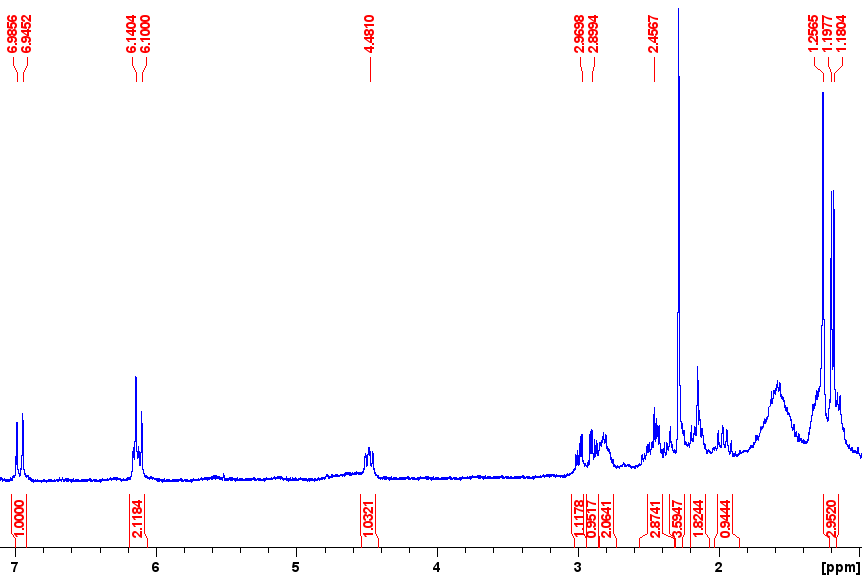

# **Figure S4-2**. COSY spectrum of compound **1** (CDCl_3_, 400.13 MHz).
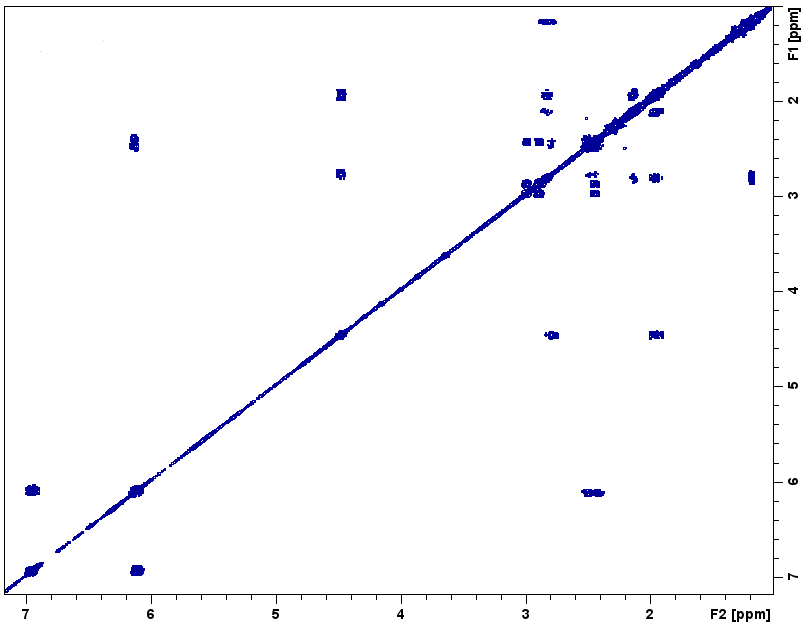

# **Figure S4-4**. HSQC spectrum of compound **1** (CDCl_3_, 400.13 MHz).
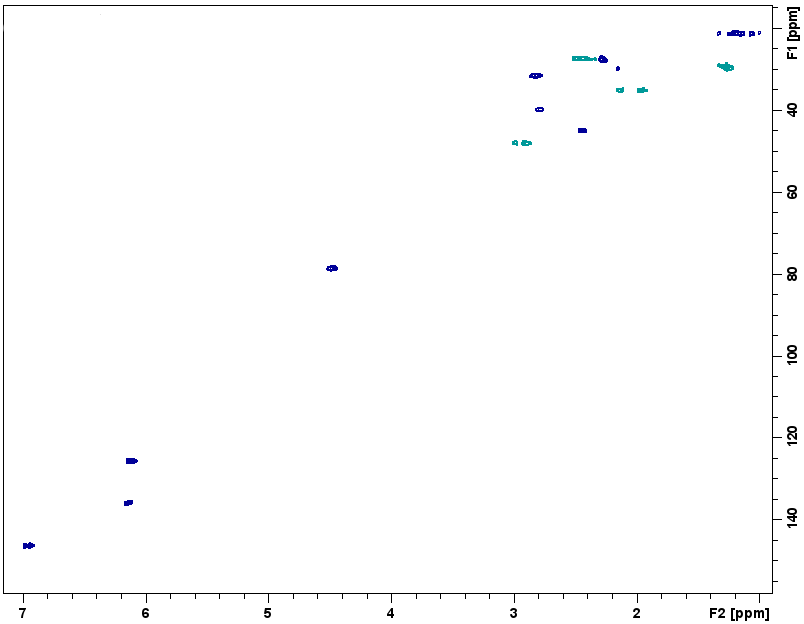

# **Figure S4-5**. HMBC spectrum of compound **1** (CDCl_3_, 400.13 MHz).


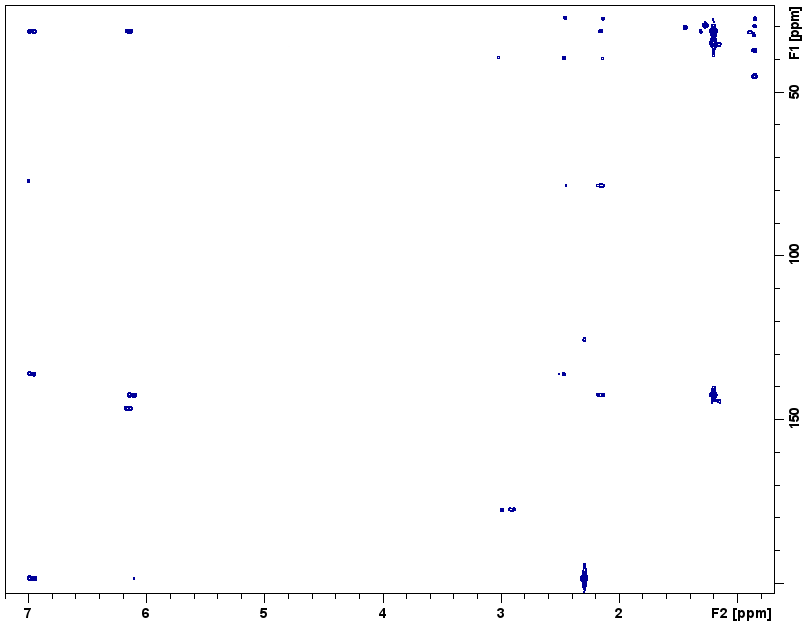

# **Figure S5-1.** ^1^H NMR spectrum of compound **2**(CDCl_3_, 400.13 MHz).


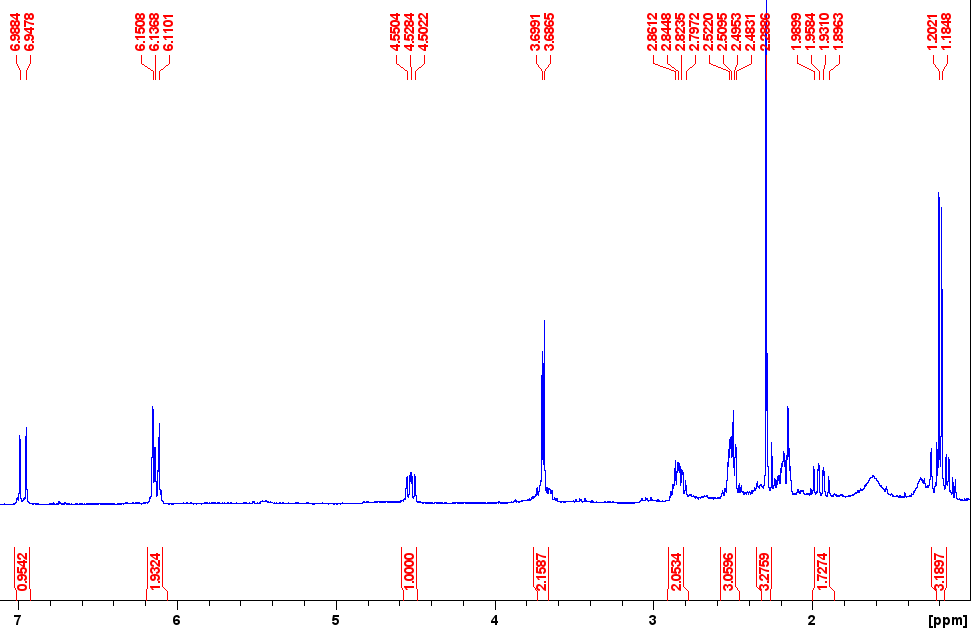

# **Figure S5-2**. COSY spectrum of compound **2** (CDCl_3_, 400.13 MHz).


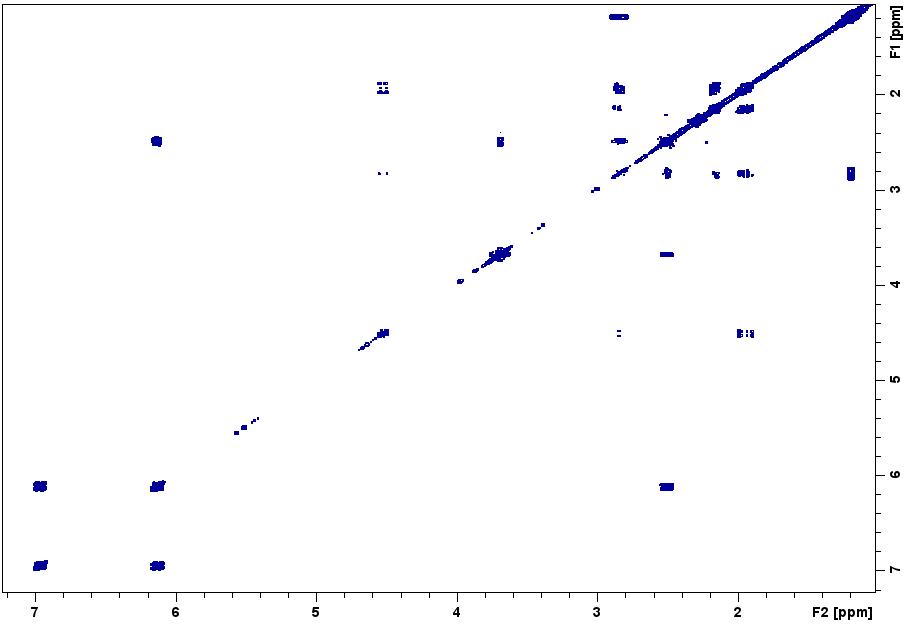

# **Figure S5-4**. HSQC spectrum of compound **2** (CDCl_3_, 400.13 MHz).


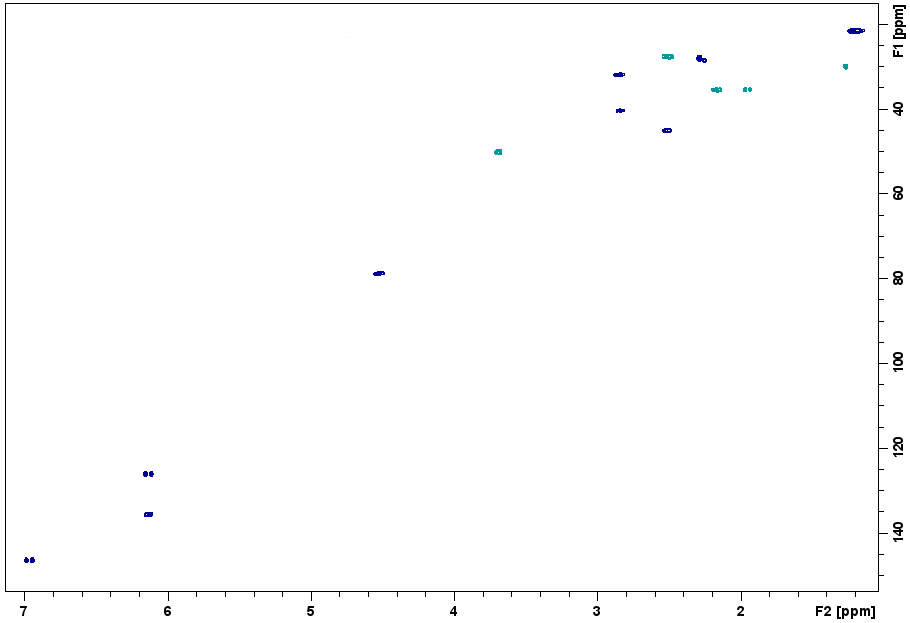

# **Figure S5-5**. HMBC spectrum of compound **2** (CDCl_3_, 400.13 MHz).


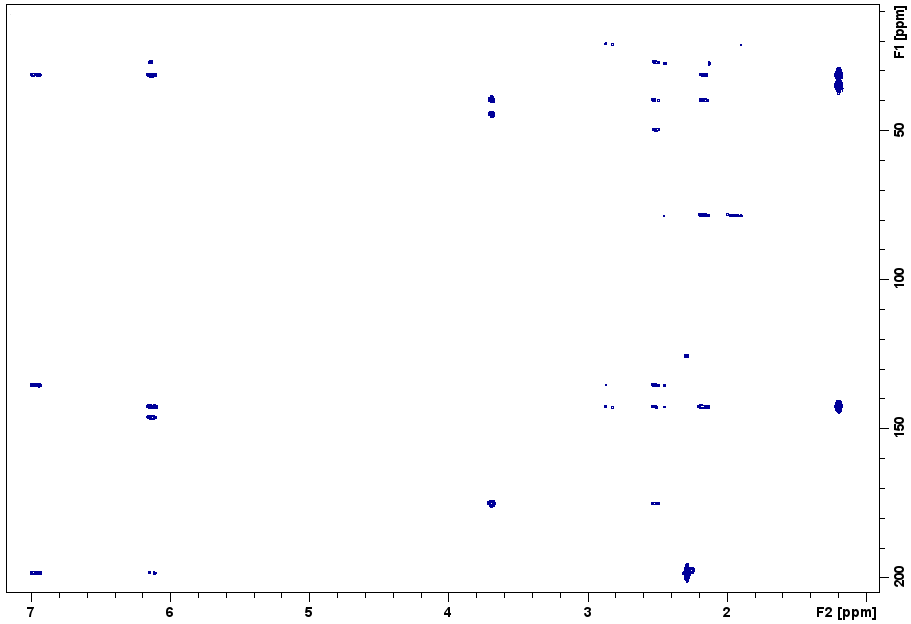

# **Figure S6-1.** ^1^H NMR spectrum of compound **3** (CDCl_3_, 400.13 MHz).

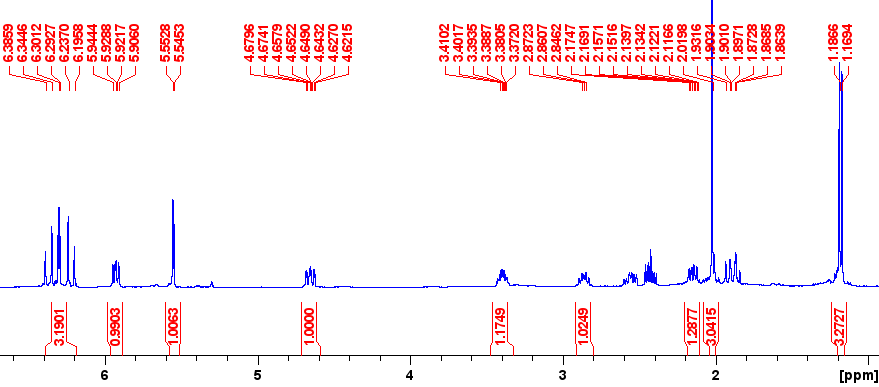


# **Figure S6-2**. COSY spectrum of compound **3** (CDCl_3_, 400.13 MHz).


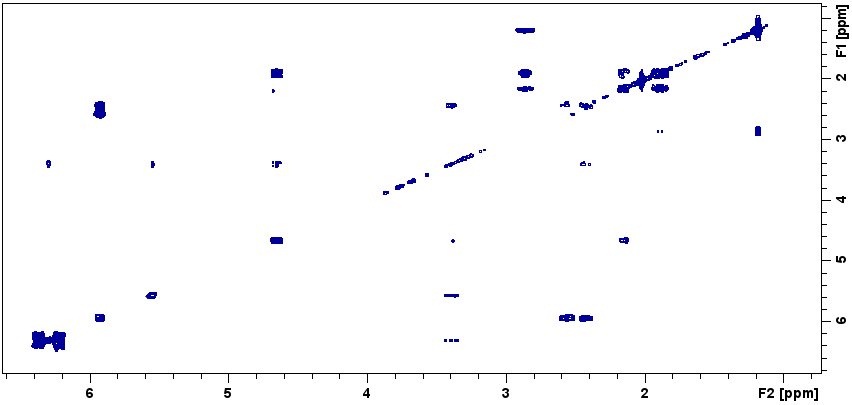

# **Figure S6-4**. HSQC spectrum of compound **3** (CDCl_3_, 400.13 MHz).


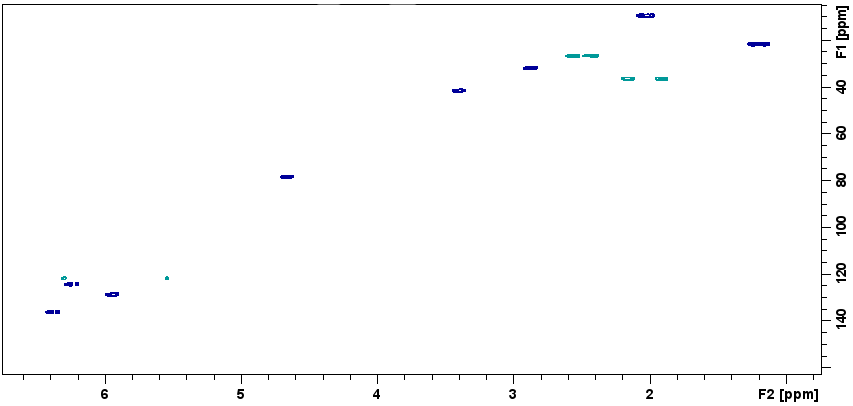

# **Figure S6-5**. HMBC spectrum of compound **3** (CDCl_3_, 400.13 MHz).
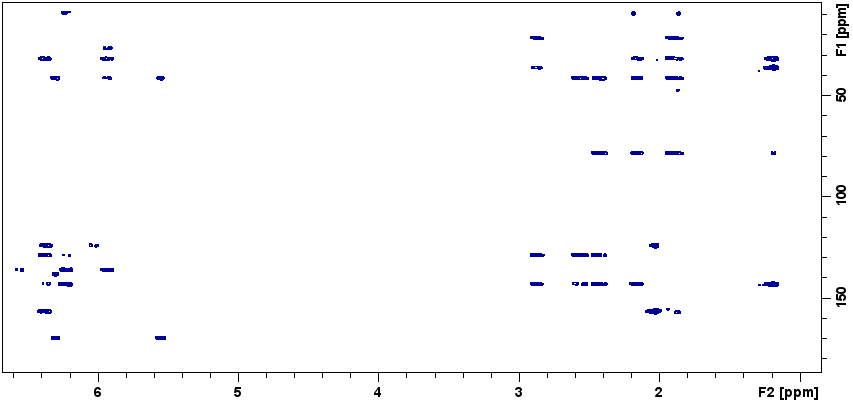

# **Figure S7-1.** ^1^H NMR spectrum of compound **3´** (CDCl_3_, 400.13 MHz).
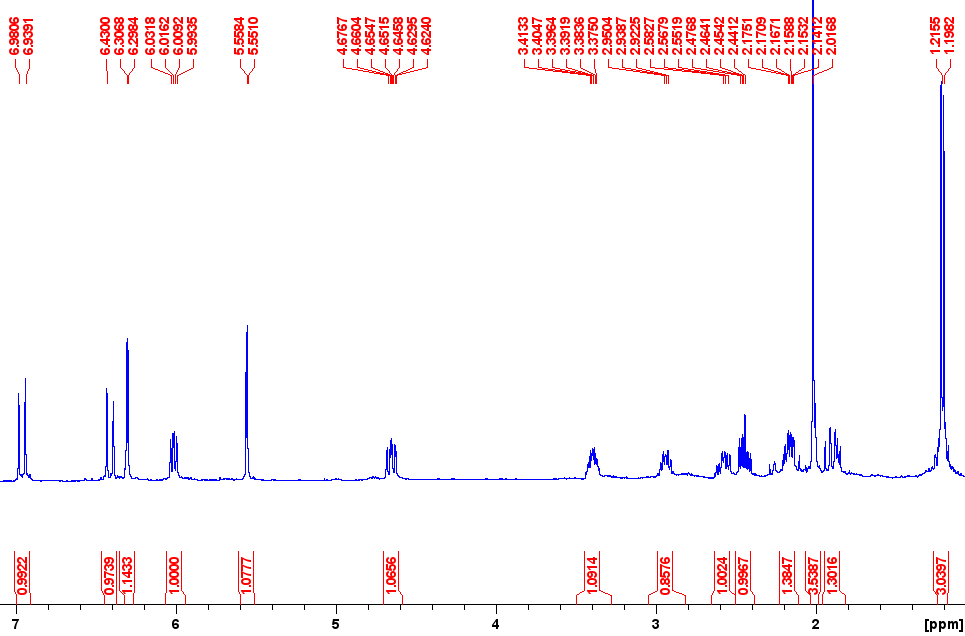

# **Figure S7-2**. COSY spectrum of compound **3´** (CDCl_3_, 400.13 MHz).


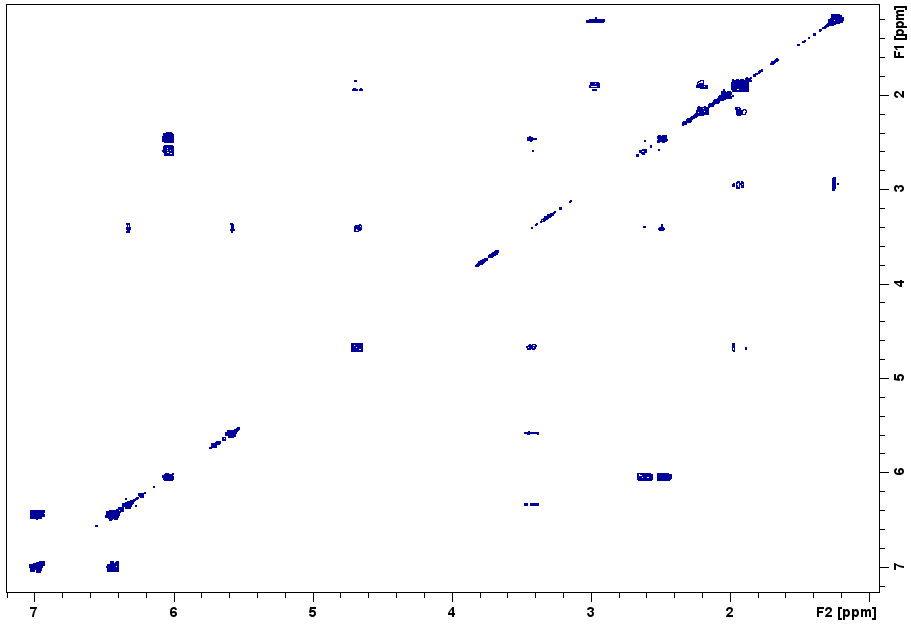

# **Figure S7-3**. HSQC spectrum of compound **3´** (CDCl_3_, 400.13 MHz).


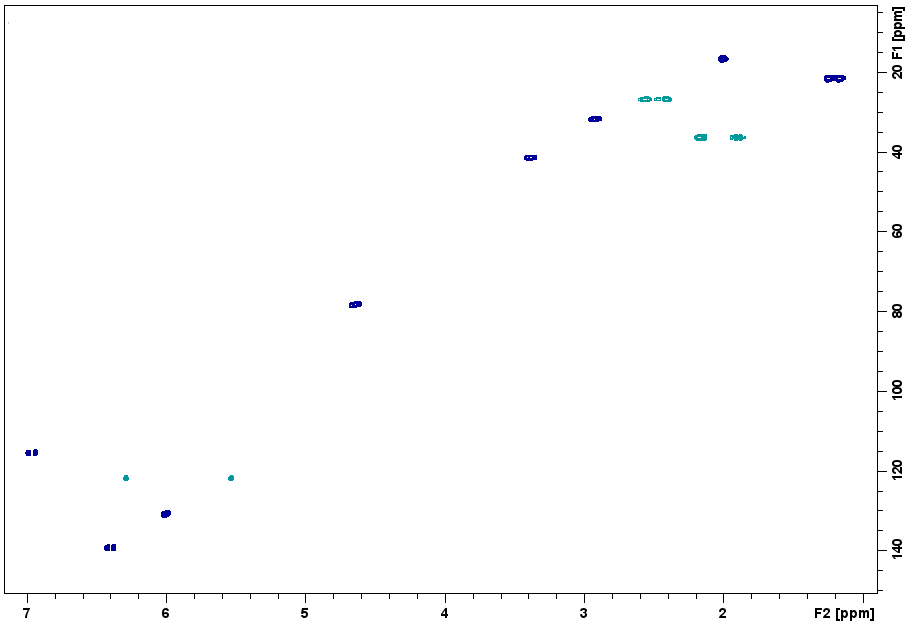

# **Figure S7-4**. HMBC spectrum of compound **3´** (CDCl_3_, 400.13 MHz).


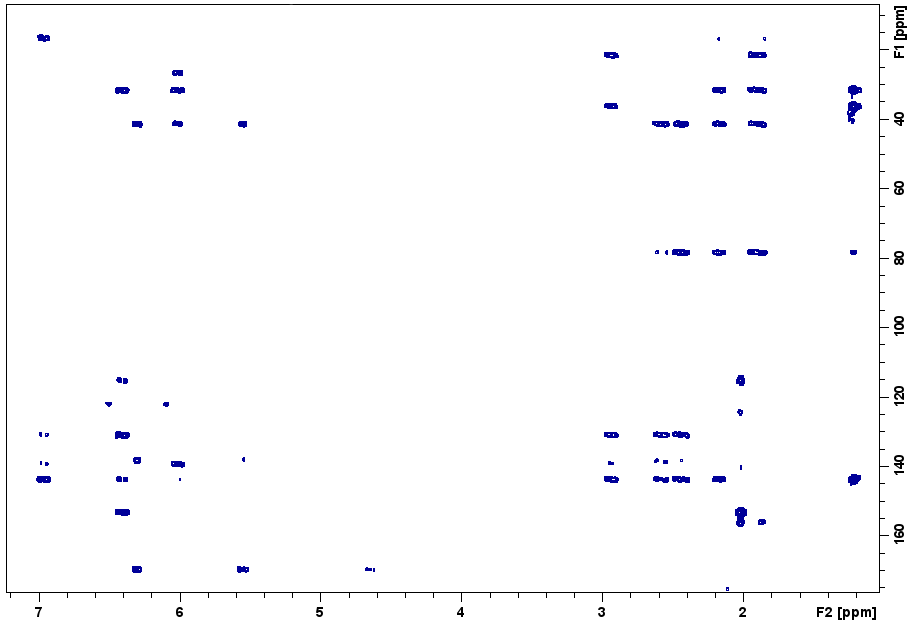

# **Figure S8-1.** ^1^H NMR spectrum of compound **4** (CDCl_3_, 400.13 MHz).


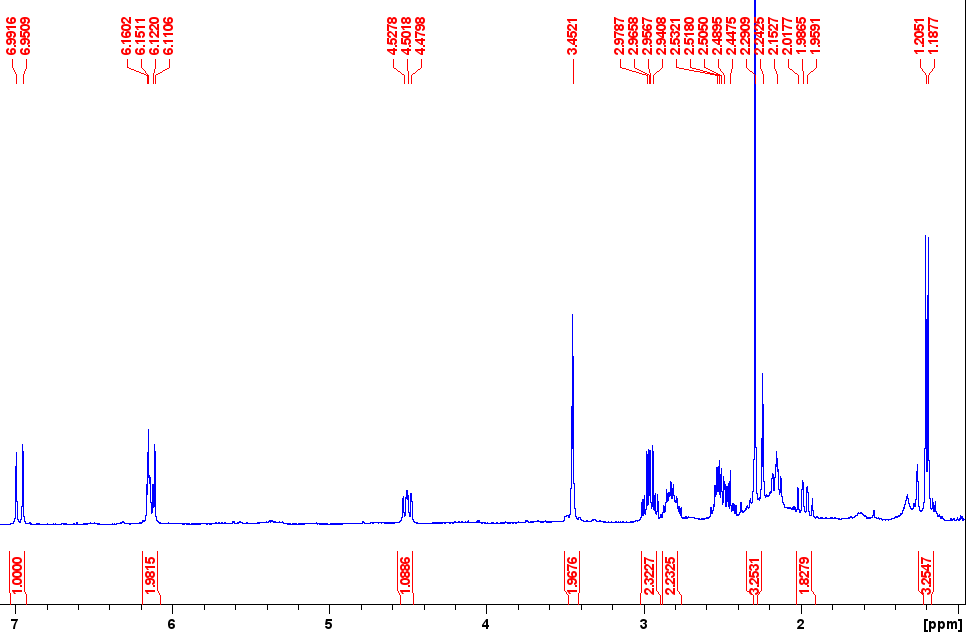

# **Figure S8-2**. COSY spectrum of compound **4** (CDCl_3_, 400.13 MHz).


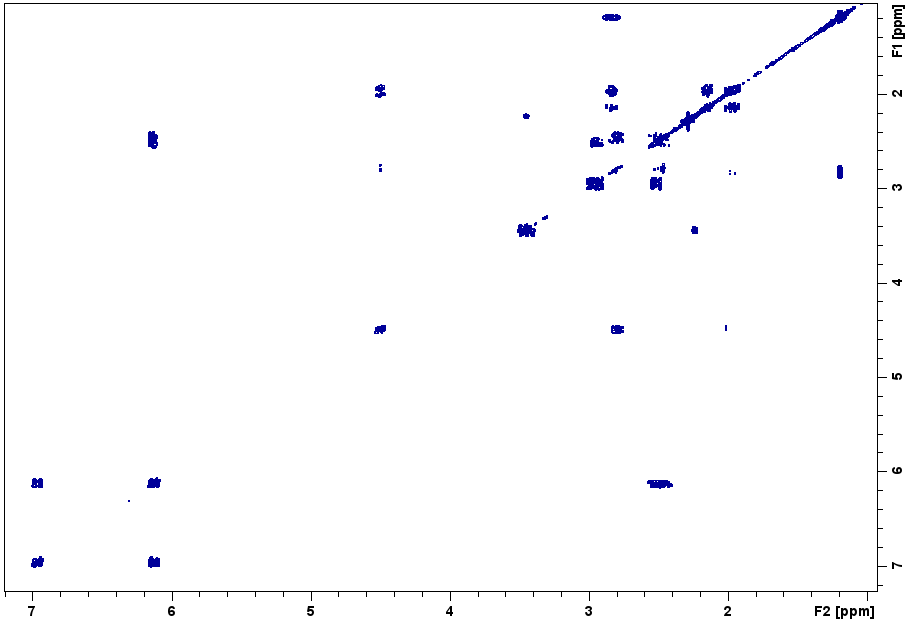

# **Figure S8-3**. HSQC spectrum of compound **4** (CDCl_3_, 400.13 MHz).


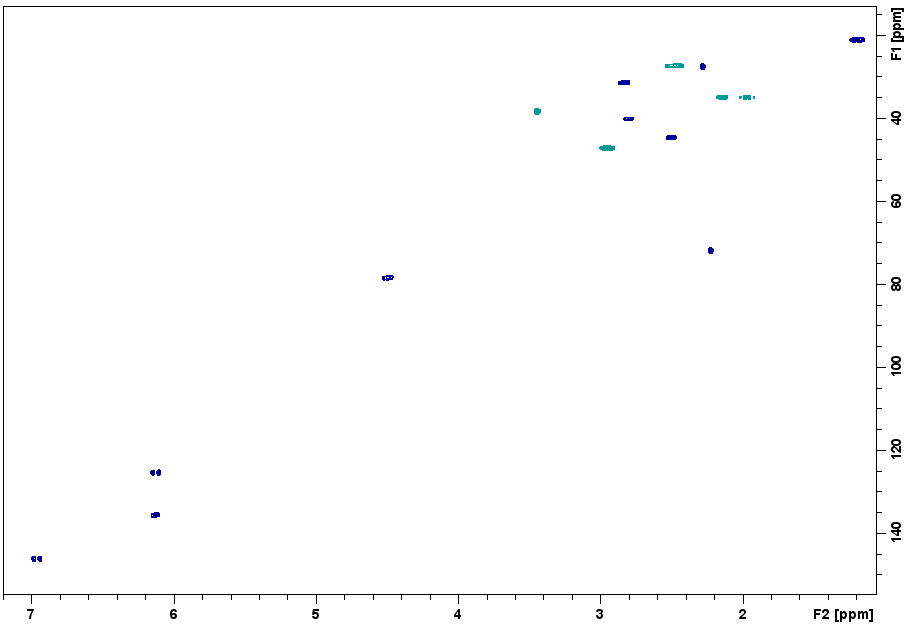

# **Figure S8-4**. HMBC spectrum of compound **4** (CDCl_3_, 400.13 MHz).

# **
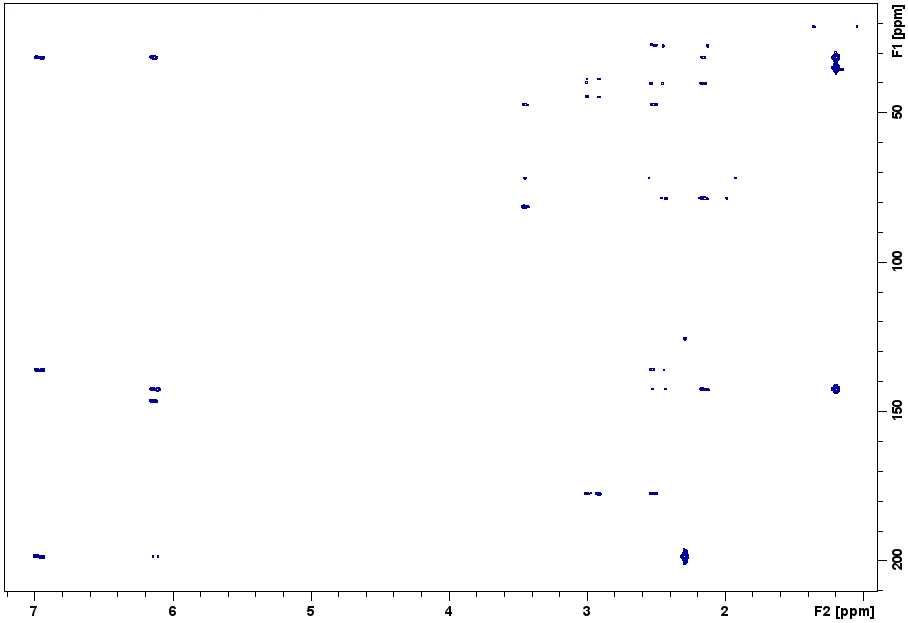
**

# **Figure S9-1.** ^1^H NMR spectrum of compound **5** (CDCl_3_, 400.13 MHz).


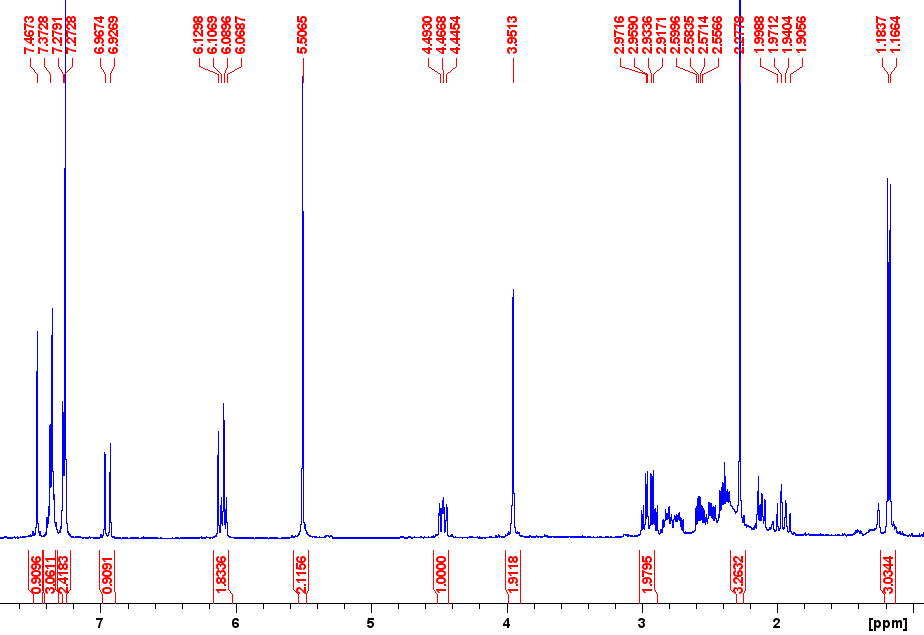

# **Figure S9-2**. COSY spectrum of compound **5** (CDCl_3_, 400.13 MHz).


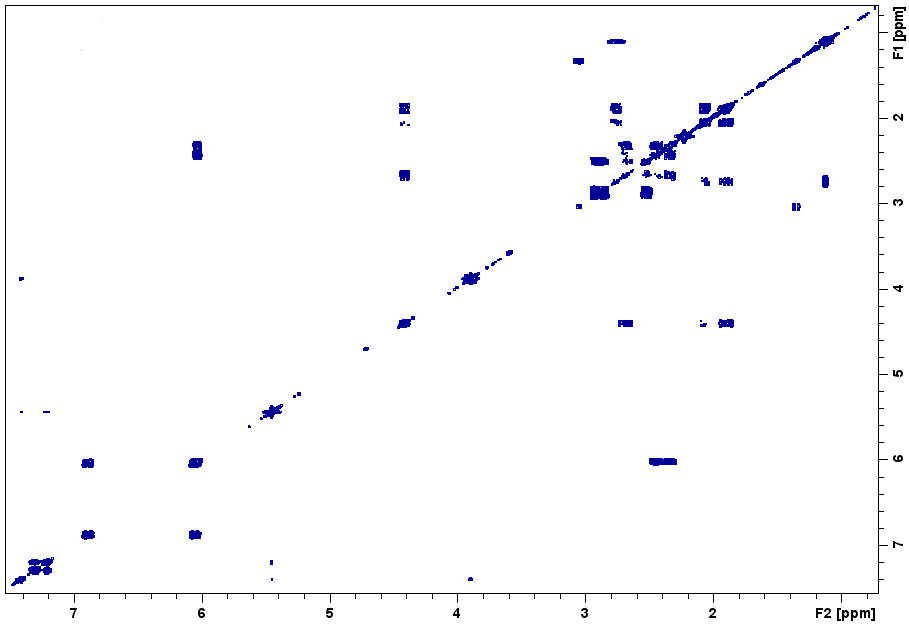

# **Figure S9-3**. HSQC spectrum of compound **5** (CDCl_3_, 400.13 MHz).


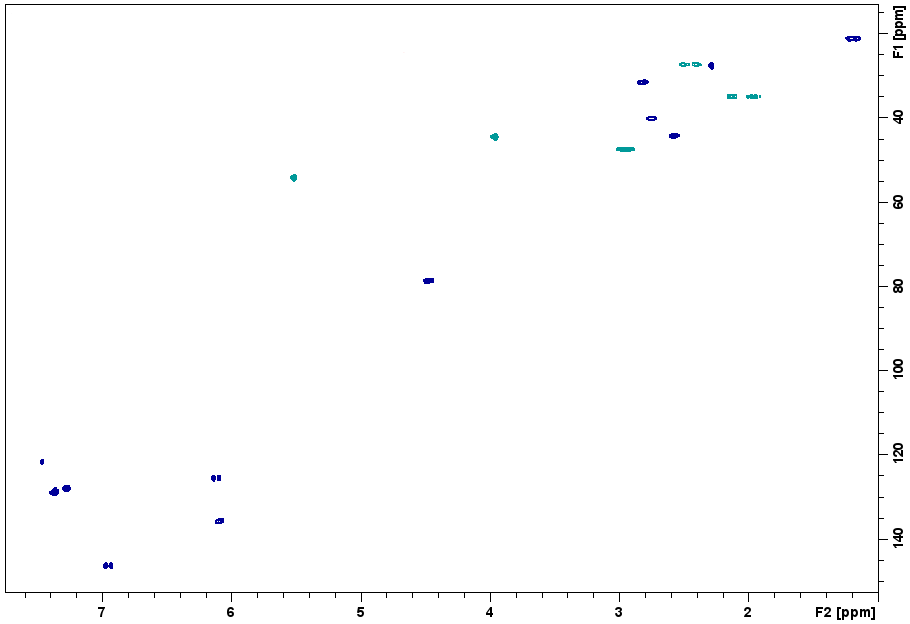

# **Figure S9-4**. HMBC spectrum of compound **5** (CDCl_3_, 400.13 MHz).


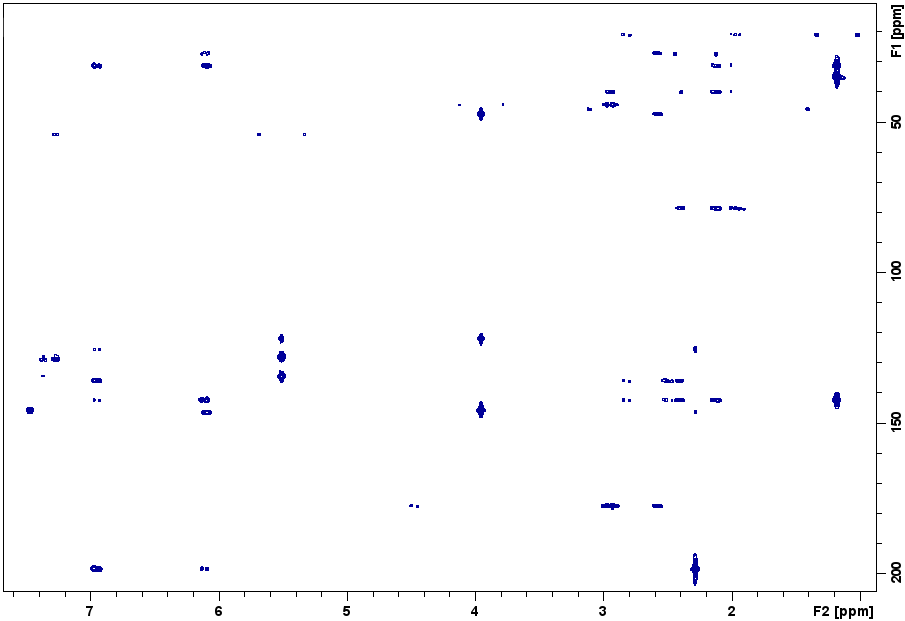

# **Figure S10-1.** ^1^H NMR spectrum of compound **6** (CDCl_3_, 400.13 MHz).


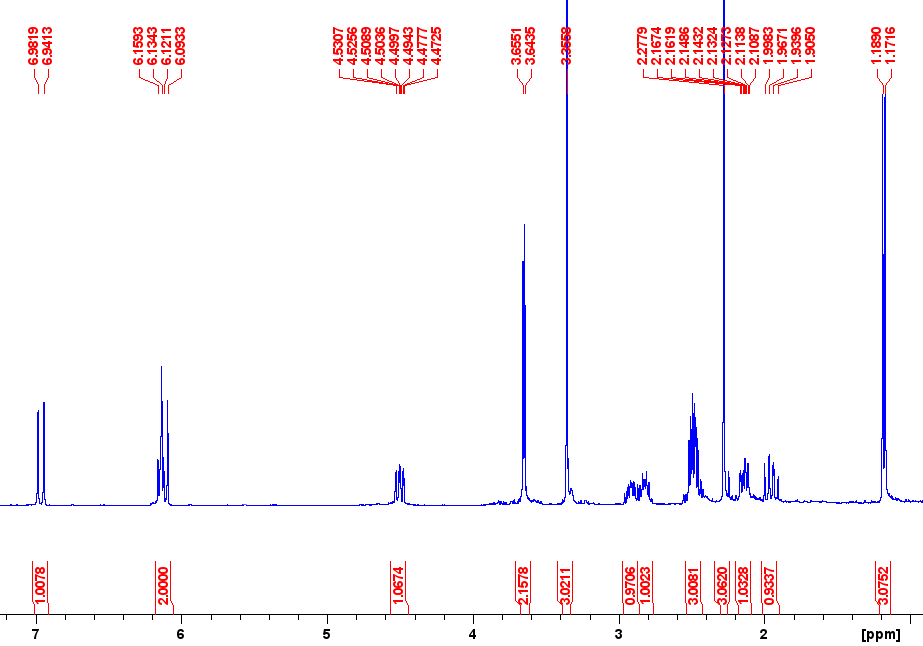

# **Figure S10-2**. COSY spectrum of compound **6** (CDCl_3_, 400.13 MHz).


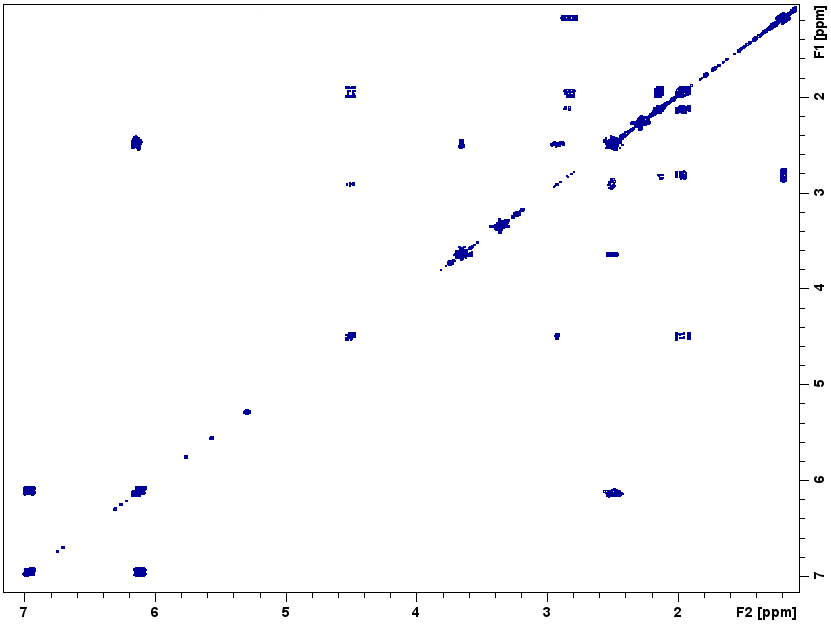

# **Figure S10-3**. HSQC spectrum of compound **6** (CDCl_3_, 400.13 MHz).


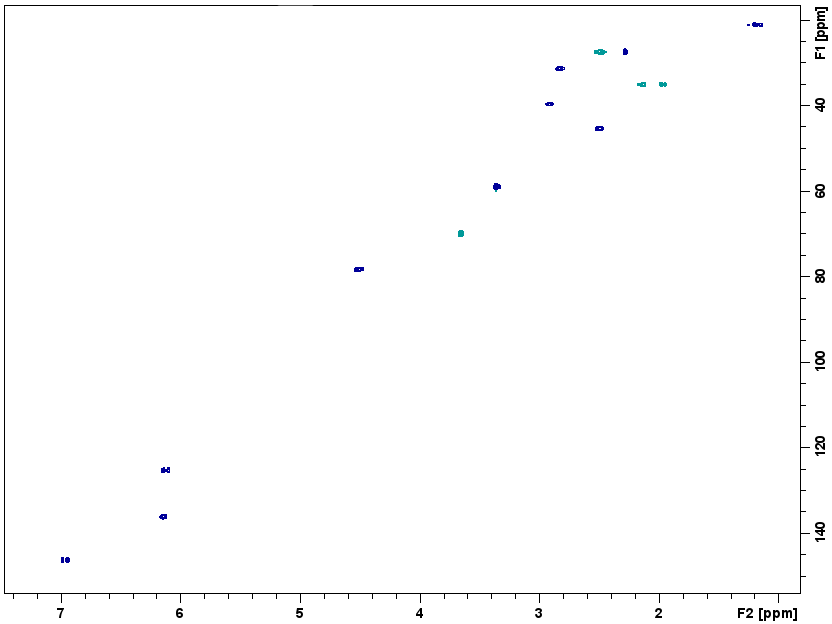

# **Figure S10-4**. HMBC spectrum of compound **6** (CDCl_3_, 400.13 MHz).


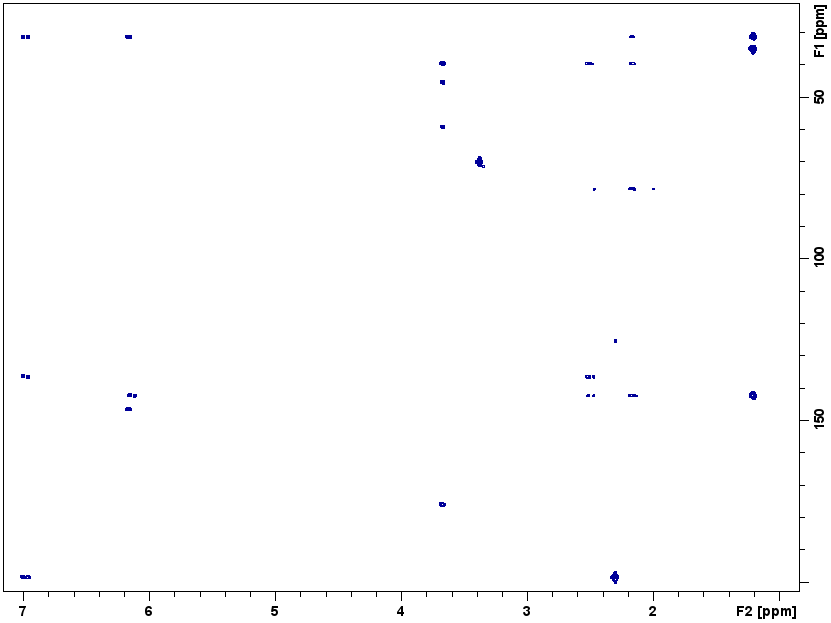

# **Figure S11-1.** ^1^H NMR spectrum of compound **7** (CDCl_3_, 400.13 MHz).


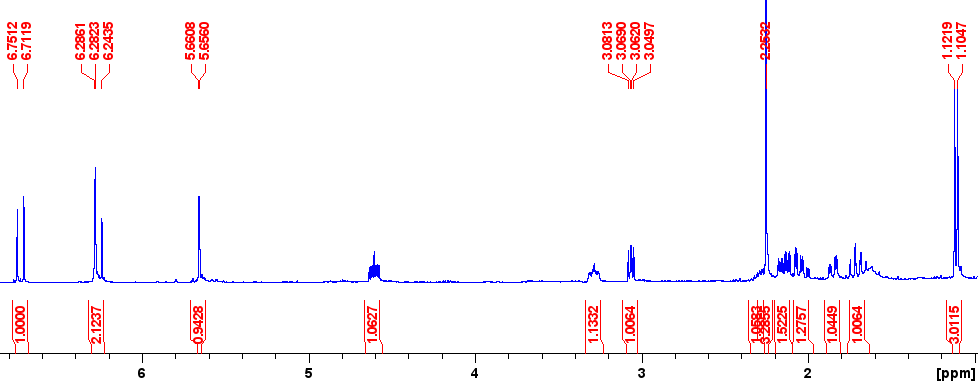

# **Figure S11-2**. COSY spectrum of compound **7** (CDCl_3_, 400.13 MHz).


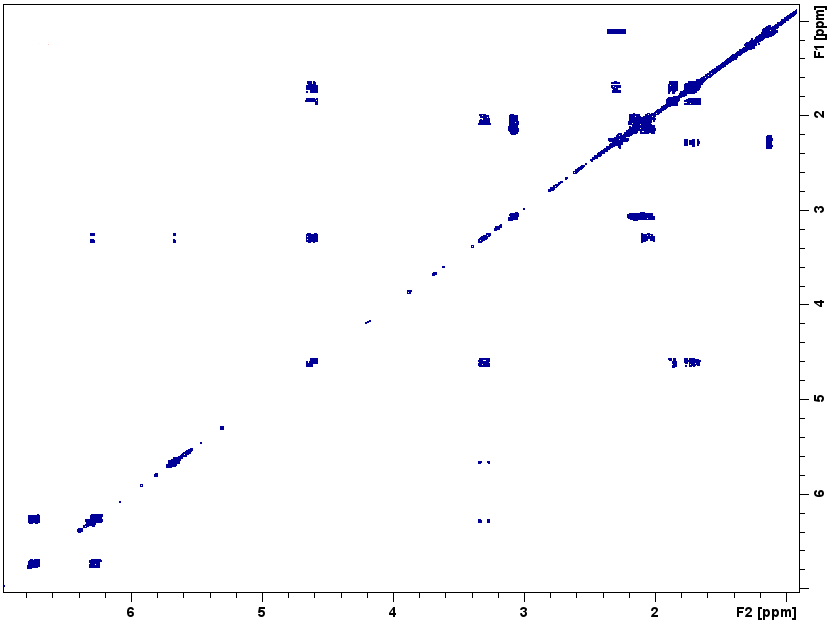

# **Figure S11-3**. HSQC spectrum of compound **7** (CDCl_3_, 400.13 MHz).
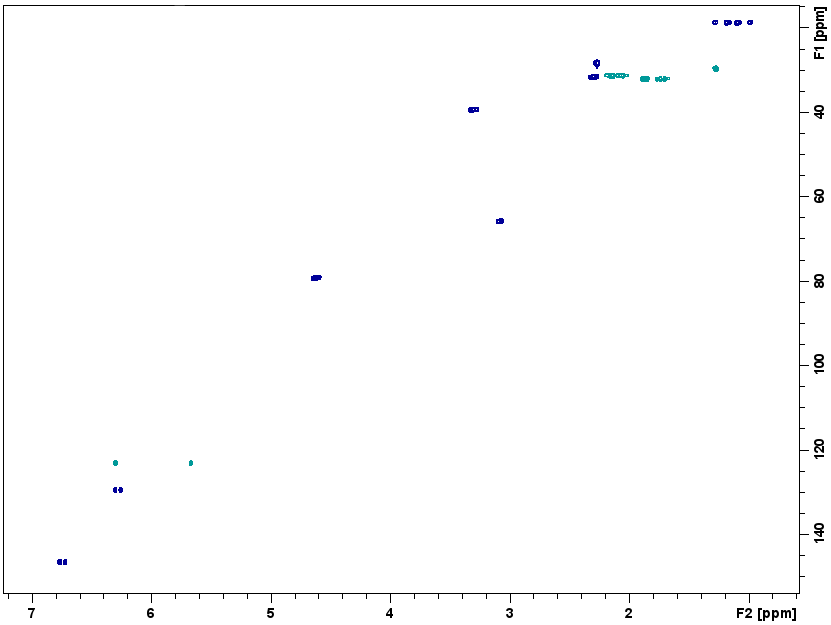

# **Figure S11-4**. HMBC spectrum of compound **7** (CDCl_3_, 400.13 MHz).


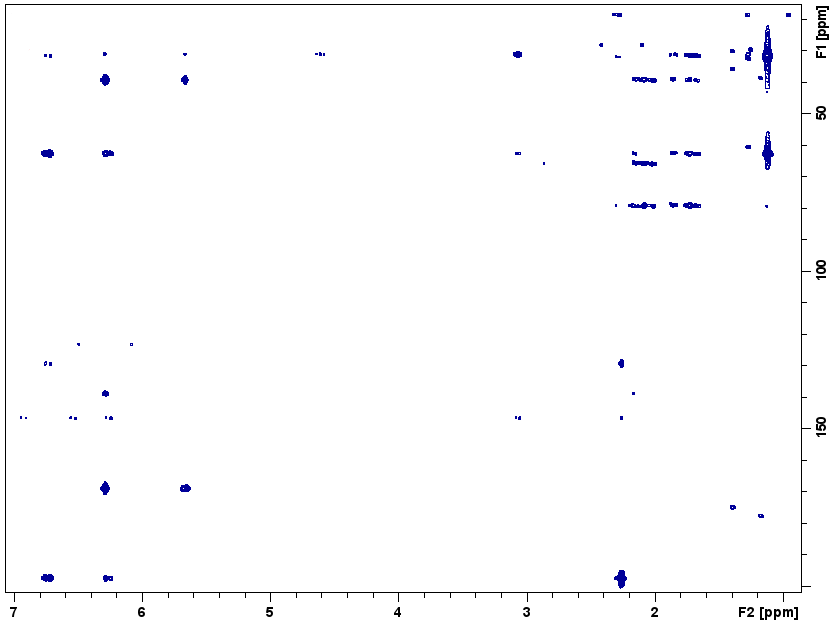

# **Figure S12-1.** ^1^H NMR spectrum of compound **8** (CDCl_3_, 400.13 MHz).


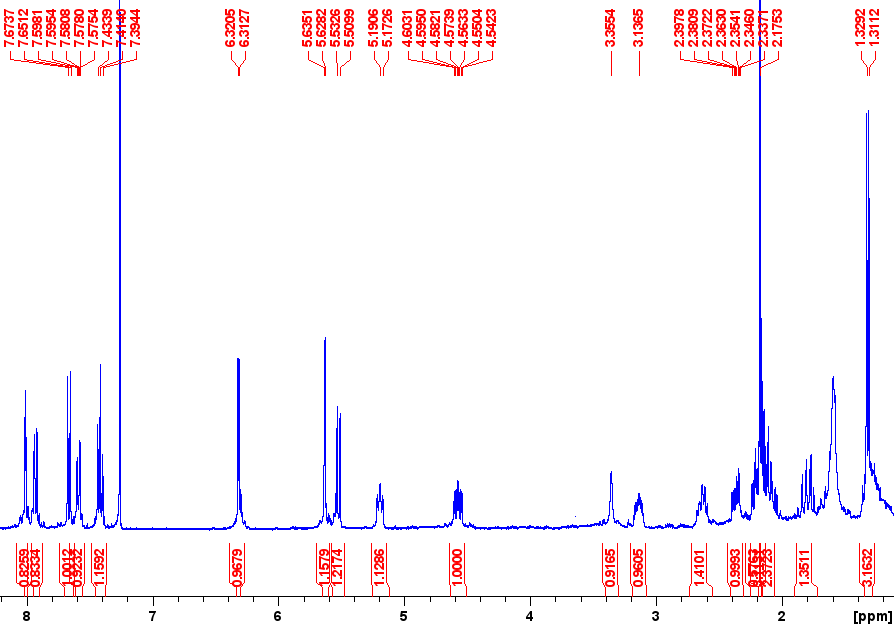

# **Figure S12-2**. COSY spectrum of compound **8** (CDCl_3_, 400.13 MHz).


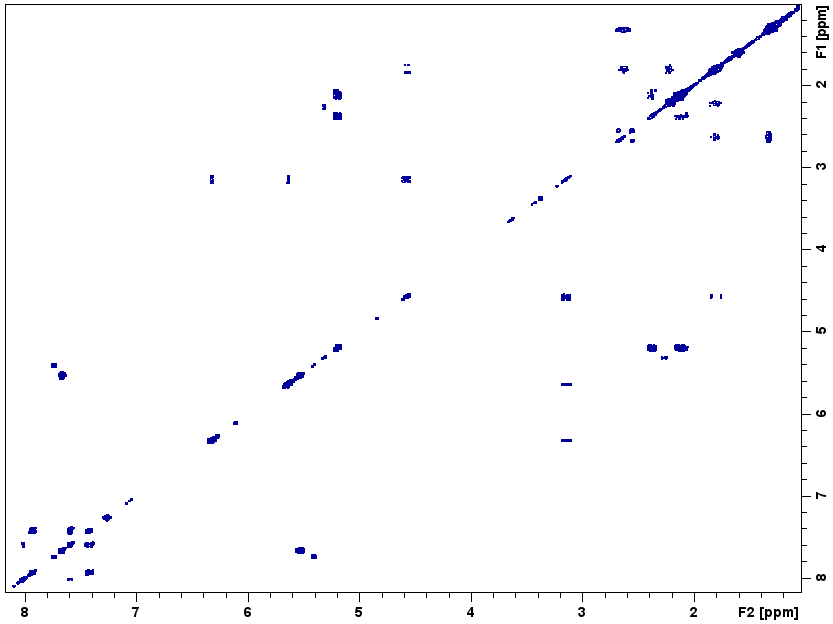

# **Figure S12-3**. HSQC spectrum of compound **8** (CDCl_3_, 400.13 MHz).


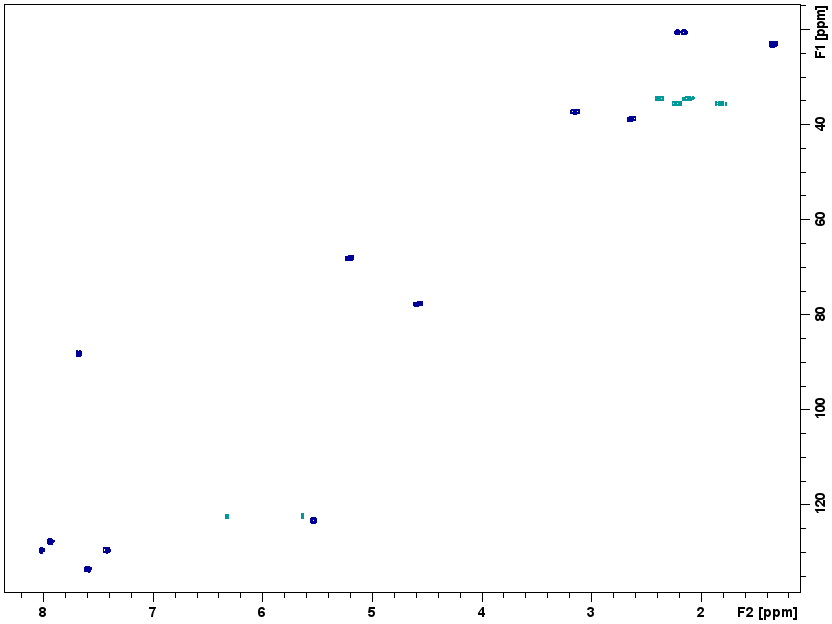

# **Figure S12-4**. HMBC spectrum of compound **8** (CDCl_3_, 400.13 MHz).


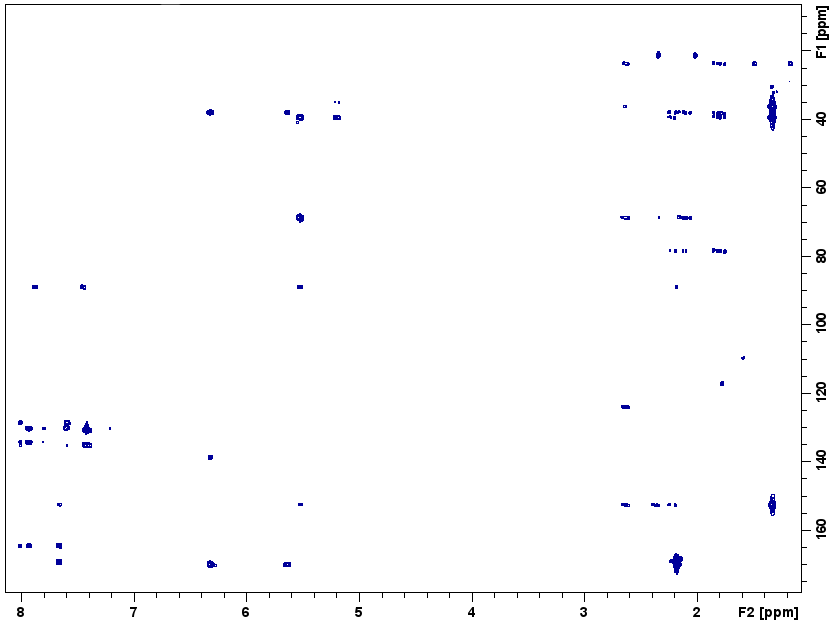

# **Figure S13-1.** ^1^H NMR spectrum of compound **9** (CDCl_3_, 400.13 MHz).


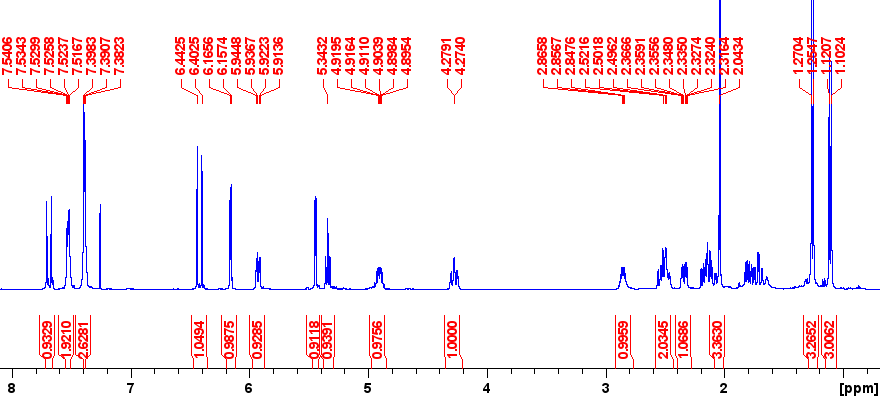

# **Figure S13-2**. COSY spectrum of compound **9** (CDCl_3_, 400.13 MHz).


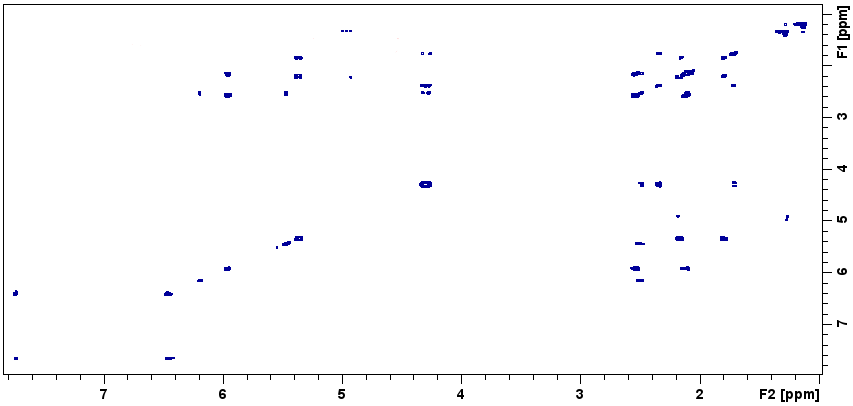

# **Figure S13-3**. HSQC spectrum of compound **9** (CDCl_3_, 400.13 MHz).


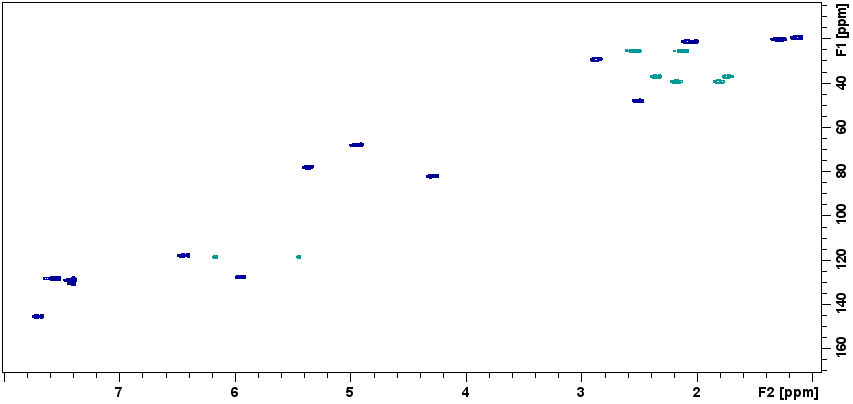

# **Figure S13-4**. HMBC spectrum of compound **9** (CDCl_3_, 400.13 MHz).


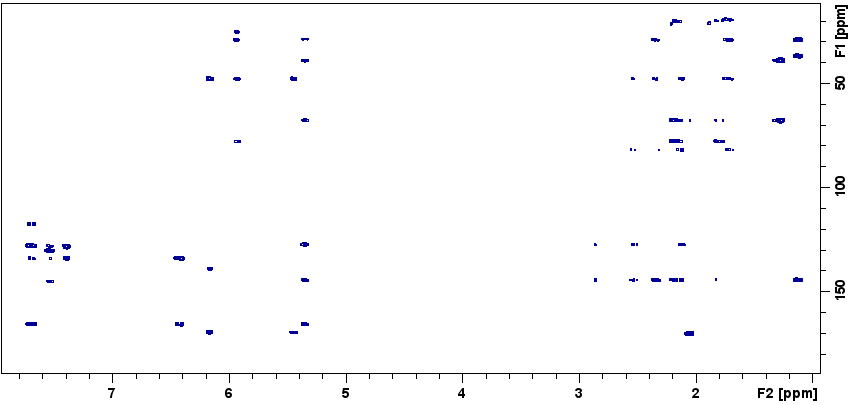

# **Figure S14-1.** ^1^H NMR spectrum of compound **10** (CDCl_3_, 400.13 MHz).


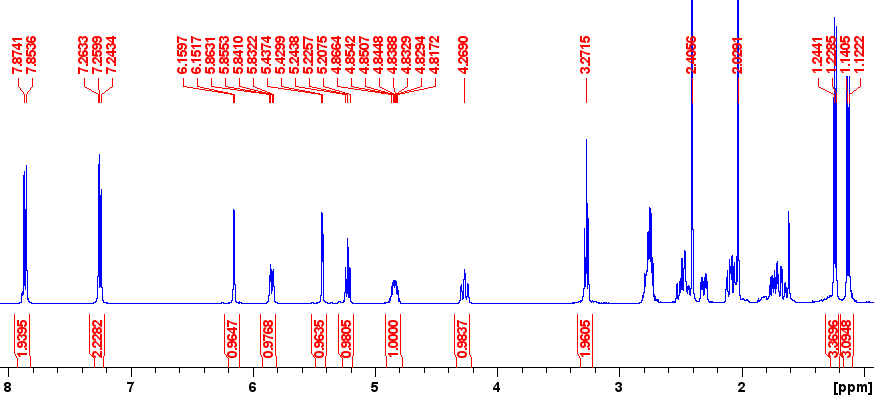

# **Figure S14-2**. COSY spectrum of compound **10** (CDCl_3_, 400.13 MHz).


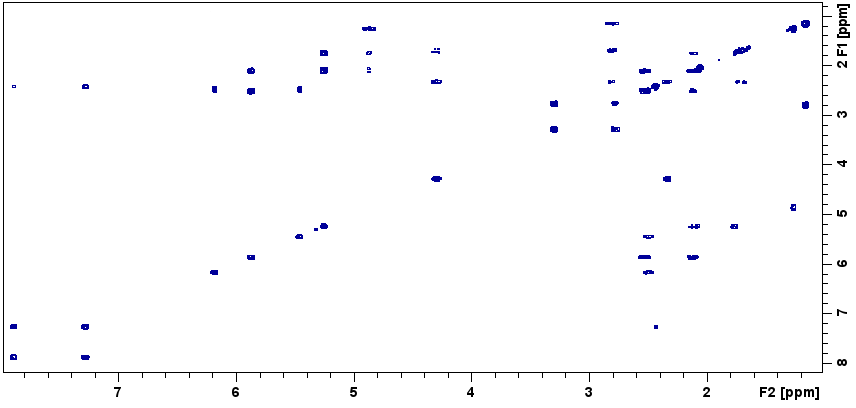

# **Figure S14-3**. HSQC spectrum of compound **10** (CDCl_3_, 400.13 MHz).


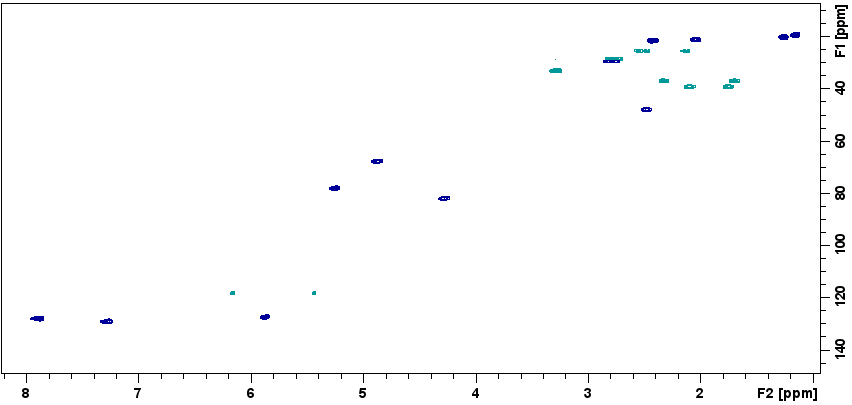

# **Figure S14-4**. HMBC spectrum of compound **10** (CDCl_3_, 400.13 MHz).


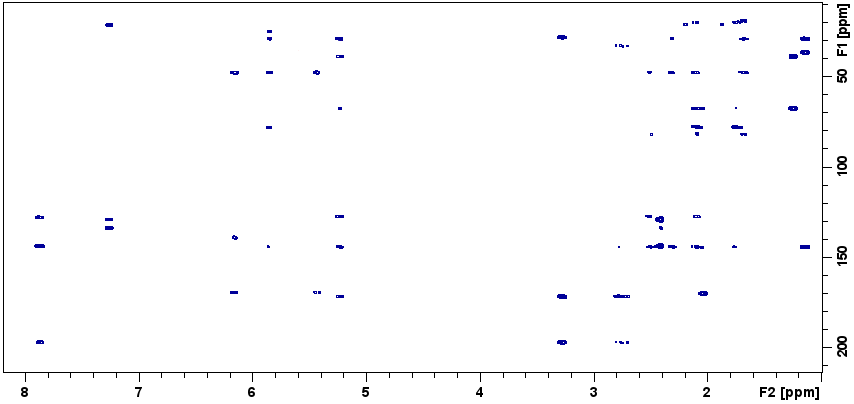

Supplement: Supplementary file 1 — Additional file 1. [file 13659_2025_513_MOESM1_ESM.docx]
